# Association of Behavioral and Clinical Risk Factors With Cataract: A Two-Sample Mendelian Randomization Study

**DOI:** 10.1167/iovs.64.10.19

**Published:** 2023-07-17

**Authors:** Chen Jiang, Ronald B. Melles, Poorab Sangani, Thomas J. Hoffmann, Pirro G. Hysi, M. Maria Glymour, Eric Jorgenson, Salil A. Lachke, Hélène Choquet

**Affiliations:** 1 Kaiser Permanente Northern California (KPNC), Division of Research, Oakland, California, United States; 2 KPNC, Department of Ophthalmology, Redwood City, California, United States; 3 KPNC, Department of Ophthalmology, South San Francisco, California, United States; 4 Institute for Human Genetics, UCSF, San Francisco, California, United States; 5 Department of Epidemiology and Biostatistics, UCSF, San Francisco, California, United States; 6 King's College London, Section of Ophthalmology, School of Life Course Sciences, London, United Kingdom; 7 King's College London, Department of Twin Research and Genetic Epidemiology, London, United Kingdom; 8 University College London, Great Ormond Street Hospital Institute of Child Health, London, United Kingdom; 9 Regeneron Genetics Center, Tarrytown, New York, United States; 10 Department of Biological Sciences, University of Delaware, Newark, Delaware, United States; 11 Center for Bioinformatics and Computational Biology, University of Delaware, Newark, Delaware, United States

**Keywords:** cataract, mendelian randomization, cataract risk factors, genetic epidemiology

## Abstract

**Purpose:**

To investigate the association of genetically determined primary open-angle glaucoma (POAG), myopic refractive error (RE), type 2 diabetes (T2D), blood pressure (BP), body mass index (BMI), cigarette smoking, and alcohol consumption with the risk of age-related cataract.

**Methods:**

To assess potential causal effects of clinical or behavioral factors on cataract risk, we conducted two-sample Mendelian randomization analyses. Genetic instruments, based on common genetic variants associated with risk factors at genome-wide significance (*P* < 5 × 10^−8^), were derived from published genome-wide association studies (GWAS). For age-related cataract, we used GWAS summary statistics from our previous GWAS conducted in the Genetic Epidemiology Research on Adult Health and Aging (GERA) cohort (28,092 cataract cases and 50,487 controls; all non-Hispanic whites) or in the UK Biobank (31,852 cataract cases and 428,084 controls; all European-descent individuals). We used the inverse-variance weighted (IVW) method as our primary source of Mendelian randomization estimates and conducted common sensitivity analyses.

**Results:**

We found that genetically determined POAG and mean spherical equivalent RE were significantly associated with cataract risk (IVW model: odds ratio [OR] = 1.04; 95% confidence interval [CI], 1.01–1.08; *P* = 0.018; per diopter more hyperopic: OR = 0.92; 95% CI, 0.89–0.93; *P* = 6.51 × 10^−13^, respectively). In contrast, genetically determined T2D, BP, BMI, cigarette smoking, or alcohol consumption were not associated with cataract risk (*P* > 0.05).

**Conclusions:**

Our results provide evidence that genetic risks for POAG and myopia may be causal risk factors for age-related cataract. These results are consistent with previous observational studies reporting associations of myopia with cataract risk. This information may support population cataract risk stratification and screening strategies.

Cataract is a leading cause of blindness worldwide and in the United States, affecting 22% of Americans 40 years and older.^1^ Surgery is the current standard of management,^2^^,^^3^ and, although cataract surgery is usually effective as measured by improvements in visual acuity,^3^ intraoperative or postoperative complications can occur.^2^^,^^4^^,^^5^ Twin and family studies strongly support an important role for genetic factors in cataract risk with heritability estimates up to 58%.^6^^–^^11^ Recently, we conducted a multiethnic genome-wide association study (GWAS) meta-analysis of cataract that identified 55 genetic risk loci.^12^ Although this work revealed novel genetic loci associated with cataract, there is still a need to discover the causal pathways underlying cataractogenesis.

Previous observational studies have identified clinical and behavioral risk factors for cataract, including type 2 diabetes (T2D), high blood pressure (BP), high body mass index (BMI), myopic refractive error (RE), cigarette smoking, and alcohol consumption.^2^^,^^13^^–^^31^ However, it is not clear if these observational associations represent causal risk factors. In our recent study, we estimated the pairwise genetic correlations (*r_g_*) between cataract and more than 700 diseases/traits from different publicly available resources/consortia and identified strong genetic correlations between cataract and several of these factors, including myopia, BMI, and cigarette smoking.^12^ We also found a significant genetic correlation between cataract and glaucoma.^12^ These findings suggest considerable shared genetic influences between cataract and those ophthalmic, clinical, and behavioral factors; however, the nature of the relationships remain largely unexplored.

The Mendelian randomization (MR) approach uses genetic variants to determine whether an observational association between a risk factor and an outcome is consistent with a causal effect.^32^^–^^35^ This approach leverages the random segregation of alleles that are not affected by environmental conditions. Results from MR studies are typically consistent with those of randomized controlled trials and provide, for example, evidence for drug target validation.^36^ Although this approach has been successful in many causal inference discoveries,^37^^–^^39^ only a few MR studies evaluating whether observational associations of clinical and behavioral factors with cataract risk are consistent with causal effects have been reported.^40^^–^^43^ Furthermore, the results of prior MR studies for cataract have been inconsistent.^42^^,^^43^

Here, we employed a two-sample MR approach^44^ to assess the potential causal role of primary open-angle glaucoma (POAG), myopic RE, T2D, BP, BMI, cigarette smoking, and alcohol consumption on the risk of cataract. We compare genetic effect estimates for those exposures and cataract risk (outcome) obtained through GWAS summary statistics, in particular using our large GWAS of cataract conducted using the Genetic Epidemiology Research on Adult Health and Aging (GERA) cohort and UK Biobank.^12^ The assessment of these risk factors could have public health and clinical implications for prevention and early detection of this common cause of visual disability.

## Methods

### Study Design

**Figure 1.**
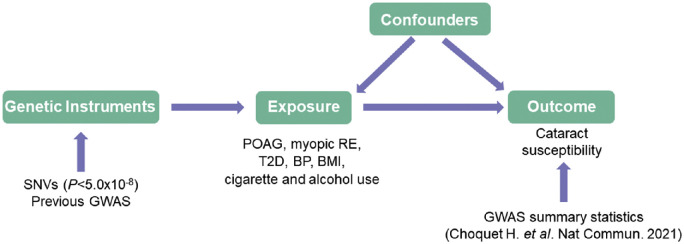
Conceptual framework of the MR study. Common genetic variants that are associated with the exposure (e.g., POAG risk) at genome-wide significance (*P* < 5.0 × 10^−8^) in published GWASs were used as genetic instruments. The association of those genetic instruments with the outcome (i.e., cataract risk) is only mediated through the exposure.

Two-sample MR analyses were conducted to investigate the association of genetically determined POAG, myopic RE, T2D, blood pressure (i.e., systolic and diastolic), BMI, cigarette smoking (i.e., smoking initiation, cigarettes per day, and lifetime smoking), and alcohol consumption (i.e., drinks per week) with the risk of age-related cataract. For each of the exposures, we used the lead single nucleotide variations (SNVs) previously reported as being genome-wide significant (*P* < 5.0 × 10^−8^) as a set of genetic instruments. Genetic instruments were then clumped using a window of 10 Mb and maximal linkage disequilibrium of *r*^2^ = 0.001 between instruments to ensure that genetic variants were independent. The conceptual framework of the current MR study is reported in Figure 1, and the different datasets used for this MR study are summarized in Supplementary Table S1.

### GWAS Summary Statistics for Cataract

Genetic association data for cataract risk (outcome) were retrieved from our previous GWAS study conducted in the GERA cohort.^12^ The GERA cohort consists of 110,266 adult members of the Kaiser Permanente Medical Care Plan, Northern California Region (KPNC), an integrated healthcare delivery system that includes ongoing longitudinal electronic health records.^45^^,^^46^ The Institutional Review Board of the Kaiser Foundation Research Institute approved all study procedures. Written informed consent was obtained from all participants. In the current study, we retrieved genetic association data from the GWAS of cataract conducted in 28,092 cataract cases and 50,487 controls (all GERA participants were of European ancestry).^12^ Briefly, GERA patients with pseudophakia were diagnosed by a Kaiser Permanente ophthalmologist and were identified in the KPNC electronic health record system based on the following International Classification of Disease, Ninth Revision (ICD9) or Tenth Revision (ICD10) diagnosis codes: V43.1 (ICD-9 code) and Z96.1 (ICD-10 code). GERA cataract cases were also identified if they had a history of having a cataract surgery at KPNC. Our GERA control group included all of the non-cases. To make sure that study samples for exposures did not overlap with those for the outcome, in some MR analyses (i.e., association of POAG, BP, or BMI with cataract risk), genetic association data for cataract risk were retrieved from our previous GWAS study conducted in the UK Biobank (UKB).^12^ The UKB cataract cases (*N* = 31,852 individuals of European ancestry) were defined as participants with a self-reported cataract operation (f20004 code 1435) or/and a hospital record including a diagnosis code (ICD-10: H25 or H26); the UKB controls (*N* = 428,084 individuals of European ancestry) were participants who were not cases.

### Genetic Instruments for POAG

Genetic association data for POAG risk (exposure) were retrieved from our recent large GWAS meta-analysis of POAG conducted by the International Glaucoma Genetics Consortium.^47^ GWAS summary statistics for this study^47^ have been publicly accessible via GWAS Catalog under study accession identifier GCST90011767 (corresponding to all European ancestry cohorts except UKB; *N* = 15,229 POAG cases and 177,473 controls). After clumping, a total of 46 genetic instruments for POAG were used for the MR analyses (Supplementary Table S2).

### Genetic Instruments for Refractive Error

Genetic variants as instrumental variables for RE (exposure) were extracted from a GWAS conducted in 102,117 UKB participants of European ancestry with direct refraction measurement and part of our previously reported European ancestry meta-analysis.^48^ In UKB, RE was measured directly using the Tomey RC 5000 Auto-Refractor Keratometer (Tomey Corporation, Aichi, Japan). The spherical equivalent was estimated as the spherical RE (UKB codes 5084 and 5085) plus half the cylindrical error (UKB codes 5086 and 5087) for each eye. The mean of spherical equivalent was then used as the outcome of the GWAS analysis.^48^ After clumping, a total of 166 genetic instruments for mean spherical equivalent RE were used for the MR analyses (Supplementary Table S3).

### Genetic Instruments for Type 2 Diabetes

Genetic variants as instrumental variables for T2D (exposure) were extracted from a large GWAS analysis consisting of 19,119 T2D cases and 423,698 controls, all UKB participants of European ancestry.^49^ After clumping, a total of 66 genetic instruments for T2D were used for the MR analyses (Supplementary Table S4).

### Genetic Instruments for Blood Pressure

Genetic variants as instrumental variables for blood pressure (exposure) were extracted from our large GWAS conducted in 80,792 GERA participants of European ancestry with systolic and diastolic blood pressure measurements.^50^ After clumping, a total of 20 genetic instruments for systolic blood pressure and a total of 20 genetic instruments for diastolic blood pressure were used for the MR analyses (Supplementary Tables S5 and S6).

### Genetic Instruments for BMI

Genetic variants as instrumental variables for BMI (exposure) were extracted from our large GWAS meta-analysis consisting of 315,347 individuals of European ancestry from the GERA and the Genetic Investigation of Anthropomorphic Traits consortium.^51^ GWAS summary statistics for this study^51^ were publicly accessible via the GWAS Catalog under study accession identifier GCST006368. After clumping, a total of 153 genetic instruments for BMI were used for the MR analyses (Supplementary Table S7).

### Genetic Instruments for Cigarette Smoking

Genetic variants as instrumental variables for cigarette smoking initiation (ever having smoked regularly vs. never) and amount smoked (number of cigarettes per day) were extracted from the most recent GWAS and Sequencing Consortium of Alcohol and Nicotine use (GSCAN) study.^52^ GWAS summary statistics for smoking initiation and amount smoked analyses^52^ included 805,431 and 326,497 individuals of European ancestry, respectively. Those GWAS summary statistics are publicly accessible at https://conservancy.umn.edu/handle/11299/241912. We also used genetic variants as instrumental variables for lifetime smoking (represented by an index that captures smoking status, duration, heaviness, and cessation) from a GWAS conducted in 462,690 UKB participants of European ancestry.^53^ GWAS summary statistics for this study^53^ were publicly accessible via the GWAS Catalog under study accession identifier GCST009096. After clumping, a total of 231 genetic instruments for smoking initiation, 47 for cigarettes per day, and 121 for lifetime smoking were used for the MR analyses (Supplementary Tables S8–S10).

### Genetic Instruments for Alcohol Consumption

Genetic variants as instrumental variables for alcohol consumption (i.e., number of drinks per week as the exposure) were extracted from the most recent GSCAN study.^52^ GWAS summary statistics corresponding to the drinks per week analysis^52^ included 619,011 individuals of European ancestry, after removing GERA participants. After clumping, a total of 93 genetic instruments for drinks per week were used for the MR analyses (Supplementary Table S11).

### Two-Sample MR Analyses

All analyses were conducted in the R V.4.1.2 (R Foundation for Statistical Computing, Vienna, Austria) using the TwoSampleMR package.^44^ This package makes causal inference about an exposure on an outcome using GWAS summary statistics, generates linkage disequilibrium pruning of exposure SNVs and harmonizes exposure and outcome datasets. We used the inverse-variance weighted (IVW) method as our primary source of MR estimates. This IVW method essentially translates to a weighted regression of SNV outcome effects on SNV-exposure effects where the intercept is constrained to zero. Moreover, we reported the estimations from MR weighted median, weighted mode, and Mendelian randomization–Egger (MR-Egger). Further, leave-one-SNV-out analyses were conducted (Supplementary Tables S12–S21).

### Sensitivity Analyses

The potential effect of pleiotropy was evaluated by the regression intercept from the MR-Egger method,^54^ and Cochran *Q* tests were used to evaluate the presence of global heterogeneity among the effects of the genetic instruments.^55^ The MR-PRESSO (Mendelian Randomization Pleiotropy RESidual Sum and Outlier) method^55^^,^^56^ was also used to provide an MR estimate that is robust against the presence of heterogeneity among SNV effects and to re-assess the MR estimate after excluding outlier SNVs. Finally, we applied the MR-Steiger method,^57^ which removes variants from the analysis if their association with the outcome is stronger than that with the exposure.^55^

### Multivariable MR for POAG Adjusting for the Effect of RE on Cataract Risk

Recent MR studies^37^^,^^58^ suggested that lower (more myopic) RE is associated with POAG risk. Moreover, some genetic loci exhibited pleiotropic effects with RE/myopia, POAG, and cataract.^12^ Because these relationships can potentially bias the relationship between POAG and cataract, we conducted a multivariable MR analysis^59^^,^^60^ to adjust for the potential effect of RE. Genetic variants as instrumental variables for mean spherical equivalent RE were extracted from a GWAS conducted in 59,094 GERA non-Hispanic white participants.^37^ Single nucleotide polymorphisms (SNPs) achieving genome-wide significance for each trait (e.g., RE, POAG) were included in the multivariable MR analysis. After clumping, a total of 43 genetic instruments were used for the multivariable MR analysis adjusting for the effect of RE. The multivariable MR analysis was performed by jointly fitting the SNP–POAG and SNP–RE effect sizes simultaneously in the regression model on the SNP–cataract association. The multivariable MR analysis was conducted using the mv_multiple() function available in the TwoSampleMR package in R curated in the MR-Base platform.^44^

## Results

### MR Analyses Identify Association of Ophthalmic Conditions With Cataract Risk

**Figure 2.**
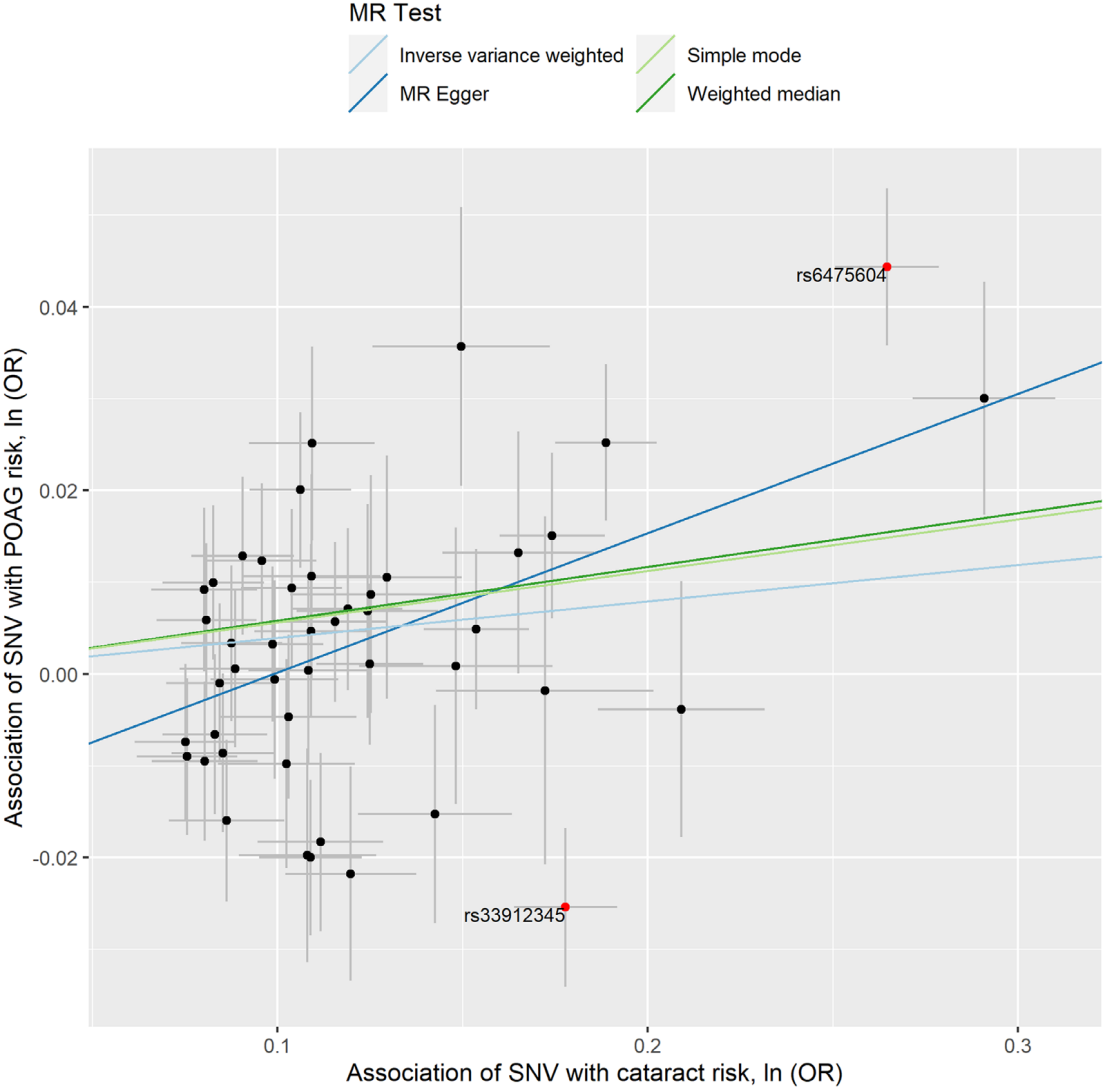
Association of POAG-associated variants with the risk of cataract. The *x*-axis shows 46 genetic instruments for POAG and their effect size estimates (ORs) with POAG. The *y*-axis shows the association of the same variants with cataract risk. The MR IVW regression line is plotted. The outlier genetic variants rs6475604 and rs33912345 detected by the MR-PRESSO model are labeled in *red*.

**Figure 3.**
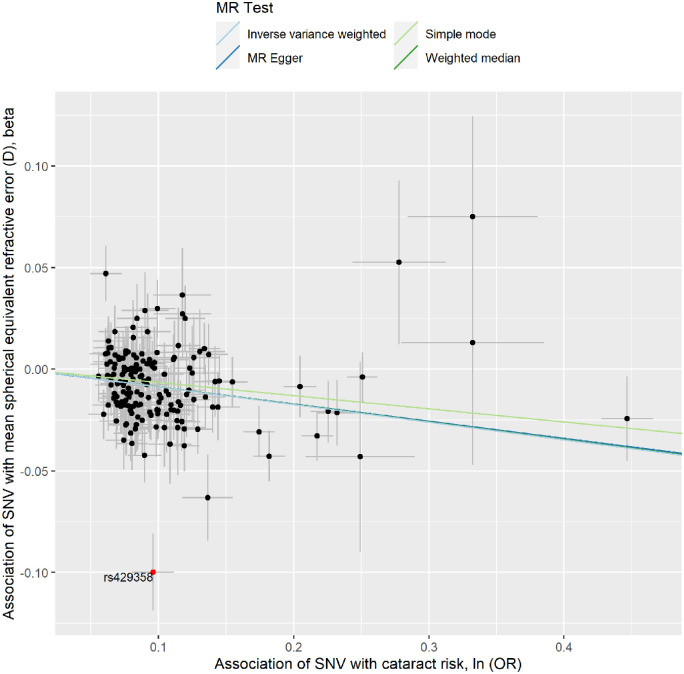
Association of refractive error-associated variants with the risk of cataract. The *x*-axis shows 166 genetic instruments for mean spherical equivalent RE and their effect size estimates (betas) with RE. The *y*-axis shows the association of the same variants with cataract risk. The MR IVW regression line is plotted. The outlier genetic variant rs429358 detected by the MR-PRESSO model is labeled in *red*.

To investigate whether genetically determined POAG and myopic RE increase the risk of cataract, we conducted two-sample MR analyses. We found that genetically determined POAG was significantly associated with cataract risk (IVW model: OR = 1.04; 95% CI, 1.01–1.08; *P* = 0.018) (Table, Fig. 2, Supplementary Tables S12 and S22). We also found evidence for a causal effect of RE on cataract risk, as a more negative mean spherical equivalent RE was associated with an increased risk of cataract (IVW model: OR per diopter more hyperopic mean spherical equivalent = 0.92; 95% CI, 0.89–0.93; *P* = 6.51 × 10^−13^) (Table, Fig. 3, Supplementary Tables S13 and S23).

**Table.** MR Results of the Associations of Genetically Predicted Risk Factors With Cataract

Exposure (Source)	Outcome	Genetic Instruments, *n*	MR Method	OR (95% CI)	*P*	Detected Outlier SNV Via MR-PRESSO
POAG (IGGC)	Cataract (UKB)	46	IVW	1.04 (1.01–1.08)	**0.018**	—
POAG (IGGC)	Cataract (UKB)	44	MR-PRESSO model	1.03 (1.00–1.07)	**0.044**	rs6475604; rs33912345
Myopic RE (UKB)	Cataract surgery (GERA)	166	IVW	0.92 (0.89–0.93)	**6.51 × 10^−^^13^**	—
Myopic RE (UKB)	Cataract surgery (GERA)	165	MR-PRESSO model	0.92 (0.90–0.94)	**9.29 × 10^−^^12^**	rs429358
T2D (UKB)	Cataract surgery (GERA)	66	IVW	1.00 (0.96–1.04)	0.96	—
T2D (UKB)	Cataract surgery (GERA)	66	MR-PRESSO model	1.00 (0.96–1.04)	0.96	NA
SBP (GERA)	Cataract (UKB)	20	IVW	1.00 (0.99–1.01)	0.68	
SBP (GERA)	Cataract (UKB)	20	MR-PRESSO model	1.00 (0.99–1.01)	0.68	NA
DBP (GERA)	Cataract (UKB)	20	IVW	1.00 (0.99–1.02)	0.36	—
DBP (GERA)	Cataract (UKB)	20	MR-PRESSO model	1.01 (0.99–1.02)	0.37	NA
BMI (GIANT + GERA)	Cataract (UKB)	153	IVW	0.94 (0.87–1.03)	0.18	—
BMI (GIANT + GERA)	Cataract (UKB)	152	MR-PRESSO model	0.95 (0.88–1.03)	0.25	rs889398
Cigarette smoking initiation (GSCAN)	Cataract surgery (GERA)	231	IVW	1.15 (0.98–1.34)	0.08	—
Cigarette smoking initiation (GSCAN)	Cataract surgery (GERA)	231	MR-PRESSO model	1.15 (0.98–1.34)	0.08	NA
Cigarettes per day (GSCAN)	Cataract surgery (GERA)	47	IVW	1.06 (0.90–1.25)	0.51	—
Cigarettes per day (GSCAN)	Cataract surgery (GERA)	47	MR-PRESSO model	1.06 (0.90–1.25)	0.51	NA
Lifetime smoking (UKB)	Cataract surgery (GERA)	121	IVW	1.07 (0.83–1.37)	0.62	—
Lifetime smoking (UKB)	Cataract surgery (GERA)	121	MR-PRESSO model	1.07 (0.83–1.37)	0.62	NA
Alcohol drinks/week (GSCAN)	Cataract surgery (GERA)	93	IVW	0.86 (0.71–1.04)	0.12	—
Alcohol drinks/week (GSCAN)	Cataract surgery (GERA)	92	MR-PRESSO model	0.90 (0.74–1.08)	0.27	rs62641967

*Note:* In the current study, we retrieved genetic association data from the GWAS of cataract conducted in 28,092 cataract cases and 50,487 controls; all GERA participants were of European ancestry.^12^ However, to make sure that study samples for exposures did not overlap with those for the outcome, in some MR analyses (i.e., association of POAG, BP, or BMI with cataract risk), genetic association data for cataract risk were retrieved from our previous GWAS study conducted in the UK Biobank.^12^ *Abbreviations: n*, number of genetic instruments after clumping; MR, Mendelian randomization; OR, odds ratio; CI, confidence interval; SNV, single nucleotide variation; POAG, primary open-angle glaucoma; IGGC, International Glaucoma Genetics Consortium; UKB, UK Biobank; IVW, inverse-variance weighted; RE, refractive error; GERA, Genetic Epidemiology Research on Adult Health and Aging; T2D, type 2 diabetes; SBP, systolic blood pressure; DBP, diastolic blood pressure; GIANT, Genetic Investigation of Anthropomorphic Traits; GSCAN, GWAS and Sequencing Consortium of Alcohol and Nicotine use. Highlighted in bold *P*-values are significant (*P* < 0.05) *P*-values.

As significant heterogeneity amongst the effects of the genetic instruments was detected using the computed Cochran *Q* statistics (POAG: *Q* = 97.3, *P* = 1.03 × 10^−5^; myopic RE: *Q* = 214.98, *P* = 0.0054), we conducted a MR-PRESSO model to identify potential outliers for POAG and myopic RE. After excluding outlier genetic variants (i.e., SNVs rs6475604 at *CDKN2B-AS1* and rs33912345 at *SIX6* for POAG, and SNV rs429358 at *APOE* for myopic RE) as the source of most of the observed heterogeneity, genetically determined POAG and RE were confirmed to be associated with cataract risk (Table). MR-Steiger results showed that the selected SNVs were valid instruments for the exposures and that the causal estimates were oriented in the expected direction.

Because recent MR studies suggested that lower (more myopic) RE is associated with POAG risk,^37^^,^^58^ we also conducted a multivariable MR analysis to assess the association of POAG with cataract risk after adjusting for the effect of RE. The association of POAG with cataract risk was similar after the adjustment of RE (OR = 1.05; 95% CI, 1.00–1.10; *P* = 0.030).

### Association of Clinical Risk Factors With Cataract Risk

Genetically determined T2D was not associated with an increased risk of cataract (OR = 1.00; 95% CI, 0.96–1.04; *P* = 0.96) (Table, Supplementary Fig. S1, Supplementary Tables S14 and S24). Similarly, we observed no association between increased genetically determined BMI or systolic and diastolic blood pressures and the risk of cataract using two-sample MR analyses (Table, Supplementary Figs. S2–S4, Supplementary Tables S15–S17 and S25–S27).

### Association of Behavioral Risk Factors With Cataract Risk

We found no evidence that cigarette smoking traits (i.e., smoking initiation, amount smoked, and lifetime smoking) or alcohol consumption (i.e., drinks per week) had a causal association with the risk of cataract using two-sample MR analyses (Table, Supplementary Figs. S5–S8, Supplementary Tables S18–S21 and S28–S31).

## Discussion

This study leveraged a MR framework to investigate the associations of clinical and behavioral factors with the risk of cataract. We found genetic evidence for a potential causal effect of POAG on cataract risk. Consistent with previous observational studies, we also found genetic evidence for a potential causal association between myopic RE and cataract risk. Those associations were robust in sensitivity analyses that address significant heterogeneity among the effects of the genetic instruments. Further, although multiple tests were conducted, the causality of myopic RE on cataract would clearly stand multiple testing correction. In contrast, we found no evidence for association of genetically determined T2D, BP, and BMI with cataract risk. In addition, we did not find evidence for associations with genetic predilections for cigarette smoking or alcohol consumption on cataract risk.

Our results are consistent with previous observational studies that have consistently reported RE or myopia as a risk factor for cataract.^14^^,^^18^^,^^20^^,^^22^^,^^24^^,^^27^^,^^29^^,^^30^^,^^61^^,^^62^ The first indication was from a study that measured axial thickness of human lenses that suggested that individuals presenting with axial myopia may have a higher likelihood of cataract detected at earlier ages.^28^ Using MR methods, we found genetic evidence that myopic RE was associated with an increased risk of cataract. Although the mechanisms behind this association are not entirely clear, prior works, especially from animal models, have attempted to provide potential biological explanations.^63^^–^^66^ Lipid peroxidation is thought to play a key role in cataractogenesis, especially in retinal degenerative disease and myopia.^63^^–^^65^ In addition, oxidative damage to lens proteins has been shown to influence myopic cataractogenesis,^66^ and the pathways involved in oxidative stress response are associated with cataracts in high myopia.^67^^,^^68^ Recently, a study in mouse models reported the dysregulation of the MAF–transforming growth factor-β1–crystallin axis as an underlying mechanism leading to an increased in lens size in highly myopic eyes.^69^ Finally, a comparative analysis of microarray expression patterns between human lens epithelia from high myopic eyes with that of emmetropic (normal refractive eye) controls suggests alterations in mitogen-activated protein kinase (MAPK) signaling and calcium signaling pathways in abnormal lens growth.^70^ Thus, the pathways described above should be investigated further to advance knowledge on the mechanisms commonly relevant to cataract and myopia.

The current study also determined that genetically determined POAG increased the risk of cataract. These findings built on our previous genetic investigations that reported a significant positive genetic correlation between glaucoma and cataract risk.^12^ Further, oxidative stress and endoplasmic reticular stress have been implicated in both vision disorders.^71^ Future investigations are needed to understand the shared molecular biology pathways involved between POAG and cataract.

In this study, our MR estimates did not indicate a potential causal association for T2D and the risk of cataract. Our results are consistent with recent MR studies on the association between T2D and cataract risk.^42^^,^^43^ Although a prior MR study found no evidence of causal relationship between T2D and cataract risk in Europeans, a causal effect of T2D on the development of cataract was observed in East Asians.^42^ Moreover, a recent MR study found no evidence for a causal effect of T2D on cataract risk in the UK Biobank study but detected significant association between the two diseases in the FinnGen consortium.^43^ Further studies in larger samples of European and non-European populations are required to clarify the potential effect of T2D on cataract risk.

We also found no evidence of causal association between higher systolic or diastolic blood pressure and cataract risk. Although hypertension has been related to incident cataract in observational studies,^20^^–^^22^ a recent MR study found limited evidence for a causal effect of SBP on cataract risk.^43^ Similarly, in our current MR study, we found no evidence of a causal association between higher BMI and cataract risk. Although obesity (or higher BMI) has been reported to be associated with an increased risk of cataract surgery,^15^ recent MR studies on the association of obesity or BMI with cataract risk are conflicting.^40^^,^^43^ Although higher genetically determined BMI was associated with an increased risk of cataract in the FinnGen consortium,^43^ obesity was not causally associated with age-related cataract in the Blue Mountains Eye Study or in the UK Biobank study.^40^^,^^43^ Additional MR studies using many more genetic instruments and larger samples may elucidate the potential effect of BMI on cataract risk.

Many observational studies have consistently reported the association of cigarette smoking with cataract risk,^13^^–^^15^^,^^20^^,^^23^ suggesting that this lifestyle exposure might be a modifiable risk factors for cataract, and a causal effect of smoking initiation on cataract risk has been identified in recent MR studies.^41^^,^^43^ In contrast, our results provide no genetic evidence of a potential causal association of cigarette smoking with cataract risk, even if a large number of genetic instruments derived from the recent and large GSCAN study^52^ were used for some exposures (i.e., smoking initiation or amount smoked). Further investigations are required to understand the reasons for those inconsistent results.

The effect of alcohol consumption on cataract risk seem to differ according to the trait tested (i.e., drinker status, moderate vs. heavy alcohol consumption among drinkers, and type of alcohol beverage).^16^^,^^17^^,^^25^ In a meta-analysis study, Gong and colleagues^16^ reported that heavy alcohol consumption significantly increased the risk of cataract, whereas moderate consumption may be protective. Those findings were reinforced by a recent observational study that found a lower risk of undergoing cataract surgery with low to moderate alcohol consumption, especially wine consumption.^25^ More recently, a MR study found no evidence for a causal effect of alcohol consumption (drinks per week) on cataract risk in either the FinnGen consortium or the UK Biobank study.^43^ Similarly, although we used a large number of genetic instruments for this lifestyle exposure (drinks per week), our current MR estimates did not indicate a potential causal association for alcohol consumption with cataract risk.

Our study has important strengths. Our two-sample MR analyses were performed using independent and large GWAS summary statistics for both exposures and outcomes. This allowed us to evaluate the association of behavioral and clinical risk factors with the risk of cataract using strong genetic instruments that consist of multiple SNVs across the genome previously reported as genome-wide significant in large samples. We found similar results using different MR methods, especially MR-PRESSO, which is a robust method for sensitivity analysis that excludes outlier SNVs for which horizontal pleiotropic effect can be observed. Finally, for all of the MR analyses, the two samples tested represented the same ethnic group (i.e., European ancestry individuals), enabling comparisons between exposures and outcomes and ensuring the validity of causal estimates.

Our study also has limitations. First, although we used summary statistics from the largest GWAS of cataract conducted to date,^12^ the number of cataract cases (i.e., mainly patients who underwent cataract surgery) is limited compared to the number of cases used in MR studies for other outcomes. Future GWAS studies of cataract could focus on more statistically powerful cataract outcomes, such as age of cataract diagnosis or age at cataract surgery. Those outcomes could then be used in future MR studies that could provide additional important insights into the causal pathways underlying cataractogenesis. Second, subtypes of cataract were not available in the GERA and UK Biobank cohorts when we conducted our previous GWAS study.^12^ Future MR studies using other cohorts will determine whether genetically determined POAG and myopic RE are also associated with specific cataract subtypes (i.e., nuclear, cortical, or subcapsular) and the extent to which these potential specific causal associations display shared pathways across subtypes. Third, the generalizability of our findings might not be applicable to non-European ancestry populations, due to the previously observed (and above-mentioned) inconsistent associations between genetically determined T2D and cataract between East Asians and Europeans.^42^ Future investigations could address this limitation as genetic data in other ethnic groups become more available.

Our findings support the use of POAG and myopic RE as ophthalmic metrics to help stratify risk of cataract in the general population. Understanding which factors increase risk of cataract could not only help identify high-risk individuals but also help develop preventative strategies.

## Conclusions

Using a two-sample MR approach, our study supports previous observational studies showing that myopic RE is a risk factor for cataract. Our study also provides evidence that genetically determined POAG increased the risk of cataract. Altogether, clarifying the nature of the relationships between those ophthalmic conditions and cataract could open new avenues of investigation into the specific mechanisms underlying this leading cause of blindness.

## Supplementary Material

Supplement 1
