# Supplementary material for: Association of Behavioral and Clinical Risk Factors With Cataract: A Two-Sample Mendelian Randomization Study
Source: Invest Ophthalmol Vis Sci. 2023 Jul 17;64(10):19. doi: 10.1167/iovs.64.10.19 (PMC10362921; doi:10.1167/iovs.64.10.19)
Supplement: Supplement 1 [file iovs-64-10-19_s001.pdf]

Supplementary Material

Jiang, et al. Association of Behavioral and Clinical Risk Factors with Cataract: A Two-Sample Mendelian Randomization Study

Supplementary Figure S1. Association of T2D-associated variants with the risk of cataract.

Supplementary Figure S2. Association of SBP-associated variants with the risk of cataract.

Supplementary Figure S3. Association of DBP-associated variants with the risk of cataract.

Supplementary Figure S4. Association of BMI-associated variants with the risk of cataract.

Supplementary Figure S5. Association of cigarette smoking initiation-associated variants with the risk of cataract.

Supplementary Figure S6. Association of cigarettes per day-associated variants with the risk of cataract.

Supplementary Figure S7. Association of lifetime smoking-associated variants with the risk of cataract.

Supplementary Figure S8. Association of alcohol consumption-associated variants with the risk of cataract.

Supplementary Table S1. Description of the samples used for exposures and outcome.

Supplementary Table S2. Genetic instruments (N=46) for POAG.

Supplementary Table S3. Genetic instruments (N=166) for mean spherical equivalent RE.

Supplementary Table S4. Genetic instruments (N=66) for T2D.

Supplementary Table S5. Genetic instruments (N=20) for SBP.

Supplementary Table S6. Genetic instruments (N=20) for DBP.

Supplementary Table S7. Genetic instruments (N=153) for BMI.

Supplementary Table S8. Genetic instruments (N=231) for cigarette smoking initiation.

Supplementary Table S9. Genetic instruments (N=47) for cigarettes per day.

Supplementary Table S10. Genetic instruments (N=121) for lifetime smoking.

Supplementary Table S11. Genetic instruments (N=93) for alcohol consumption (drinks per week).

Supplementary Table S12. Mendelian randomization leave-one-out analysis for POAG suggesting evidence of causality with cataract.

Supplementary Table S13. Mendelian randomization leave-one-out analysis for mean spherical equivalent RE suggesting evidence of causality with cataract.

Supplementary Table S14. Mendelian randomization leave-one-out analysis for T2D suggesting a lack of association with cataract.

Supplementary Table S15. Mendelian randomization leave-one-out analysis for SBP showing a lack of association with cataract.

Supplementary Table S16. Mendelian randomization leave-one-out analysis for DBP showing a lack of association with cataract.

Supplementary Table S17. Mendelian randomization leave-one-out analysis for BMI showing a lack of association with cataract.

Supplementary Table S18. Mendelian randomization leave-one-out analysis for cigarette smoking initiation showing a lack of association with cataract.

Supplementary Table S19. Mendelian randomization leave-one-out analysis for cigarettes per week showing a lack of association with cataract.

Supplementary Table S20. Mendelian randomization leave-one-out analysis for lifetime smoking showing a lack of association with cataract.

Supplementary Table S21. Mendelian randomization leave-one-out analysis for alcohol consumption showing a lack of association with cataract.

Supplementary Table S22. Evaluation of MR associations of POAG with cataract under different MR models

Supplementary Table S23. Evaluation of MR associations of mean spherical equivalent RE with cataract under different MR models

Supplementary Table S24. Evaluation of MR associations of T2D with cataract under different MR models

Supplementary Table S25. Evaluation of MR associations of SBP with cataract under different MR models

Supplementary Table S26. Evaluation of MR associations of DBP with cataract under different MR models

Supplementary Table S27. Evaluation of MR associations of BMI with cataract under different MR models

Supplementary Table S28. Evaluation of MR associations of smoking initiation with cataract under different MR models

Supplementary Table S29. Evaluation of MR associations of cigarettes per day with cataract under different MR models

Supplementary Table S30. Evaluation of MR associations of lifetime smoking with cataract under different MR models

Supplementary Table S31. Evaluation of MR associations of alcohol drinks per week with cataract under different MR models

Supplementary Figure S1. Association of T2D-associated variants with the risk of cataract. The x-axis shows 66 genetic instruments for T2D and their effect size estimates (ORs) with T2D. The y-axis shows the association of the same variants with cataract risk. The Mendelian Randomization (MR) inverse-weighted (IVW) regression line is plotted.

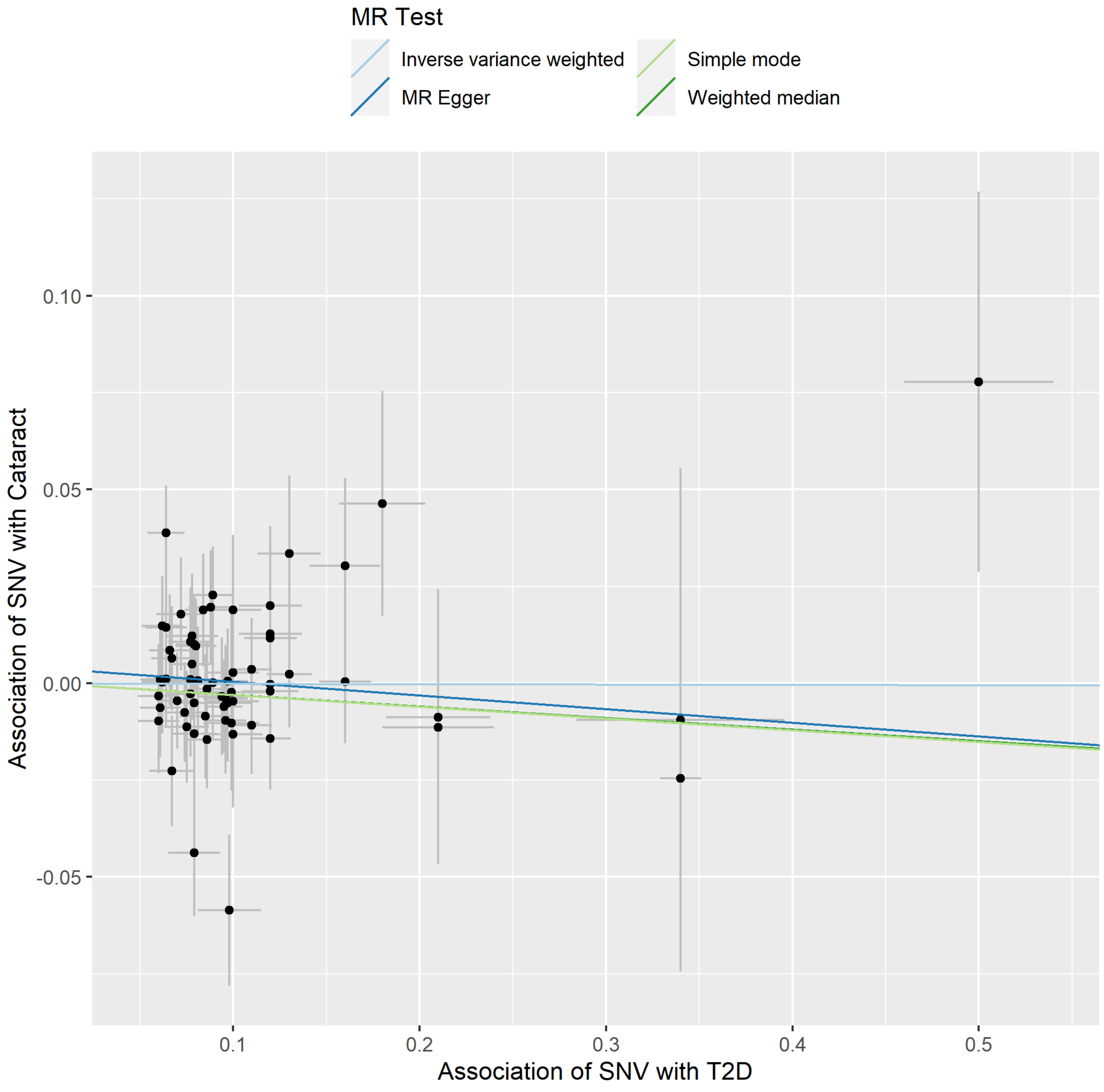

Supplementary Figure S2. Association of SBP-associated variants with the risk of cataract. The x-axis shows 20 genetic instruments for SBP and their effect size estimates (betas) with SBP. The y-axis shows the association of the same variants with cataract risk. The Mendelian Randomization (MR) inverse-weighted (IVW) regression line is plotted.

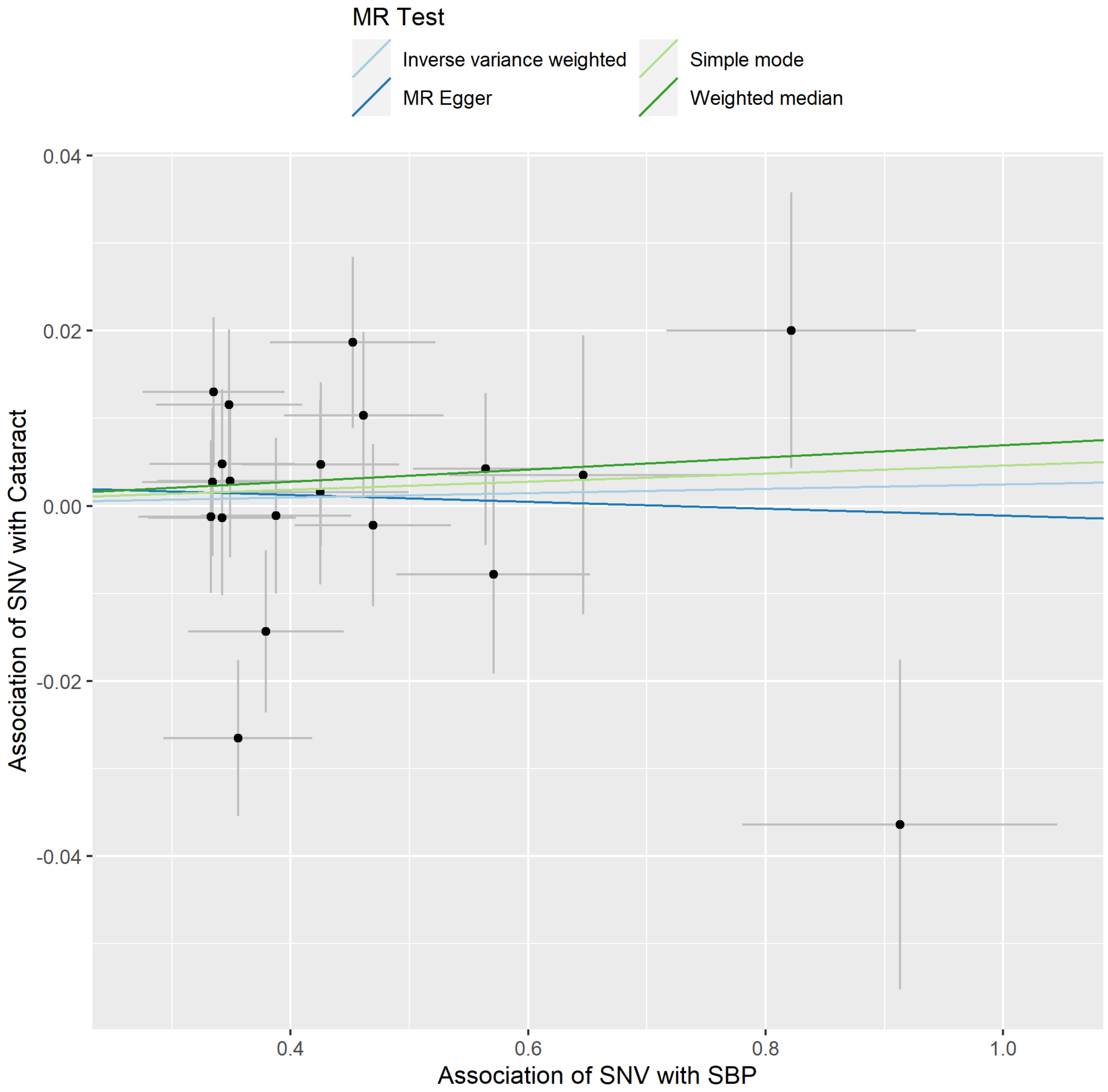

Supplementary Figure S3. Association of DBP-associated variants with the risk of cataract. The x-axis shows 20 genetic instruments for DBP and their effect size estimates (betas) with DBP. The y-axis shows the association of the same variants with cataract risk. The Mendelian Randomization (MR) inverse-weighted (IVW) regression line is plotted.

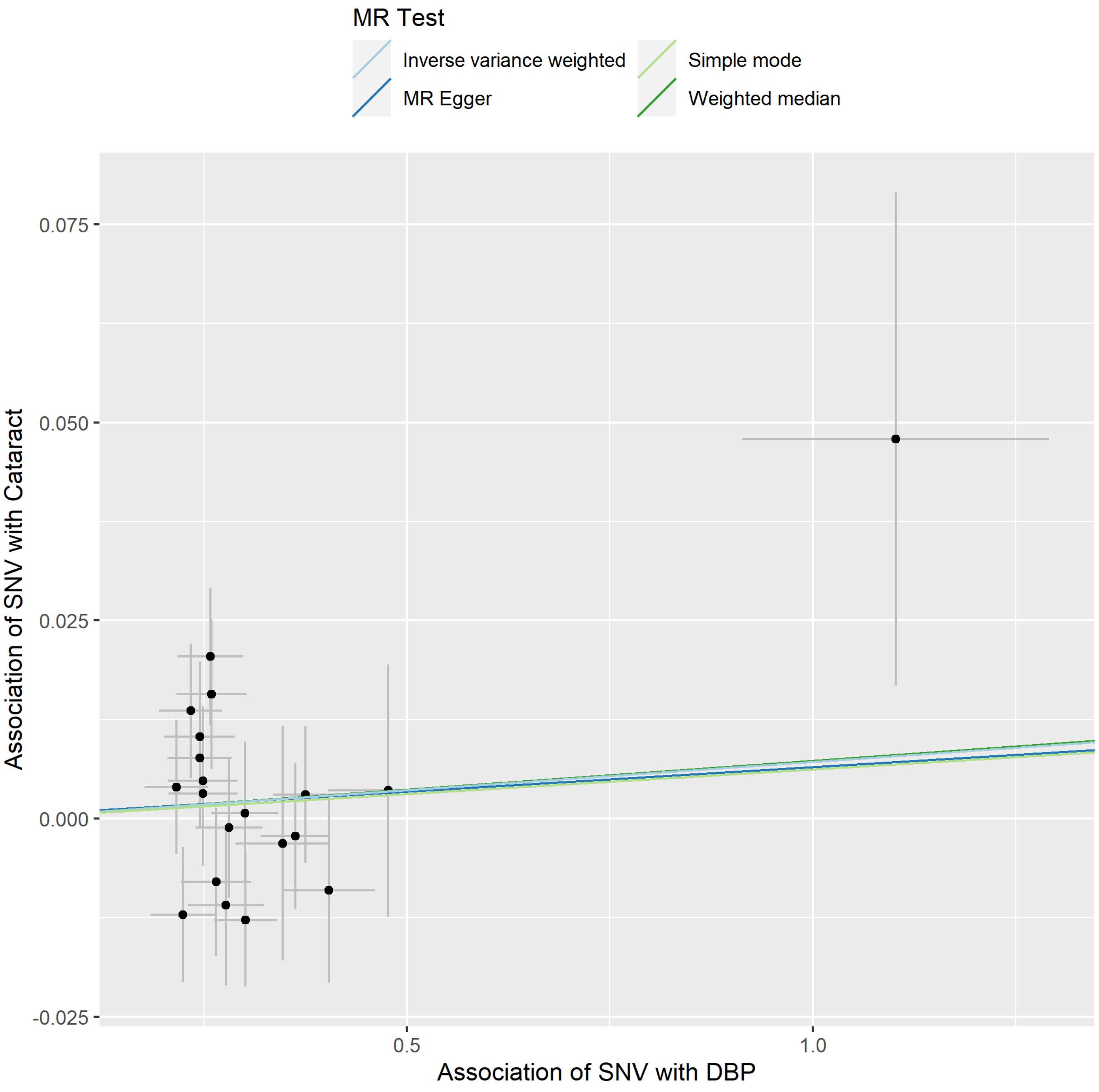

Supplementary Figure S4. Association of BMI-associated variants with the risk of cataract. The x-axis shows 153 genetic instruments for BMI and their effect size estimates (betas) with BMI. The y-axis shows the association of the same variants with cataract risk. The Mendelian Randomization (MR) inverse-weighted (IVW) regression line is plotted. The outlier genetic variant rs889398 detected by the MR-PRESSO model is labeled in red.

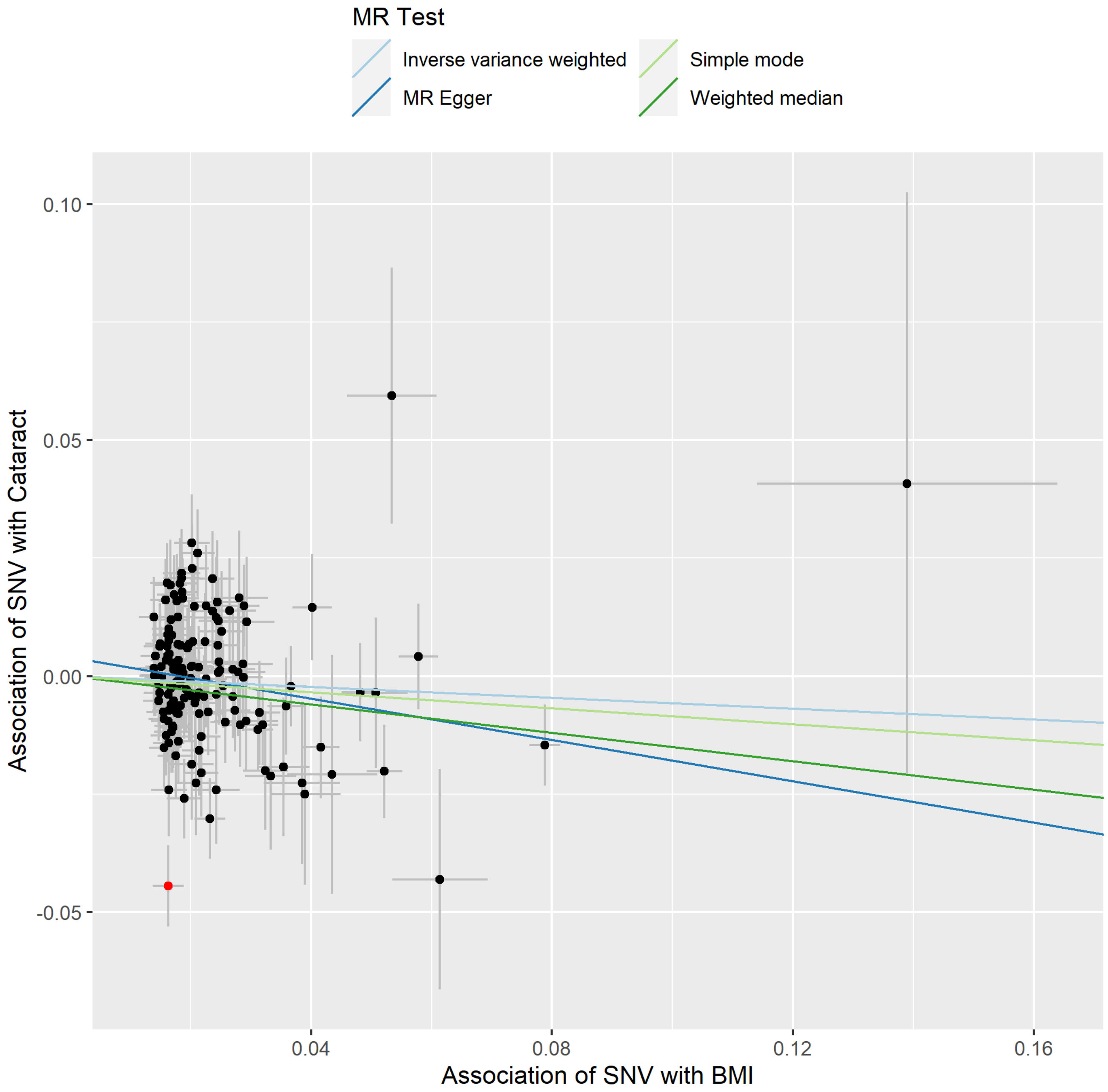

Supplementary Figure S5. Association of cigarette smoking initiation-associated variants with the risk of cataract. The x-axis shows 231 genetic instruments for cigarette smoking initiation and their effect size estimates (ORs) with cigarette smoking initiation. The y-axis shows the association of the same variants with cataract risk. The Mendelian Randomization (MR) inverse-weighted (IVW) regression line is plotted.

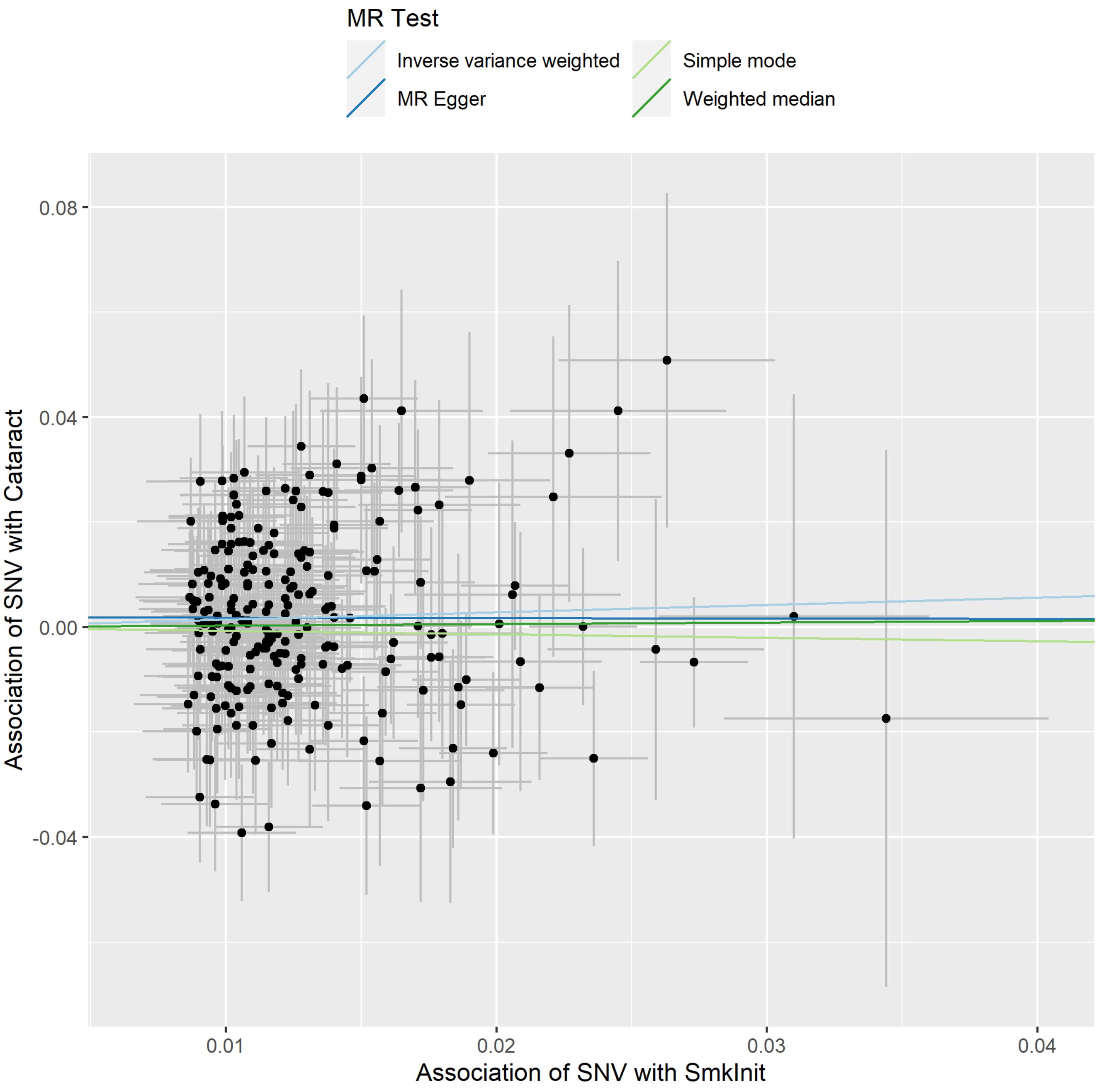

Supplementary Figure S6. Association of cigarettes per day-associated variants with the risk of cataract. The x-axis shows 47 genetic instruments for the amount smoked and their effect size estimates (betas) with the number of cigarettes smoked per day. The y-axis shows the association of the same variants with cataract risk. The Mendelian Randomization (MR) inverse-weighted (IVW) regression line is plotted.

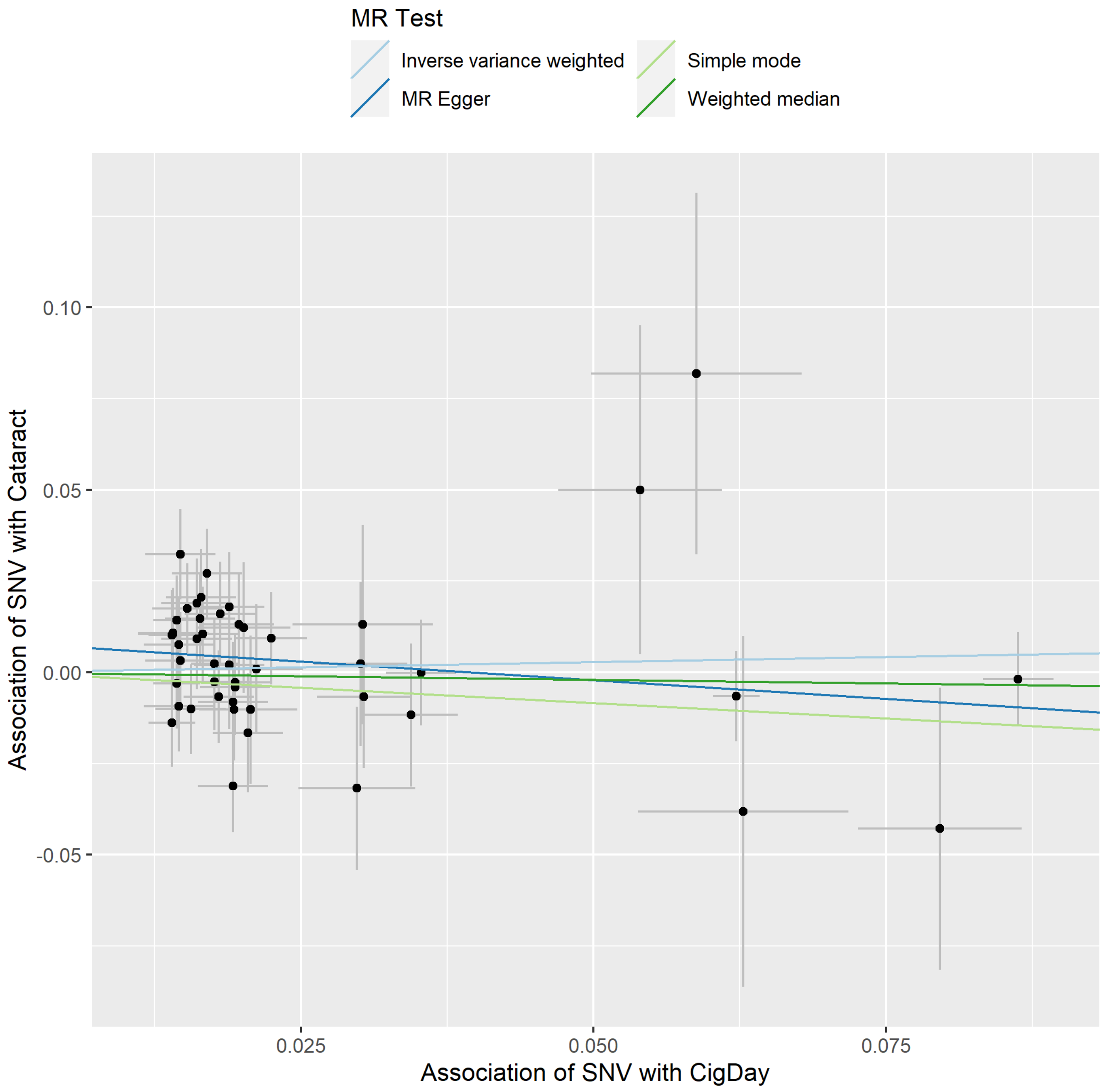

Supplementary Figure S7. Association of lifetime smoking-associated variants with the risk of cataract. The x-axis shows 121 genetic instruments for lifetime smoking and their effect size estimates (betas) with lifetime smoking. The y-axis shows the association of the same variants with cataract risk. The Mendelian Randomization (MR) inverse-weighted (IVW) regression line is plotted.

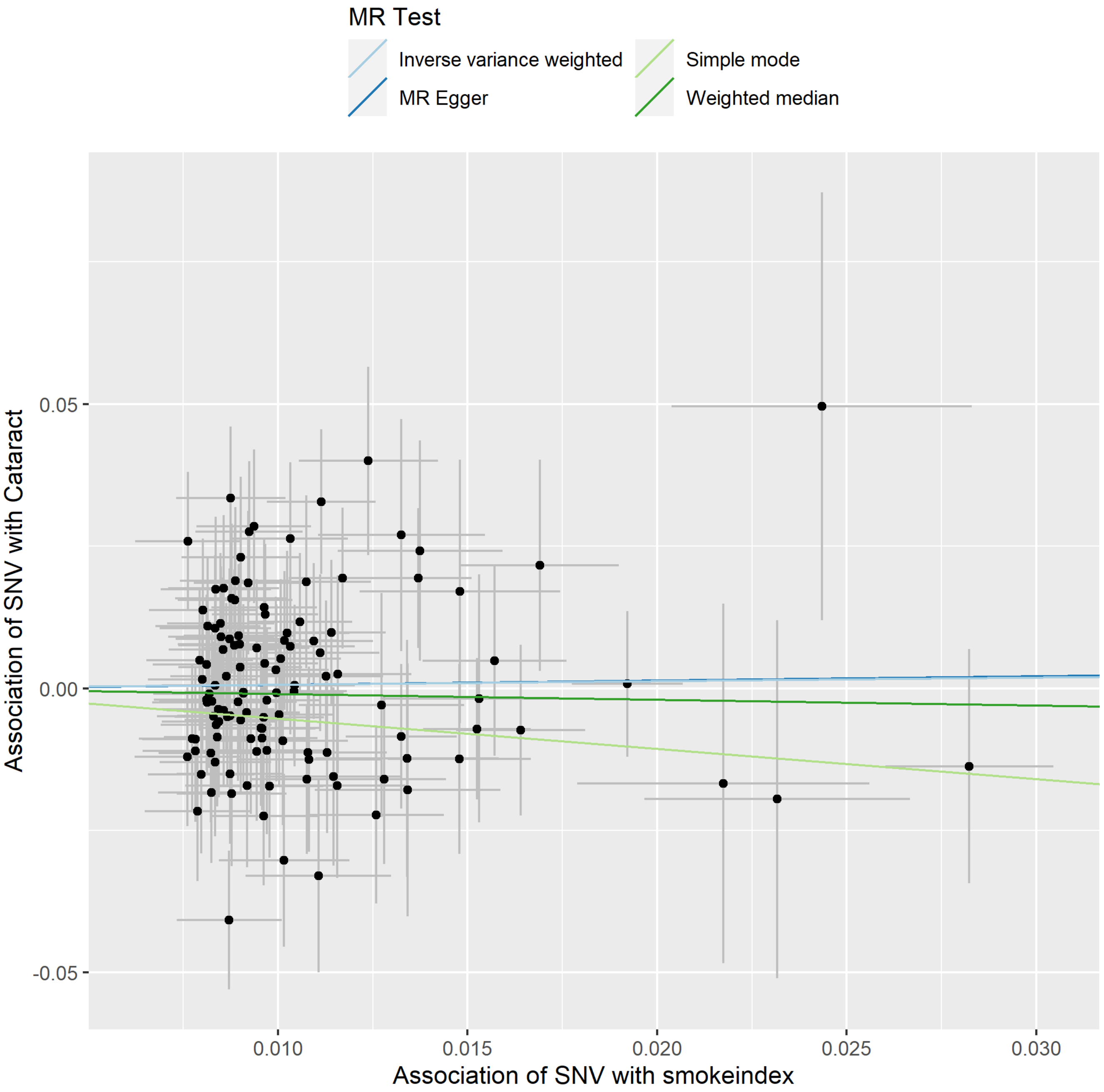

Supplementary Figure S8. Association of alcohol consumption-associated variants with the risk of cataract. The x-axis shows 93 genetic instruments for alcohol consumption and their effect size estimates (ORs) with alcohol consumption (drinks per week). The y-axis shows the association of the same variants with cataract risk. The Mendelian Randomization (MR) inverse-weighted (IVW) regression line is plotted. The outlier genetic variant rs62641967 detected by the MR-PRESSO model is labeled in red.

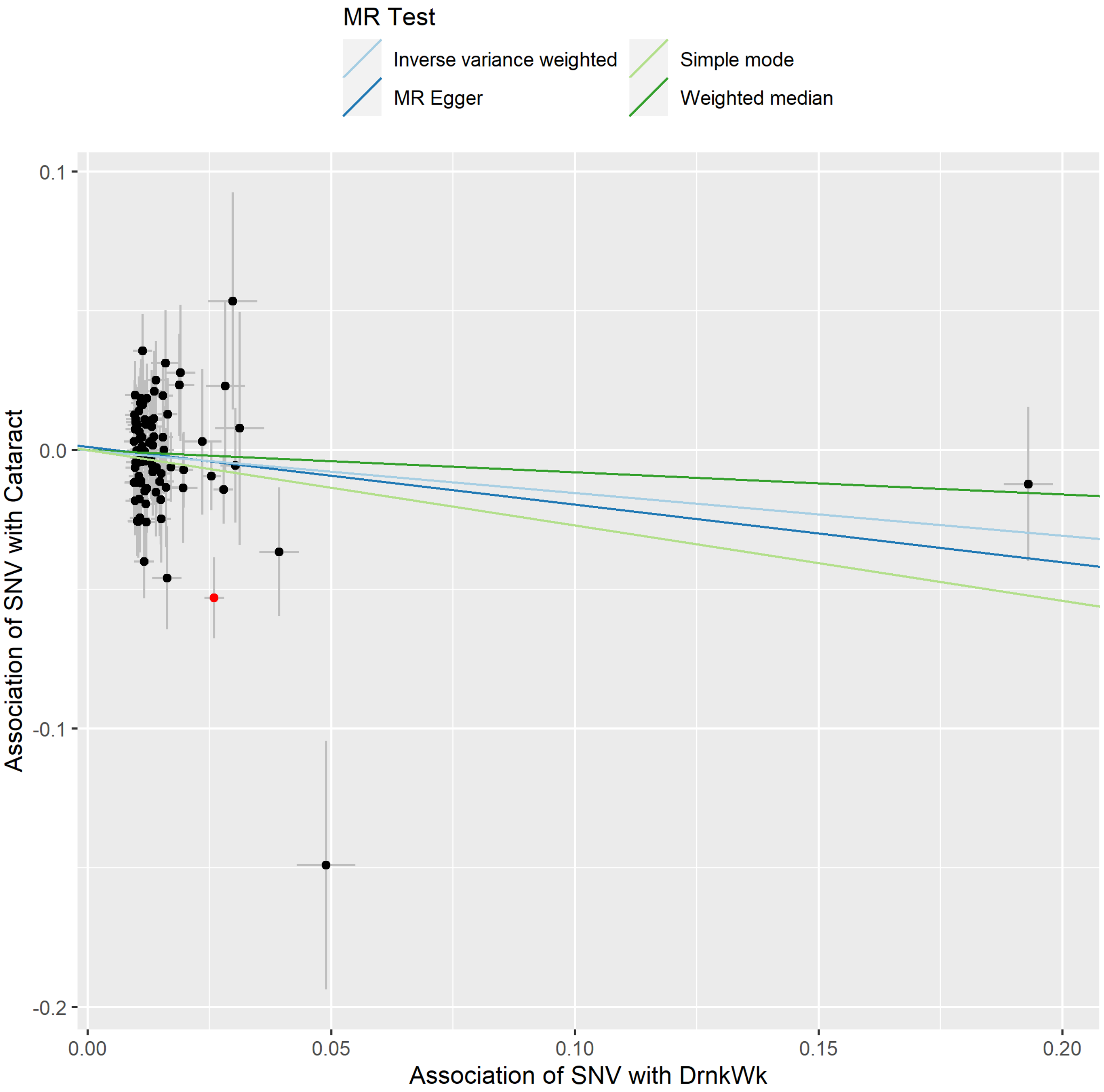

Supplementary Table S1. Description of the samples used for exposures and outcome.

EXPOSURE				OUTCOME	
**Risk factor**	**Source Ref (PMID #)**	**No. of participants (All of European ancestry)**	**N Genetic Instruments**	**Trait**	**Source Ref (PMID #)**
POAG	IGGC PMID: 33627673	15,229 cases and 177,473 controls	46	Cataract (self-reported cataract operation or/and diagnosis)	UKB European sample 31,852 cases and 428,084 controls PMID: 34127677
Blood pressure (SBP and DBP)	GERA PMID: 27841878	80,792 individuals	20 for SBP 20 for DBP		
BMI	GERA and GIANT PMID: 30108127	234,069 GIANT individuals and 81,278 GERA individuals	153		
Myopic refractive error	UKB PMID: 32231278	102,117 individuals	166	Cataract (surgery)	GERA non-Hispanic whites 28,092 cases and 50,487 controls PMID: 34127677
Type 2 diabetes	UKB PMID: 30297969	19,119 cases and 423,698 controls	66		
Cigarette smoking initiation	GSCAN PMID: 36477530	805,431 individuals	231		
Cigarettes per day	GSCAN PMID: 36477530	326,497 individuals	47		
Life time smoking	UKB PMID: 31689377	462,690 individuals	121		
Alcohol consumption (drinks per week)	GSCAN PMID: 36477530	619,011 individuals	93		

Note: In the current study, we retrieved genetic association data from the GWAS of cataract conducted in 28,092 cataract cases and 50,487 controls; all GERA participants of European ancestry (Choquet H, et al. Nat Commun. Jun 14 2021;12(1):3595). However, to make sure that study samples for exposures did not overlap with those for the outcome, in some MR analyses (i.e., association of POAG, BP, or BMI with cataract risk), genetic association data for cataract risk were retrieved from our previous GWAS study conducted in the UK Biobank (UKB) (Choquet H, et al. Nat Commun. Jun 14 2021;12(1):3595).

Supplementary Table S2. Genetic instruments (46-SNP - $P<5.0x10^{-8}$) for POAG**.** Abbreviations: SNP, single nucleotide polymorphism; EA, effect allele; OA, other allele; POAG, primary open-angle glaucoma; se, standard error.

SNP	EA	OA	beta. POAG	se. POAG	pval. POAG	beta. Cataract	se. Cataract	pval. Cataract
rs10248136	T	C	0.088	0.014	1.30E-10	0.003	0.008	0.692
rs10448285	T	C	-0.119	0.015	9.90E-16	-0.007	0.009	0.422
rs10517281	A	G	0.099	0.017	7.65E-09	-0.001	0.011	0.955
rs10800155	A	G	0.291	0.019	7.56E-52	0.030	0.013	0.018
rs109467	T	C	-0.083	0.014	4.05E-09	0.007	0.009	0.451
rs1139795	T	C	-0.143	0.021	7.03E-12	0.015	0.012	0.200
rs113985657	T	C	0.108	0.019	5.54E-09	-0.020	0.012	0.090
rs114367221	T	C	0.148	0.026	1.33E-08	0.001	0.015	0.953
rs12208086	A	G	0.108	0.016	1.62E-11	0.000	0.010	0.970
rs12540035	A	G	0.109	0.015	9.40E-13	0.005	0.009	0.620
rs1577488	A	G	-0.130	0.020	1.45E-10	-0.011	0.013	0.426
rs1649068	A	C	0.106	0.014	9.00E-15	0.020	0.008	0.018
rs17527016	T	C	-0.112	0.017	4.15E-11	0.018	0.010	0.059
rs2027312	A	G	-0.083	0.014	1.67E-09	-0.010	0.008	0.237
rs2278565	C	G	0.102	0.019	3.31E-08	-0.010	0.011	0.390
rs2472494	T	C	0.189	0.014	4.76E-43	0.025	0.009	0.003
rs2514879	T	G	-0.125	0.022	7.81E-09	-0.009	0.013	0.503
rs257336	T	G	-0.086	0.016	3.18E-08	0.016	0.009	0.070
rs2579998	T	C	0.209	0.023	1.47E-20	-0.004	0.014	0.783
rs2667477	T	C	-0.109	0.014	2.84E-15	0.020	0.008	0.018
rs2735114	A	G	0.080	0.014	1.80E-08	-0.009	0.009	0.274
rs28497695	A	G	-0.109	0.017	1.39E-10	-0.025	0.011	0.018
rs33912345	A	C	-0.178	0.014	2.43E-37	0.025	0.009	0.003
rs3825942	A	G	-0.124	0.019	7.48E-11	-0.007	0.012	0.555
rs41543317	A	G	-0.084	0.014	4.67E-09	0.001	0.009	0.911
rs4577906	C	G	-0.076	0.014	3.00E-08	0.009	0.009	0.292
rs4612174	T	G	0.116	0.014	3.39E-16	0.006	0.009	0.513
rs4653159	A	G	0.109	0.019	4.19E-09	0.011	0.011	0.340
rs4775427	T	C	0.075	0.014	4.18E-08	-0.007	0.008	0.384
rs55882252	T	C	-0.099	0.014	6.65E-13	-0.003	0.008	0.701
rs56233426	A	G	0.104	0.014	3.66E-14	0.009	0.009	0.275
rs58073046	A	G	-0.165	0.021	9.21E-16	-0.013	0.013	0.316
rs6117318	A	G	-0.089	0.015	3.54E-09	-0.001	0.009	0.948
rs62283811	T	C	0.172	0.029	4.45E-09	-0.002	0.019	0.925
rs6462143	C	G	0.103	0.018	1.83E-08	-0.005	0.009	0.604
rs6475604	T	C	-0.265	0.014	5.17E-80	-0.044	0.009	0.000
rs6490697	T	G	0.120	0.018	1.20E-11	-0.022	0.012	0.062
rs6602453	A	C	0.080	0.014	2.25E-08	0.009	0.009	0.303
rs6845653	T	C	-0.174	0.014	1.44E-34	-0.015	0.009	0.095
rs686768	A	G	-0.150	0.024	3.77E-10	-0.036	0.015	0.019
rs7137828	T	C	0.096	0.015	7.65E-11	0.012	0.008	0.143
rs7739648	A	G	-0.125	0.014	5.04E-18	-0.001	0.009	0.902
rs9544017	A	G	-0.081	0.014	2.38E-09	-0.006	0.008	0.487

rs9819278	A	G	-0.091	0.014	5.03E-11	-0.013	0.009	0.134
rs9913911	A	G	0.154	0.014	2.40E-27	0.005	0.009	0.575
rs993471	A	G	-0.085	0.014	8.60E-10	0.009	0.009	0.320

Supplementary Table S3. Genetic instruments (166-SNP - P<5.0x10^{-8}) for mean spherical equivalent RE. Abbreviations: SNP, single nucleotide polymorphism; EA, effect allele; OA, other allele; RE, refractive error; se, standard error.

SNP	EA	OA	beta. RE	se. RE	pval. RE	beta. Cataract	se. Cataract	pval. Cataract
rs1002191	G	C	0.095	0.012	2.00E-17	0.002	0.013	0.902
rs10025605	T	A	0.093	0.013	6.30E-14	0.005	0.014	0.733
rs10204657	T	A	0.063	0.011	1.80E-08	0.010	0.013	0.407
rs10220706	A	C	0.087	0.012	8.10E-14	-0.012	0.013	0.343
rs1055356	T	C	0.081	0.011	1.30E-11	0.001	0.012	0.959
rs10824515	G	C	0.134	0.012	7.60E-32	0.010	0.013	0.430
rs10839545	G	A	-0.063	0.011	2.10E-08	-0.008	0.013	0.558
rs10853030	A	G	0.109	0.017	1.40E-10	-0.017	0.020	0.371
rs10890821	A	T	-0.076	0.011	6.10E-12	-0.009	0.012	0.465
rs10917958	C	T	0.114	0.013	4.90E-19	-0.025	0.015	0.084
rs1106107	G	A	-0.144	0.016	1.40E-19	0.019	0.017	0.256
rs11078724	G	A	0.056	0.011	4.20E-08	-0.004	0.012	0.770
rs11078918	G	C	-0.073	0.013	2.50E-09	0.018	0.014	0.223
rs11083244	T	C	0.070	0.013	6.10E-09	-0.007	0.014	0.602
rs11127261	G	A	0.090	0.012	4.00E-13	-0.042	0.013	0.002
rs11130365	C	T	-0.062	0.012	1.40E-08	0.003	0.013	0.805
rs11145746	G	A	0.093	0.014	4.70E-11	-0.005	0.015	0.744
rs1118543	G	C	-0.094	0.012	2.20E-15	0.021	0.013	0.107
rs11186433	G	A	-0.064	0.013	4.10E-08	-0.004	0.014	0.789
rs11602008	A	T	0.232	0.015	2.50E-56	-0.021	0.016	0.186
rs11614577	C	T	0.076	0.012	2.10E-11	-0.006	0.013	0.647
rs11751433	G	A	0.063	0.011	1.30E-08	0.014	0.012	0.261
rs11909473	A	G	0.074	0.012	1.50E-09	-0.011	0.013	0.406
rs12186577	T	C	0.126	0.019	1.20E-11	0.006	0.020	0.769
rs12193446	A	G	-0.447	0.019	2.10E-131	0.024	0.021	0.242
rs12308700	A	G	-0.067	0.011	1.20E-09	-0.001	0.012	0.964
rs12440787	G	A	0.076	0.014	3.80E-08	-0.027	0.015	0.071
rs12511880	T	A	-0.086	0.012	5.80E-14	0.000	0.013	0.991
rs1254701	A	G	-0.088	0.015	2.10E-09	0.003	0.017	0.849
rs12568187	T	C	0.080	0.011	6.70E-13	-0.003	0.012	0.823
rs12599282	T	C	-0.081	0.012	7.50E-12	-0.020	0.014	0.131
rs12615720	T	C	0.129	0.011	2.30E-31	-0.030	0.012	0.015
rs12853508	G	T	0.088	0.013	1.20E-11	0.008	0.014	0.585
rs13107325	C	T	-0.118	0.021	1.70E-08	-0.037	0.023	0.114
rs1323971	G	A	0.073	0.012	3.00E-10	-0.007	0.013	0.612
rs13280928	T	C	-0.090	0.017	3.30E-08	-0.029	0.019	0.129
rs1340044	T	A	0.101	0.011	4.70E-20	-0.012	0.012	0.313
rs1367023	G	A	0.100	0.013	2.60E-17	-0.020	0.014	0.151
rs1369821	T	C	-0.080	0.014	8.40E-09	0.022	0.016	0.161
rs1408345	G	A	-0.125	0.021	1.30E-09	0.002	0.023	0.936
rs1420667	T	C	-0.081	0.014	7.60E-09	-0.015	0.015	0.304
rs1427750	G	A	0.084	0.012	7.80E-12	-0.027	0.013	0.041
rs147268199	G	A	0.332	0.053	7.70E-11	0.013	0.060	0.828

rs147792504	T	A	0.249	0.040	3.20E-10	-0.043	0.047	0.358
rs1483208	G	A	0.071	0.012	4.30E-09	0.005	0.013	0.707
rs1487441	G	A	0.079	0.011	4.80E-13	-0.020	0.012	0.096
rs149850621	G	A	0.100	0.011	5.60E-19	-0.022	0.013	0.078
rs1550094	G	A	-0.205	0.012	2.60E-67	0.009	0.015	0.576
rs1550870	C	T	-0.073	0.011	1.40E-10	0.003	0.013	0.832
rs1571590	A	G	0.084	0.014	2.20E-09	0.006	0.015	0.714
rs1659563	A	G	0.065	0.011	4.30E-09	-0.004	0.013	0.776
rs17048756	C	T	0.097	0.012	8.10E-16	0.001	0.013	0.965
rs17283419	T	C	0.069	0.011	3.10E-09	-0.014	0.012	0.268
rs17497118	G	A	-0.085	0.014	2.10E-09	0.018	0.015	0.249
rs17499741	G	A	0.117	0.011	2.50E-25	-0.018	0.012	0.142
rs17855844	G	C	0.075	0.014	3.90E-08	-0.018	0.016	0.255
rs1858001	G	C	-0.108	0.012	2.30E-21	0.012	0.013	0.350
rs196051	A	G	-0.111	0.012	1.60E-22	-0.005	0.012	0.703
rs1963456	T	C	0.122	0.012	1.60E-24	-0.010	0.013	0.416
rs198442	C	T	0.081	0.011	2.70E-13	-0.005	0.014	0.726
rs201224	T	C	0.074	0.012	3.70E-10	0.005	0.013	0.665
rs2046411	C	T	0.068	0.012	1.60E-08	0.018	0.013	0.155
rs2137277	A	G	-0.137	0.014	1.30E-22	-0.007	0.015	0.634
rs2155413	C	A	0.095	0.011	1.80E-18	-0.023	0.012	0.063
rs2160729	C	T	-0.087	0.012	2.90E-12	0.017	0.013	0.192
rs2229741	C	T	0.087	0.011	8.90E-15	-0.013	0.012	0.300
rs2278161	T	C	-0.080	0.013	4.00E-09	0.001	0.015	0.965
rs2281827	C	T	-0.099	0.013	2.20E-14	-0.030	0.014	0.037
rs2326838	G	A	0.080	0.012	4.30E-11	0.000	0.013	0.974
rs2329144	G	A	-0.069	0.013	3.90E-08	0.025	0.014	0.066
rs2745939	C	A	-0.063	0.012	2.50E-08	0.018	0.013	0.163
rs2808510	C	T	0.099	0.011	2.90E-19	0.008	0.013	0.511
rs2855530	G	C	0.119	0.011	6.60E-28	-0.038	0.013	0.003
rs2856250	A	G	-0.072	0.012	6.40E-10	0.018	0.013	0.165
rs2908972	T	A	0.145	0.012	1.30E-36	-0.006	0.013	0.644
rs2965185	T	C	0.077	0.012	2.90E-10	-0.012	0.013	0.365
rs297588	A	G	-0.080	0.012	2.20E-11	0.031	0.014	0.021
rs3138142	C	T	-0.226	0.013	1.70E-70	0.021	0.015	0.172
rs34695788	A	G	0.078	0.011	1.40E-12	-0.014	0.012	0.261
rs35033460	A	T	0.076	0.014	3.60E-08	-0.015	0.017	0.357
rs36024104	A	G	0.109	0.014	5.50E-15	-0.020	0.016	0.210
rs36212732	A	G	0.098	0.014	1.90E-12	-0.028	0.015	0.055
rs3778987	C	T	-0.065	0.012	2.70E-09	0.008	0.015	0.606
rs3812112	T	A	0.089	0.012	4.50E-15	0.004	0.013	0.748
rs3819878	T	C	0.075	0.013	1.60E-08	0.001	0.014	0.955
rs4262652	G	A	-0.072	0.012	4.30E-10	0.006	0.013	0.627
rs4278108	G	A	-0.074	0.013	1.10E-08	0.035	0.015	0.016
rs429358	T	C	-0.096	0.016	2.00E-09	0.100	0.019	1.39E-07
rs442439	G	A	0.078	0.012	2.50E-11	-0.018	0.013	0.165
rs4517452	T	C	0.140	0.012	4.20E-33	-0.019	0.013	0.144
rs4766878	C	T	0.068	0.011	5.30E-10	-0.012	0.012	0.327

rs4792448	A	G	0.075	0.012	7.70E-09	-0.001	0.013	0.934
rs4793501	C	T	0.081	0.011	1.30E-12	-0.018	0.012	0.139
rs4856278	A	G	0.074	0.013	4.50E-08	-0.008	0.015	0.585
rs4858582	G	A	-0.082	0.011	3.40E-13	-0.007	0.012	0.559
rs500359	G	A	-0.071	0.011	8.30E-10	-0.006	0.012	0.634
rs501057	A	G	-0.059	0.011	8.10E-09	0.022	0.012	0.067
rs524952	T	A	0.251	0.011	5.20E-116	-0.004	0.012	0.749
rs55728756	T	C	-0.065	0.012	2.70E-08	-0.011	0.012	0.394
rs55754534	G	C	0.142	0.016	3.70E-21	-0.006	0.018	0.728
rs55899248	A	G	0.117	0.014	3.40E-18	-0.026	0.015	0.075
rs55978930	A	G	-0.126	0.012	1.50E-25	0.015	0.013	0.258
rs56158821	T	C	0.064	0.011	6.40E-09	-0.004	0.013	0.772
rs579728	G	A	0.084	0.011	1.90E-14	-0.011	0.012	0.356
rs6054512	C	T	-0.078	0.012	2.70E-12	-0.008	0.013	0.503
rs6059635	A	T	0.081	0.012	2.90E-11	-0.037	0.013	0.005
rs60743220	T	C	0.089	0.015	1.80E-10	-0.006	0.017	0.724
rs6121073	G	A	0.085	0.015	9.70E-09	-0.024	0.016	0.141
rs61421564	T	C	0.093	0.016	3.70E-09	-0.014	0.017	0.418
rs62067167	C	T	0.135	0.015	3.90E-20	-0.014	0.016	0.388
rs62169542	T	C	-0.106	0.011	5.10E-22	0.011	0.012	0.385
rs62182437	T	C	-0.083	0.013	1.90E-10	0.001	0.014	0.934
rs6421566	G	A	0.084	0.011	2.40E-14	0.001	0.012	0.968
rs654169	G	A	0.069	0.013	8.40E-09	-0.016	0.014	0.247
rs6548183	C	T	0.079	0.014	3.60E-08	-0.012	0.016	0.454
rs6680922	T	C	-0.115	0.012	2.00E-23	0.016	0.013	0.224
rs67362351	C	A	0.083	0.012	1.10E-12	-0.029	0.013	0.022
rs6764769	A	G	-0.102	0.011	4.40E-20	0.014	0.012	0.248
rs67745574	C	T	-0.076	0.013	6.10E-09	0.027	0.015	0.065
rs6967089	A	G	-0.120	0.015	1.90E-16	-0.025	0.016	0.128
rs6979354	C	T	0.068	0.011	6.20E-09	0.007	0.012	0.571
rs6980853	C	T	0.092	0.012	3.40E-15	0.003	0.013	0.836
rs6989160	A	C	0.070	0.012	5.90E-10	-0.018	0.013	0.156
rs7042950	A	G	0.119	0.014	3.50E-20	-0.029	0.015	0.043
rs7140259	T	G	-0.083	0.011	4.30E-14	0.017	0.012	0.166
rs7149665	C	T	-0.088	0.016	9.40E-09	0.025	0.017	0.131
rs7162310	C	T	-0.111	0.014	1.60E-17	0.020	0.015	0.171
rs7188859	T	C	0.182	0.012	3.10E-58	-0.043	0.013	0.001
rs72621438	C	G	-0.174	0.012	6.00E-52	0.031	0.013	0.015
rs72683465	T	G	0.101	0.013	8.50E-14	-0.016	0.015	0.282
rs72772496	T	C	0.114	0.021	4.40E-08	-0.029	0.023	0.217
rs72903976	C	T	-0.118	0.021	3.30E-09	-0.027	0.022	0.221
rs7326825	G	A	0.087	0.012	4.00E-13	-0.004	0.013	0.777
rs73294447	T	C	-0.332	0.048	8.40E-14	-0.075	0.049	0.127
rs7444298	A	G	0.073	0.013	6.00E-09	-0.016	0.014	0.242
rs745030	G	A	0.155	0.011	1.60E-42	-0.006	0.012	0.612
rs7465621	G	A	-0.106	0.018	1.80E-10	0.022	0.019	0.234
rs75012440	G	A	0.092	0.016	2.90E-08	-0.008	0.018	0.666
rs75120545	C	T	0.278	0.035	1.10E-17	0.053	0.040	0.194

rs75227249	A	T	-0.115	0.017	2.40E-11	-0.012	0.019	0.531
rs75601727	G	A	-0.131	0.019	1.90E-13	-0.009	0.021	0.674
rs7596847	T	A	0.084	0.015	3.50E-08	0.025	0.017	0.140
rs7744813	C	A	0.218	0.012	4.70E-84	-0.033	0.012	0.008
rs77740144	G	A	-0.109	0.016	7.10E-12	0.037	0.019	0.056
rs7816934	T	C	0.104	0.019	3.70E-08	-0.029	0.019	0.138
rs781832	T	C	0.065	0.012	4.60E-08	-0.005	0.013	0.720
rs7848891	T	A	0.097	0.016	6.10E-11	0.003	0.017	0.850
rs7849585	G	T	0.067	0.012	2.90E-08	-0.014	0.014	0.309
rs78857879	G	A	0.136	0.019	9.10E-14	-0.063	0.021	0.003
rs7944541	G	T	0.114	0.013	7.30E-19	-0.021	0.014	0.152
rs807037	G	C	0.067	0.012	1.90E-08	-0.003	0.013	0.845
rs8086861	A	G	-0.104	0.014	1.20E-12	0.003	0.015	0.869
rs8108157	T	C	0.068	0.012	2.00E-09	0.002	0.013	0.852
rs8132840	A	G	-0.084	0.011	2.80E-13	-0.002	0.012	0.860
rs854087	C	T	0.062	0.012	4.40E-08	0.003	0.013	0.851
rs893363	G	A	0.078	0.012	1.00E-11	-0.009	0.013	0.483
rs893819	A	G	0.076	0.012	1.20E-10	0.008	0.014	0.565
rs9038	T	C	0.061	0.011	1.60E-08	0.047	0.014	0.001
rs9395623	T	A	-0.094	0.012	2.10E-15	-0.003	0.013	0.844
rs943439	C	T	0.061	0.012	2.40E-08	0.008	0.013	0.553
rs9585327	G	A	0.123	0.011	8.90E-28	0.001	0.012	0.964
rs9611597	A	T	0.112	0.016	7.50E-13	0.006	0.018	0.750
rs9824877	G	A	0.099	0.014	1.20E-12	-0.020	0.016	0.195
rs9902531	G	A	-0.092	0.017	2.10E-08	-0.018	0.019	0.325
rs9934586	A	G	-0.121	0.019	1.60E-10	0.012	0.021	0.548
rs999951	C	G	-0.077	0.013	1.50E-09	0.000	0.015	0.987

Supplementary Table S4. Genetic instruments (66-SNP - P<5.0x10^{-8}) for T2D. Abbreviations: SNP, single nucleotide polymorphism; EA, effect allele; OA, other allele; T2D, type II diabetes; se, standard error.

SNP	EA	OA	beta. T2D	se. T2D	pval. T2D	beta. Cataract	se. Cataract	pval. Cataract
rs10184004	T	C	0.079	0.010	5.10E-14	0.010	0.012	0.419
rs10228456	T	C	-0.077	0.010	1.00E-13	-0.001	0.012	0.936
rs10411648	A	T	-0.089	0.012	3.10E-13	0.000	0.015	0.994
rs10830963	G	C	-0.096	0.012	5.90E-17	0.004	0.014	0.772
rs10965246	C	T	0.160	0.014	5.00E-32	0.000	0.016	0.981
rs11257655	T	C	-0.094	0.013	1.40E-13	0.003	0.015	0.826
rs112674299	T	C	0.099	0.015	6.00E-11	-0.010	0.017	0.551
rs11602873	T	A	0.120	0.014	2.00E-18	0.012	0.017	0.499
rs11658063	G	C	0.089	0.011	3.50E-17	0.023	0.013	0.069
rs116782923	T	A	-0.180	0.023	2.00E-15	-0.046	0.029	0.110
rs11720108	T	C	0.088	0.012	1.40E-13	0.020	0.015	0.181
rs11759026	G	A	-0.075	0.012	1.30E-09	0.011	0.014	0.433
rs11763876	G	A	-0.098	0.017	7.50E-09	0.059	0.020	0.003
rs1182395	G	T	-0.064	0.011	3.90E-09	-0.014	0.013	0.259
rs12611068	C	T	0.061	0.011	5.60E-09	0.001	0.013	0.942
rs12967878	C	T	-0.078	0.012	1.90E-10	-0.005	0.014	0.733
rs13262861	A	C	0.100	0.014	7.40E-14	0.003	0.016	0.868
rs13414381	C	T	0.120	0.015	2.40E-15	-0.002	0.019	0.908
rs1359790	A	G	0.097	0.011	9.20E-18	0.000	0.014	0.973
rs1421085	C	T	-0.120	0.010	2.70E-31	0.000	0.012	0.980
rs1496653	G	A	0.097	0.013	2.10E-14	-0.005	0.015	0.737
rs17036160	T	C	0.100	0.016	2.90E-10	-0.013	0.019	0.483
rs17513135	T	C	-0.079	0.012	9.40E-11	0.013	0.014	0.365
rs1800961	T	C	-0.210	0.030	8.70E-13	0.011	0.035	0.746
rs2102278	G	A	-0.060	0.011	4.80E-08	0.010	0.013	0.466
rs2258238	T	A	-0.120	0.017	9.70E-13	-0.013	0.019	0.505
rs2278524	A	G	-0.062	0.011	2.20E-08	0.000	0.013	0.978
rs243018	G	C	-0.064	0.010	9.90E-10	-0.001	0.012	0.931
rs2479038	G	A	0.340	0.056	1.50E-09	-0.009	0.065	0.884
rs2510078	T	C	-0.066	0.011	9.70E-09	-0.008	0.014	0.559
rs2796441	A	G	0.086	0.010	1.80E-16	-0.002	0.013	0.907
rs28678152	C	T	0.067	0.012	1.20E-08	-0.023	0.014	0.112
rs2972144	G	A	-0.086	0.011	1.80E-15	0.014	0.013	0.254

rs34715063	C	T	-0.100	0.015	1.40E-11	-0.019	0.019	0.331
rs34744311	T	C	0.110	0.011	1.80E-26	-0.011	0.013	0.389
rs348330	A	G	0.062	0.011	5.80E-09	0.015	0.013	0.246
rs34872471	C	T	-0.340	0.011	1.00E-200	0.025	0.013	0.066
rs374722	A	G	0.079	0.014	4.50E-08	-0.005	0.017	0.767
rs3802177	A	G	0.110	0.011	9.00E-22	0.004	0.013	0.788
rs465002	T	C	-0.077	0.012	6.30E-11	-0.011	0.014	0.443
rs4689394	G	C	-0.096	0.011	4.90E-20	0.010	0.014	0.481
rs4729854	A	T	-0.061	0.011	6.30E-09	0.006	0.013	0.613
rs4886876	T	C	-0.081	0.012	7.80E-12	-0.001	0.014	0.960
rs5215	T	C	0.074	0.011	7.10E-12	-0.008	0.013	0.555
rs55891333	G	T	-0.099	0.018	2.10E-08	0.002	0.022	0.911
rs614730	A	T	-0.072	0.013	8.50E-09	-0.018	0.015	0.221
rs6446298	C	T	-0.060	0.011	3.70E-08	0.003	0.013	0.802
rs6777684	G	A	-0.070	0.011	2.80E-11	0.004	0.012	0.719
rs6885132	G	C	0.120	0.017	5.70E-12	0.020	0.021	0.329
rs6905775	A	G	-0.100	0.014	1.10E-12	0.005	0.017	0.780
rs703972	C	G	0.084	0.010	3.10E-16	0.019	0.015	0.191
rs7125213	G	A	0.079	0.014	1.90E-08	-0.044	0.016	0.008
rs72631105	A	G	-0.077	0.013	6.90E-09	0.003	0.016	0.860
rs7274168	T	C	-0.062	0.010	1.70E-09	0.000	0.012	0.982
rs72802365	C	G	0.160	0.019	6.40E-16	0.030	0.023	0.179
rs72892910	T	G	-0.085	0.014	4.60E-10	0.009	0.016	0.594
rs7633675	G	T	-0.120	0.011	1.20E-25	0.014	0.013	0.276
rs76895963	G	T	0.500	0.040	2.40E-36	0.078	0.049	0.113
rs7766070	A	C	-0.130	0.012	1.90E-29	-0.002	0.014	0.865
rs780094	C	T	-0.080	0.011	2.80E-14	-0.010	0.012	0.438
rs7807512	T	C	-0.067	0.011	3.00E-09	-0.006	0.014	0.637
rs79687284	C	G	-0.210	0.028	1.90E-13	0.009	0.033	0.789
rs7988007	C	A	0.078	0.014	8.50E-09	0.012	0.016	0.447
rs849135	A	G	0.095	0.010	3.50E-20	-0.006	0.012	0.617
rs896854	C	T	0.064	0.010	3.90E-10	0.039	0.012	0.001
rs9379084	A	G	0.130	0.017	5.70E-15	0.033	0.020	0.098

Supplementary Table S5. Genetic instruments (20-SNP - $P<5.0x10^{-8}$) for SBP. Abbreviations: SNP, single nucleotide polymorphism; EA, effect allele; OA, other allele; SBP systolic blood pressure; se, standard error.

SNP	EA	OA	beta. SBP	se. SBP	pval. SBP	beta. Cataract	se. Cataract	pval. Cataract
rs10219559	T	C	0.342	0.061	2.20E-08	0.005	0.009	0.575
rs11105352	G	A	0.571	0.082	2.50E-12	-0.008	0.011	0.488
rs11191548	T	C	0.821	0.105	5.10E-15	0.020	0.016	0.203
rs12509595	T	C	-0.469	0.066	9.10E-13	0.002	0.009	0.812
rs12712886	C	G	0.349	0.061	1.30E-08	0.003	0.009	0.744
rs1275984	A	C	0.564	0.061	2.50E-20	0.004	0.009	0.627
rs13021222	G	C	0.462	0.067	6.20E-12	0.010	0.009	0.274
rs13107325	C	T	0.646	0.113	1.10E-08	0.004	0.016	0.825
rs2307032	T	C	0.356	0.063	1.40E-08	-0.027	0.009	0.003
rs2392929	T	G	-0.425	0.075	1.10E-08	-0.002	0.011	0.883
rs4688508	T	C	0.335	0.060	1.90E-08	0.013	0.009	0.130
rs486023	G	A	0.425	0.066	1.40E-10	0.005	0.009	0.614
rs55892892	C	A	0.913	0.132	5.60E-12	-0.036	0.019	0.053
rs6108787	T	G	-0.334	0.059	1.80E-08	-0.003	0.008	0.749
rs6787345	A	G	-0.388	0.063	9.80E-10	0.001	0.009	0.899
rs7111257	A	G	-0.452	0.070	7.70E-11	-0.019	0.010	0.056
rs7115331	T	G	-0.379	0.066	7.30E-09	0.014	0.009	0.122
rs7497418	G	A	-0.348	0.062	1.70E-08	-0.012	0.009	0.181
rs7700842	T	C	0.333	0.061	5.00E-08	-0.001	0.009	0.886
rs8105753	A	C	0.342	0.062	3.80E-08	-0.001	0.009	0.875

Supplementary Table S6. Genetic instruments (20-SNP - $P<5.0x10^{-8}$) for DBP. Abbreviations: SNP, single nucleotide polymorphism; EA, effect allele; OA, other allele; DBP diastolic blood pressure; se, standard error.

SNP	EA	OA	beta. DBP	se. DBP	pval. DBP	beta. Cataract	se. Cataract	pval. Cataract
rs10427021	T	G	0.347	0.058	2.70E-09	-0.003	0.015	0.832
rs10747570	A	G	0.245	0.040	7.30E-10	0.008	0.009	0.379
rs12509595	T	C	-0.363	0.043	1.80E-17	0.002	0.009	0.812
rs12654806	A	G	-0.234	0.039	1.90E-09	-0.014	0.008	0.109
rs13021222	G	C	0.245	0.044	1.90E-08	0.010	0.009	0.274
rs13107325	C	T	0.477	0.073	8.10E-11	0.004	0.016	0.825
rs142570686	G	T	-1.102	0.189	5.50E-09	-0.048	0.031	0.124
rs150446556	T	C	0.265	0.043	1.00E-09	-0.008	0.009	0.392
rs1731249	T	A	0.375	0.040	4.30E-21	0.003	0.009	0.729
rs17637472	G	A	-0.258	0.040	1.70E-10	-0.020	0.009	0.018
rs17791208	G	T	0.277	0.047	3.30E-09	-0.011	0.010	0.280
rs258317	C	T	0.224	0.040	1.70E-08	-0.012	0.009	0.155
rs486023	G	A	0.249	0.043	7.40E-09	0.005	0.009	0.614
rs60228621	C	T	-0.249	0.042	4.30E-09	-0.003	0.009	0.730
rs6429422	T	G	-0.300	0.042	4.60E-13	-0.001	0.009	0.940
rs653178	C	T	0.301	0.039	5.50E-15	-0.013	0.008	0.128
rs6739828	T	G	0.216	0.039	2.40E-08	0.004	0.008	0.639
rs6787345	A	G	-0.281	0.041	8.50E-12	0.001	0.009	0.899
rs7432737	C	T	-0.404	0.057	1.00E-12	0.009	0.012	0.434
rs77098653	G	A	0.260	0.043	2.00E-09	0.016	0.009	0.098

Supplementary Table S7. Genetic instruments (153-SNP - $P<5.0x10^{-8}$) for BMI. Abbreviations: SNP, single nucleotide polymorphism; EA, effect allele; OA, other allele; BMI, body mass index; se, standard error.

SNP	EA	OA	beta. BMI	se. BMI	pval. BMI	beta. Cataract	se. Cataract	pval. Cataract
rs10063334	T	C	-0.027	0.004	1.90E-09	-0.014	0.011	0.211
rs10132280	C	A	0.022	0.003	6.20E-15	-0.020	0.009	0.027
rs10182181	A	G	-0.031	0.003	3.20E-35	0.011	0.008	0.177
rs10192119	T	G	-0.018	0.003	2.80E-08	-0.003	0.012	0.769
rs10460960	G	A	-0.025	0.004	1.30E-10	-0.003	0.013	0.823
rs10499694	A	G	0.014	0.003	3.60E-08	0.013	0.008	0.136
rs10733682	A	G	0.019	0.003	7.50E-13	-0.005	0.009	0.583
rs10742752	T	C	-0.015	0.003	3.90E-09	-0.007	0.009	0.432
rs1074657	T	C	0.017	0.003	7.70E-09	0.003	0.009	0.760
rs10840100	A	G	-0.018	0.003	5.10E-12	-0.003	0.009	0.753
rs10842239	T	C	-0.023	0.004	8.60E-09	-0.015	0.013	0.249
rs10866869	A	G	0.028	0.005	3.50E-09	0.017	0.014	0.244
rs10883521	A	T	-0.023	0.003	4.90E-11	0.001	0.010	0.958
rs10909880	C	T	0.015	0.003	1.00E-08	0.002	0.009	0.819
rs10920678	A	G	0.017	0.003	6.80E-11	0.005	0.009	0.581
rs10929925	C	A	0.014	0.003	1.80E-08	0.004	0.009	0.617
rs10938397	A	G	-0.037	0.003	1.40E-47	0.002	0.009	0.799
rs10968576	A	G	-0.025	0.003	6.50E-21	0.002	0.009	0.821
rs11057405	G	A	0.029	0.005	2.90E-10	0.012	0.014	0.400
rs1106908	G	A	0.018	0.003	1.60E-12	0.013	0.008	0.139
rs11074452	C	G	0.017	0.003	2.00E-09	0.017	0.008	0.042
rs11081818	G	A	-0.016	0.003	6.40E-09	-0.010	0.008	0.238
rs11165643	C	T	-0.019	0.003	2.60E-13	-0.018	0.009	0.038
rs11170468	A	C	0.017	0.003	1.20E-08	-0.011	0.010	0.291
rs1126313	C	A	-0.029	0.005	3.50E-08	0.010	0.010	0.356
rs11611246	G	T	-0.022	0.003	1.80E-12	-0.007	0.010	0.479
rs11655589	A	G	-0.015	0.003	4.20E-08	-0.006	0.009	0.485
rs1167827	A	G	-0.019	0.003	3.10E-12	-0.016	0.009	0.053
rs11709077	G	A	-0.024	0.004	1.70E-10	-0.016	0.013	0.229
rs11727676	T	C	0.029	0.005	7.20E-09	0.003	0.014	0.855
rs118067556	C	T	0.044	0.007	5.60E-09	-0.021	0.025	0.411
rs11851122	A	G	-0.019	0.003	5.40E-10	-0.021	0.009	0.024
rs12220375	T	C	-0.033	0.004	5.10E-15	0.021	0.016	0.177

rs12286929	A	G	-0.021	0.003	5.50E-16	-0.015	0.008	0.081
rs12352785	A	C	0.018	0.003	5.00E-11	-0.008	0.009	0.385
rs12429545	G	A	-0.032	0.004	4.60E-18	0.020	0.013	0.112
rs12887636	T	G	0.017	0.003	2.20E-10	-0.006	0.009	0.469
rs12939549	A	G	0.017	0.003	2.60E-11	0.009	0.008	0.308
rs13021737	A	G	-0.058	0.003	3.80E-69	-0.004	0.011	0.711
rs13107325	C	T	-0.051	0.005	3.20E-21	0.004	0.016	0.825
rs13191362	A	G	0.025	0.004	2.10E-10	0.009	0.013	0.460
rs1396141	C	T	-0.017	0.003	1.20E-08	0.012	0.009	0.177
rs1421085	T	C	-0.079	0.003	1.00E-200	0.015	0.009	0.088
rs1436351	G	T	-0.016	0.003	1.40E-08	-0.007	0.010	0.440
rs1439620	A	G	0.015	0.003	5.00E-08	-0.002	0.009	0.851
rs1441264	G	A	-0.018	0.003	2.10E-12	-0.007	0.009	0.454
rs144839874	A	G	0.139	0.025	2.00E-08	0.041	0.062	0.509
rs1561277	C	A	0.017	0.003	3.00E-09	0.019	0.010	0.045
rs1561589	G	A	-0.017	0.003	6.60E-10	0.006	0.009	0.492
rs1666132	C	T	0.018	0.003	7.30E-10	-0.001	0.009	0.895
rs17016673	C	G	-0.028	0.004	5.10E-10	-0.001	0.014	0.947
rs17024393	T	C	-0.053	0.007	6.40E-13	-0.059	0.027	0.028
rs17066856	T	C	0.035	0.004	2.70E-16	-0.019	0.015	0.191
rs17203016	A	G	-0.020	0.003	1.10E-09	0.004	0.011	0.735
rs17405819	T	C	0.025	0.003	9.20E-20	0.001	0.009	0.935
rs17630235	G	A	0.016	0.003	5.00E-10	0.003	0.009	0.691
rs17759796	C	A	-0.020	0.004	2.50E-08	0.019	0.012	0.115
rs1819844	A	G	0.019	0.003	4.40E-09	0.001	0.011	0.956
rs188275	A	C	0.021	0.004	1.50E-08	-0.008	0.012	0.504
rs1928295	T	C	0.016	0.003	1.10E-10	-0.004	0.008	0.651
rs197374	C	T	-0.016	0.003	1.60E-08	0.010	0.009	0.271
rs2062332	G	A	-0.015	0.003	7.90E-09	0.005	0.008	0.536
rs2112347	T	G	0.027	0.003	3.00E-26	-0.004	0.009	0.621
rs2145272	G	A	-0.018	0.003	6.30E-11	0.006	0.009	0.473
rs215607	G	A	0.024	0.004	2.00E-10	0.021	0.010	0.040
rs2196618	A	G	-0.019	0.003	9.90E-09	-0.002	0.010	0.860
rs2216931	C	A	-0.020	0.003	1.70E-11	-0.007	0.009	0.416
rs2245368	C	T	0.025	0.004	5.00E-09	0.001	0.011	0.914
rs2270204	T	G	-0.018	0.003	4.50E-09	-0.016	0.010	0.109
rs2287019	C	T	0.031	0.003	1.30E-19	-0.008	0.011	0.479

rs2365389	C	T	0.020	0.003	3.40E-15	0.002	0.009	0.817	
rs2372716	C	T	0.017	0.003	3.10E-08	-0.003	0.010	0.805	
rs2481665	T	C	0.018	0.003	1.40E-12	0.001	0.008	0.891	
rs2528531	A	C	-0.017	0.003	1.10E-08	-0.001	0.009	0.865	
rs2759315	C	A	-0.019	0.003	5.20E-11	0.000	0.008	0.995	
rs2820311	A	G	-0.028	0.003	2.60E-20	0.010	0.009	0.246	
rs2836754	T	C	-0.017	0.003	7.40E-11	-0.003	0.009	0.747	
rs2862996	G	T	0.018	0.003	4.30E-11	-0.002	0.009	0.843	
rs2890652	T	C	-0.024	0.004	3.90E-10	0.024	0.011	0.035	
rs29941	A	G	-0.016	0.003	2.50E-09	-0.006	0.009	0.483	
rs3026101	T	C	-0.020	0.003	2.70E-13	-0.007	0.009	0.464	
rs3101336	T	C	-0.032	0.003	7.40E-36	0.010	0.009	0.231	
rs3127553	G	A	0.021	0.003	2.90E-16	0.002	0.009	0.830	
rs34045288	C	T	-0.018	0.003	3.00E-09	0.017	0.009	0.058	
rs34811474	G	A	0.020	0.003	1.60E-09	0.002	0.010	0.837	
rs34976806	C	T	0.017	0.003	7.30E-10	-0.007	0.009	0.410	
rs3764400	T	C	-0.027	0.004	6.00E-13	-0.001	0.013	0.908	
rs3817334	C	T	-0.023	0.003	6.30E-20	0.030	0.009	0.000	
rs4130548	T	C	-0.022	0.003	2.40E-16	0.004	0.009	0.617	
rs4671328	T	G	0.024	0.003	4.90E-18	-0.004	0.009	0.650	
rs4715213	C	T	-0.040	0.003	8.70E-35	-0.015	0.011	0.196	
rs4740619	T	C	0.016	0.003	3.60E-10	-0.013	0.008	0.138	
rs4757142	G	A	-0.019	0.003	1.20E-10	0.003	0.009	0.738	
rs4776970	A	T	0.026	0.003	3.50E-24	-0.010	0.009	0.268	
rs4788099	A	G	-0.029	0.003	9.00E-30	-0.015	0.009	0.083	
rs4820408	T	G	0.016	0.003	3.30E-10	0.004	0.009	0.648	
rs4833079	T	C	0.016	0.003	2.10E-10	-0.014	0.009	0.098	
rs487152	C	A	-0.018	0.003	2.20E-12	0.008	0.008	0.357	
rs492400	C	T	0.014	0.003	4.10E-08	0.002	0.009	0.845	
rs4986044	C	T	0.015	0.003	3.20E-08	-0.001	0.008	0.951	
rs543874	A	G	-0.048	0.003	4.20E-52	0.003	0.010	0.743	
rs592483	C	T	0.016	0.003	1.20E-09	0.004	0.009	0.611	
rs6091540	C	T	0.023	0.003	4.40E-16	-0.008	0.009	0.412	
rs6142096	G	A	-0.016	0.003	1.10E-08	-0.020	0.008	0.020	
rs6265	C	T	0.042	0.003	1.60E-39	-0.015	0.011	0.162	
rs6471932	T	A	0.024	0.004	1.60E-08	0.012	0.013	0.338	
rs6477694	C	T	0.016	0.003	2.10E-10	0.009	0.009	0.322	

rs6545714	G	A	0.018	0.003	1.70E-12	0.007	0.009	0.433
rs6567160	T	C	-0.052	0.003	6.80E-70	0.020	0.010	0.042
rs6569648	C	T	0.016	0.003	4.30E-08	-0.024	0.010	0.015
rs6676190	C	T	0.015	0.003	2.70E-08	0.000	0.009	0.992
rs6713510	G	A	-0.016	0.003	6.20E-10	0.015	0.008	0.073
rs6804842	A	G	-0.017	0.003	5.70E-12	0.006	0.009	0.482
rs6864049	A	G	-0.014	0.003	3.80E-08	0.000	0.009	0.995
rs6870983	C	T	0.020	0.003	8.70E-12	0.028	0.010	0.006
rs687339	C	T	-0.017	0.003	4.70E-08	-0.012	0.010	0.233
rs7138803	G	A	-0.027	0.003	6.10E-27	0.007	0.009	0.406
rs7143963	C	T	-0.021	0.003	8.20E-11	0.023	0.011	0.042
rs7144011	G	T	-0.029	0.003	3.00E-20	0.000	0.010	0.976
rs7161194	A	G	0.019	0.003	1.50E-09	0.022	0.009	0.019
rs7193901	T	C	0.021	0.004	2.70E-08	-0.006	0.012	0.637
rs7239883	G	A	0.016	0.003	6.80E-10	0.016	0.009	0.063
rs749671	G	A	0.018	0.003	1.80E-12	-0.014	0.009	0.114
rs756717	G	A	0.015	0.003	2.40E-08	-0.004	0.009	0.687
rs7580766	A	G	-0.016	0.003	4.30E-08	0.008	0.008	0.373
rs758747	C	T	-0.018	0.003	1.50E-09	-0.020	0.010	0.039
rs7599312	G	A	0.021	0.003	4.40E-14	-0.016	0.010	0.103
rs7613875	C	A	-0.020	0.003	6.90E-14	-0.006	0.008	0.480
rs7629375	C	A	0.021	0.003	3.50E-17	-0.004	0.009	0.685
rs7647305	T	C	-0.036	0.003	7.20E-31	0.006	0.010	0.536
rs77165542	C	T	0.061	0.008	1.10E-14	-0.043	0.023	0.065
rs7730898	G	A	-0.016	0.003	5.10E-09	0.007	0.009	0.442
rs7899106	A	G	-0.039	0.006	2.70E-11	0.025	0.019	0.193
rs7903146	C	T	0.021	0.003	3.30E-14	0.026	0.009	0.005
rs80255756	G	A	-0.039	0.006	1.20E-09	0.023	0.017	0.189
rs8036171	A	C	0.016	0.003	1.00E-09	-0.009	0.009	0.304
rs815610	C	G	0.020	0.003	2.20E-12	0.000	0.008	0.959
rs879620	C	T	-0.025	0.003	4.00E-14	-0.012	0.009	0.176
rs889398	C	T	0.016	0.003	1.70E-10	-0.044	0.009	0.000
rs891389	C	T	-0.021	0.003	1.90E-12	0.005	0.009	0.583
rs901630	C	T	0.014	0.003	2.50E-08	0.000	0.009	0.984
rs935166	G	A	0.017	0.003	5.40E-10	-0.005	0.008	0.538
rs9400239	T	C	-0.020	0.003	4.80E-14	-0.023	0.009	0.014
rs9462027	G	A	-0.020	0.003	4.80E-13	0.004	0.010	0.654

rs946711	A	C	-0.018	0.003	3.50E-09	0.006	0.009	0.489
rs947612	G	A	0.018	0.003	1.60E-08	-0.001	0.010	0.879
rs9512699	G	A	0.025	0.003	1.90E-12	0.006	0.011	0.544
rs9513140	C	T	-0.019	0.003	3.10E-11	0.026	0.008	0.002
rs9527895	T	C	-0.024	0.004	8.00E-10	-0.014	0.011	0.217
rs9563576	C	T	0.022	0.003	9.70E-12	-0.013	0.011	0.253
rs972540	A	G	-0.018	0.003	1.70E-10	-0.001	0.009	0.938
rs977747	T	G	0.017	0.003	1.80E-11	-0.011	0.009	0.203
rs9856151	G	A	-0.017	0.003	3.20E-09	0.002	0.009	0.814

Supplementary Table S8. Genetic instruments (231-SNP - $P<5.0x10^{-8}$) for cigarette smoking initiation. Abbreviations: SNP, single nucleotide polymorphism; EA, effect allele; OA, other allele; SmkInit, smoking initiation; se, standard error.

SNP	EA	OA	beta. SmkInit	se. SmkInit	pval. SmkInit	beta. Cataract	se. Cataract	pval. Cataract
rs10001365	A	G	-0.015	0.002	4.32E-21	-0.011	0.012	0.386
rs1004787	A	G	0.016	0.002	3.91E-24	-0.006	0.012	0.619
rs10062607	A	C	0.010	0.002	2.28E-10	-0.003	0.013	0.825
rs10119117	T	C	0.009	0.002	1.80E-08	-0.001	0.012	0.925
rs10121930	A	T	0.010	0.002	8.01E-10	-0.019	0.012	0.109
rs10233018	G	A	0.014	0.002	1.49E-18	0.004	0.012	0.751
rs1025910	C	G	-0.012	0.002	4.83E-13	0.013	0.013	0.322
rs10279261	A	G	-0.012	0.002	6.87E-14	-0.003	0.012	0.844
rs10444314	G	T	-0.009	0.002	1.77E-08	-0.001	0.012	0.962
rs10458563	G	A	0.017	0.002	2.17E-18	0.022	0.015	0.148
rs1050847	T	C	-0.011	0.002	3.71E-11	0.015	0.014	0.271
rs10698713	A	G	-0.020	0.004	1.84E-08	-0.001	0.027	0.984
rs10745324	G	A	-0.010	0.002	1.97E-08	0.034	0.013	0.009
rs10753630	T	C	0.011	0.002	9.96E-11	0.016	0.013	0.213
rs10786721	A	C	0.018	0.002	7.91E-27	-0.006	0.012	0.646
rs1084445	C	T	-0.012	0.002	1.80E-10	0.013	0.015	0.382
rs10914684	A	G	-0.011	0.002	3.24E-10	0.039	0.013	0.003
rs10927039	C	T	-0.013	0.002	2.39E-10	0.010	0.016	0.534
rs10954115	C	A	-0.010	0.002	4.78E-09	0.015	0.013	0.219
rs11012726	C	T	0.014	0.002	4.25E-17	-0.008	0.013	0.550
rs11078713	G	A	-0.013	0.002	2.77E-15	0.008	0.012	0.510
rs1111578	T	G	-0.013	0.002	8.22E-09	-0.024	0.017	0.153
rs11130381	T	C	-0.009	0.002	4.83E-08	0.015	0.013	0.261
rs11162019	T	C	-0.010	0.002	3.40E-09	-0.001	0.013	0.957
rs11162976	C	A	0.011	0.002	6.47E-10	0.003	0.014	0.808
rs111861749	A	G	-0.018	0.003	3.16E-12	-0.023	0.020	0.242
rs11210229	G	A	-0.014	0.002	4.94E-18	-0.019	0.012	0.118
rs11258417	T	C	-0.010	0.002	3.83E-10	0.000	0.013	0.973
rs114050142	A	T	-0.023	0.003	2.56E-11	-0.033	0.028	0.242
rs114900182	G	C	-0.021	0.003	1.45E-12	0.007	0.025	0.791
rs1155641	A	G	0.010	0.002	7.72E-10	0.000	0.013	0.993
rs11632439	G	A	0.009	0.002	1.31E-08	-0.009	0.012	0.444
rs11656151	G	A	-0.013	0.002	5.61E-12	-0.034	0.015	0.019

rs11673452	C	T	0.015	0.003	3.40E-09	0.028	0.020	0.155
rs11693702	A	T	0.014	0.002	9.33E-19	-0.004	0.012	0.755
rs11695197	A	G	0.014	0.002	6.28E-09	0.026	0.021	0.222
rs11716705	G	A	0.012	0.002	1.40E-10	-0.015	0.014	0.270
rs11720703	T	C	0.011	0.002	5.58E-12	-0.011	0.012	0.354
rs11742625	C	T	0.016	0.002	2.19E-10	0.020	0.018	0.275
rs11756490	A	T	-0.014	0.002	6.20E-09	0.004	0.019	0.837
rs11873164	T	C	-0.016	0.002	1.63E-12	-0.011	0.017	0.526
rs11926232	G	A	0.021	0.004	1.16E-08	0.006	0.029	0.832
rs12027999	C	T	-0.018	0.002	1.54E-13	0.023	0.019	0.223
rs12036050	C	T	0.012	0.002	7.18E-11	0.011	0.013	0.427
rs12079063	G	A	-0.009	0.002	3.88E-08	-0.020	0.012	0.100
rs12112638	G	A	-0.012	0.002	8.74E-12	-0.026	0.014	0.053
rs12133063	A	C	0.012	0.002	1.15E-12	-0.003	0.013	0.820
rs12209775	G	C	-0.010	0.002	1.29E-10	-0.011	0.012	0.364
rs12213996	A	G	0.012	0.002	2.89E-13	-0.001	0.013	0.917
rs12441907	A	C	-0.015	0.002	6.09E-14	-0.029	0.016	0.065
rs1246265	C	T	0.012	0.002	1.27E-11	0.016	0.013	0.237
rs12485391	A	C	0.014	0.002	1.89E-08	-0.019	0.018	0.303
rs12600466	T	A	0.012	0.002	1.30E-11	0.003	0.013	0.825
rs12632110	G	A	-0.014	0.002	1.18E-16	-0.010	0.015	0.504
rs12642744	T	G	-0.012	0.002	5.98E-11	-0.011	0.014	0.449
rs12685816	A	G	0.015	0.002	1.66E-10	-0.007	0.018	0.675
rs12714017	C	T	0.010	0.002	6.57E-10	0.005	0.012	0.651
rs1291865	T	G	0.012	0.002	2.12E-13	0.008	0.012	0.508
rs12923427	T	C	-0.014	0.002	4.96E-12	0.007	0.015	0.632
rs13009008	G	A	-0.010	0.002	6.92E-09	-0.002	0.013	0.868
rs13030994	A	G	0.021	0.002	2.42E-39	0.008	0.012	0.520
rs13145728	C	G	-0.012	0.002	6.87E-14	0.003	0.013	0.821
rs13162305	T	A	0.010	0.002	1.15E-08	-0.008	0.013	0.562
rs13246563	G	C	-0.013	0.002	3.99E-14	0.000	0.012	0.996
rs13261725	C	G	0.013	0.002	3.43E-13	0.006	0.014	0.651
rs13301073	A	G	0.011	0.002	3.13E-11	-0.008	0.013	0.528
rs1334557	T	C	0.013	0.002	1.51E-12	0.007	0.014	0.633
rs13377168	A	T	-0.015	0.002	2.16E-20	-0.002	0.012	0.888
rs13398418	A	T	0.012	0.002	2.09E-11	-0.011	0.014	0.424
rs134529	C	T	-0.012	0.002	8.86E-13	-0.004	0.013	0.738

rs1381287	T	C	0.012	0.002	7.51E-15	-0.018	0.012	0.145
rs1392446	T	C	0.010	0.002	5.60E-10	0.009	0.012	0.456
rs1435679	A	G	0.010	0.002	2.86E-10	-0.005	0.012	0.714
rs143909875	C	T	0.018	0.003	3.74E-12	-0.001	0.020	0.947
rs147052174	T	G	0.034	0.006	1.31E-08	-0.017	0.051	0.733
rs1485272	C	T	-0.009	0.002	4.07E-08	-0.028	0.013	0.032
rs1499982	T	C	0.022	0.002	3.01E-23	-0.012	0.018	0.509
rs1503211	A	G	0.010	0.002	3.43E-10	0.008	0.012	0.495
rs1559278	C	T	-0.011	0.002	9.97E-12	0.005	0.013	0.702
rs1565735	A	T	-0.023	0.002	7.49E-32	0.000	0.015	0.997
rs160631	G	T	-0.011	0.002	3.56E-09	-0.016	0.014	0.240
rs16896199	T	A	0.012	0.002	5.61E-09	0.014	0.017	0.395
rs16975171	A	C	-0.017	0.003	5.29E-09	0.031	0.022	0.154
rs17207435	G	A	0.019	0.003	1.47E-08	-0.011	0.025	0.652
rs1733756	G	A	0.010	0.002	1.23E-10	0.014	0.012	0.232
rs17432775	T	C	-0.011	0.002	2.55E-08	-0.021	0.015	0.146
rs17594561	G	A	0.010	0.002	1.27E-09	-0.007	0.012	0.570
rs1776631	C	T	-0.011	0.002	3.92E-08	-0.001	0.015	0.930
rs1820083	C	T	0.012	0.002	9.23E-12	-0.001	0.013	0.968
rs1901477	G	A	0.016	0.002	1.94E-22	-0.009	0.012	0.486
rs1937443	G	C	0.014	0.002	1.27E-18	0.002	0.012	0.883
rs1945737	C	T	-0.010	0.002	8.19E-10	0.010	0.012	0.434
rs1971318	T	C	0.016	0.002	7.80E-13	0.013	0.017	0.458
rs1994247	T	G	0.012	0.002	1.25E-14	0.005	0.012	0.655
rs2022815	A	G	0.010	0.002	1.12E-09	-0.012	0.013	0.346
rs2072155	C	T	0.013	0.002	1.16E-13	0.013	0.013	0.316
rs2101364	G	C	-0.010	0.002	3.89E-08	0.015	0.014	0.290
rs2135160	C	T	0.016	0.003	8.23E-09	-0.026	0.020	0.202
rs2163413	G	A	-0.014	0.002	3.06E-11	-0.004	0.016	0.808
rs2173019	A	T	0.013	0.002	3.47E-10	0.029	0.016	0.071
rs2237303	A	G	-0.010	0.002	6.37E-10	-0.003	0.013	0.801
rs2289791	T	G	-0.013	0.002	2.87E-12	0.006	0.014	0.676
rs2292239	G	T	0.011	0.002	1.03E-10	0.008	0.013	0.522
rs2313500	T	C	0.013	0.002	1.99E-12	0.023	0.014	0.109
rs2678903	G	A	0.010	0.002	1.25E-10	-0.002	0.012	0.892
rs2708630	T	C	-0.010	0.002	9.99E-10	0.012	0.013	0.376
rs2711607	T	G	0.011	0.002	4.09E-08	-0.004	0.017	0.805

rs2783130	G	A	-0.009	0.002	2.71E-08	-0.008	0.012	0.504
rs2876586	A	G	0.009	0.002	1.88E-08	-0.032	0.012	0.009
rs288181	T	C	-0.009	0.002	4.11E-08	-0.003	0.013	0.826
rs2939756	A	G	-0.010	0.002	6.87E-11	-0.028	0.012	0.020
rs3110590	A	C	0.011	0.002	1.22E-09	-0.025	0.014	0.071
rs3213876	C	T	0.012	0.002	5.08E-12	-0.003	0.013	0.832
rs326341	A	G	-0.010	0.002	6.16E-10	0.011	0.012	0.361
rs326387	C	T	-0.010	0.002	3.64E-10	-0.016	0.013	0.216
rs332827	A	G	-0.009	0.002	2.81E-08	0.020	0.013	0.111
rs34367058	T	C	0.014	0.002	1.36E-13	-0.004	0.015	0.810
rs34371841	T	C	0.011	0.002	3.37E-08	0.029	0.015	0.042
rs34488670	C	T	0.018	0.002	8.73E-20	-0.006	0.015	0.700
rs35891966	A	G	-0.019	0.003	6.52E-10	-0.028	0.028	0.325
rs3781295	A	G	-0.012	0.002	1.13E-12	0.001	0.013	0.930
rs3801289	C	A	-0.012	0.002	3.64E-12	0.004	0.013	0.748
rs3814994	T	G	0.010	0.002	7.44E-09	0.015	0.013	0.251
rs3895907	G	A	-0.013	0.002	1.56E-14	-0.015	0.012	0.234
rs3905125	T	C	0.010	0.002	4.28E-11	0.023	0.012	0.058
rs3934797	A	G	-0.015	0.002	1.90E-13	-0.044	0.016	0.006
rs39784	A	C	0.012	0.002	6.41E-11	0.026	0.014	0.068
rs4044321	G	A	-0.016	0.002	3.04E-23	-0.026	0.013	0.042
rs4374330	T	C	0.013	0.002	3.73E-12	-0.001	0.014	0.919
rs4401691	G	C	-0.010	0.002	9.42E-09	0.001	0.012	0.947
rs4479577	T	C	0.009	0.002	2.11E-08	0.010	0.012	0.392
rs4543592	C	T	0.012	0.002	2.51E-14	-0.005	0.012	0.681
rs4571506	T	C	-0.015	0.002	1.64E-21	0.022	0.012	0.076
rs4579569	A	G	0.012	0.002	1.23E-14	-0.005	0.012	0.675
rs465646	A	G	-0.024	0.002	1.70E-28	0.025	0.017	0.135
rs4659805	T	G	0.009	0.002	8.36E-09	0.003	0.013	0.800
rs4751614	T	A	0.012	0.002	6.35E-11	0.009	0.014	0.537
rs4785187	A	G	0.012	0.002	2.03E-10	-0.007	0.016	0.667
rs4819027	G	C	-0.010	0.002	1.34E-08	-0.021	0.014	0.127
rs4837631	T	C	-0.009	0.002	3.54E-09	0.009	0.012	0.441
rs4841484	A	G	-0.010	0.002	6.29E-09	0.007	0.013	0.576
rs4857114	T	C	0.009	0.002	3.03E-08	0.005	0.012	0.680
rs4865172	T	C	-0.009	0.002	4.01E-08	-0.005	0.013	0.702
rs4888444	G	A	-0.025	0.004	6.84E-10	-0.041	0.029	0.150

rs4944844	C	T	-0.013	0.002	2.33E-10	0.015	0.016	0.366
rs540356	A	C	0.012	0.002	4.07E-13	-0.038	0.012	0.002
rs540860	G	A	0.012	0.002	3.18E-13	-0.022	0.012	0.070
rs551739	T	A	0.010	0.002	4.42E-09	0.004	0.013	0.749
rs557544	C	T	-0.009	0.002	2.82E-08	0.013	0.013	0.316
rs55921136	C	T	-0.014	0.002	1.90E-12	-0.026	0.015	0.089
rs55942317	A	G	0.017	0.003	4.08E-11	-0.012	0.021	0.565
rs56169608	A	G	0.009	0.002	1.00E-08	-0.004	0.012	0.726
rs56348592	G	A	0.013	0.002	1.81E-08	0.026	0.017	0.121
rs56820925	T	C	-0.011	0.002	2.25E-10	-0.001	0.013	0.922
rs574835	A	G	-0.010	0.002	2.33E-10	-0.025	0.014	0.072
rs5751239	T	C	-0.010	0.002	1.02E-10	0.016	0.012	0.180
rs58400863	A	G	-0.011	0.002	5.03E-12	-0.015	0.013	0.255
rs60453921	A	T	0.013	0.002	1.06E-09	0.014	0.017	0.396
rs6088618	A	G	-0.011	0.002	5.89E-11	0.012	0.012	0.327
rs6141314	A	G	0.013	0.002	3.59E-13	-0.023	0.015	0.116
rs61533748	C	T	0.010	0.002	1.50E-10	0.002	0.013	0.860
rs61959481	A	G	-0.012	0.002	2.06E-10	-0.004	0.015	0.787
rs62107261	C	T	-0.026	0.004	2.13E-11	0.004	0.029	0.881
rs62135525	T	C	-0.022	0.004	4.61E-09	-0.025	0.031	0.417
rs62181771	A	G	0.010	0.002	4.47E-10	0.020	0.012	0.103
rs62250713	G	A	-0.019	0.002	1.63E-30	0.010	0.013	0.426
rs62254171	A	G	0.031	0.005	3.73E-09	0.002	0.042	0.963
rs62258903	G	C	-0.016	0.002	7.20E-12	0.016	0.018	0.356
rs62266876	G	C	0.015	0.003	3.06E-08	0.030	0.021	0.147
rs62419578	A	T	0.013	0.002	1.08E-08	0.014	0.019	0.446
rs6265	T	C	-0.020	0.002	1.86E-22	0.024	0.016	0.121
rs6438208	A	G	-0.010	0.002	7.33E-09	0.019	0.014	0.188
rs6464024	T	C	-0.012	0.002	3.38E-14	0.014	0.013	0.259
rs6472232	G	T	-0.009	0.002	1.43E-08	0.025	0.013	0.046
rs6497840	A	G	0.013	0.002	1.13E-12	-0.007	0.013	0.594
rs6598539	C	T	0.011	0.002	5.76E-12	0.011	0.012	0.367
rs66480876	G	C	0.009	0.002	1.38E-08	0.001	0.012	0.922
rs66680800	T	G	-0.013	0.002	1.13E-14	-0.008	0.012	0.533
rs6705147	T	C	0.010	0.002	5.24E-09	0.016	0.013	0.238
rs6728726	C	T	0.019	0.002	4.27E-19	-0.015	0.016	0.355
rs6852117	G	C	-0.012	0.002	1.97E-13	0.006	0.012	0.647

rs6959670	T	C	0.010	0.002	7.29E-09	0.016	0.013	0.223
rs6973168	G	C	-0.009	0.002	2.70E-09	-0.010	0.012	0.427
rs7026534	G	T	-0.011	0.002	2.46E-10	0.005	0.013	0.690
rs7092291	T	C	-0.010	0.002	7.74E-10	-0.008	0.012	0.524
rs7127712	T	A	0.027	0.002	2.19E-65	-0.007	0.012	0.589
rs71367545	A	G	0.012	0.002	1.70E-10	0.007	0.016	0.636
rs71491832	G	C	-0.018	0.003	3.72E-10	0.029	0.023	0.203
rs71627577	G	A	-0.017	0.002	5.15E-12	-0.027	0.020	0.190
rs7195043	T	C	-0.009	0.002	2.13E-08	0.013	0.014	0.353
rs7205551	A	G	0.010	0.002	1.15E-09	0.021	0.012	0.089
rs7224742	T	C	-0.011	0.002	1.02E-11	-0.014	0.013	0.285
rs72733235	C	T	0.013	0.002	1.86E-09	0.006	0.017	0.711
rs72789627	T	C	-0.017	0.002	1.54E-13	0.000	0.018	0.992
rs72904370	C	G	-0.011	0.002	1.59E-08	-0.001	0.015	0.962
rs7333559	A	G	-0.014	0.002	3.24E-13	-0.019	0.015	0.216
rs75177132	T	C	-0.026	0.004	1.02E-12	-0.051	0.032	0.112
rs7585579	G	C	0.013	0.002	1.29E-14	0.001	0.012	0.936
rs76132272	T	C	0.018	0.003	9.43E-09	-0.001	0.024	0.959
rs7629352	G	A	0.010	0.002	7.82E-09	0.028	0.013	0.037
rs77307359	C	T	-0.011	0.002	2.07E-08	0.004	0.015	0.807
rs7788527	C	T	0.010	0.002	1.58E-08	0.001	0.013	0.956
rs7804551	G	A	-0.015	0.002	1.09E-12	0.034	0.017	0.045
rs78175438	C	T	0.016	0.002	2.02E-11	-0.003	0.018	0.872
rs7829715	C	T	-0.012	0.002	1.30E-13	0.002	0.012	0.859
rs7893954	G	A	-0.010	0.002	1.43E-08	-0.019	0.014	0.165
rs79222572	G	T	0.010	0.002	1.53E-08	0.025	0.014	0.079
rs7929518	G	A	0.012	0.002	9.06E-10	-0.002	0.014	0.894
rs7947391	G	A	0.009	0.002	7.55E-09	-0.025	0.013	0.050
rs7969559	G	A	-0.011	0.002	3.28E-10	-0.004	0.013	0.746
rs7984311	A	G	0.009	0.002	1.77E-08	0.011	0.013	0.391
rs7986094	C	A	0.010	0.002	5.99E-09	0.021	0.013	0.116
rs8001839	G	A	0.009	0.002	3.74E-08	0.006	0.013	0.662
rs8067305	A	G	0.011	0.002	4.95E-12	-0.019	0.013	0.149
rs8069451	C	T	0.011	0.002	1.02E-09	0.019	0.014	0.176
rs846781	C	T	-0.012	0.002	4.80E-11	-0.011	0.014	0.433
rs853684	C	T	-0.012	0.002	8.55E-13	0.011	0.013	0.385
rs888292	T	A	-0.011	0.002	2.03E-09	-0.010	0.013	0.431

rs911781	G	A	-0.009	0.002	4.99E-08	-0.003	0.013	0.783
rs9323328	G	A	-0.011	0.002	7.25E-12	-0.008	0.012	0.523
rs9375371	A	G	0.013	0.002	1.41E-13	0.012	0.014	0.394
rs9402093	T	G	0.010	0.002	3.38E-09	-0.008	0.013	0.570
rs9423279	G	C	-0.011	0.002	9.64E-11	-0.012	0.013	0.371
rs951740	A	G	0.017	0.002	3.78E-26	0.008	0.013	0.499
rs9529052	A	T	-0.012	0.002	1.85E-13	-0.018	0.012	0.144
rs9538536	G	T	-0.009	0.002	4.28E-08	-0.008	0.013	0.537
rs9541499	G	T	-0.017	0.003	2.16E-08	-0.041	0.023	0.075
rs9613472	G	A	0.009	0.002	4.02E-08	0.006	0.013	0.653
rs963354	A	C	0.011	0.002	2.35E-10	0.016	0.013	0.218
rs993700	C	T	-0.014	0.002	1.00E-13	-0.031	0.015	0.034
rs9987376	G	T	-0.014	0.002	1.02E-17	-0.003	0.012	0.789

Supplementary Table S9. Genetic instruments (47-SNP - *P*<5.0x10^{-8}) for cigarettes per day. Abbreviations: SNP, single nucleotide polymorphism; EA, effect allele; OA, other allele; CigDay, cigarettes per day; se, standard error.

SNP	EA	OA	beta. CigDay	se. CigDay	pval. CigDay	beta. Cataract	se. Cataract	pval. Cataract
rs11076320	A	C	-0.016	0.003	1.06E-10	-0.015	0.013	0.239
rs112178027	T	C	0.021	0.003	8.02E-10	-0.017	0.016	0.311
rs112270518	A	G	0.034	0.004	6.01E-18	-0.012	0.020	0.551
rs11663346	T	A	-0.014	0.003	1.75E-08	-0.011	0.012	0.393
rs11686893	C	T	0.017	0.003	3.28E-10	0.021	0.013	0.119
rs11725618	C	T	0.018	0.003	5.71E-11	0.016	0.014	0.257
rs11921010	C	T	0.014	0.003	2.33E-08	0.011	0.012	0.386
rs11928552	C	T	-0.017	0.003	2.71E-09	-0.011	0.013	0.410
rs11940430	A	T	-0.015	0.003	2.94E-08	-0.008	0.013	0.557
rs12660603	C	T	-0.030	0.006	4.24E-08	-0.013	0.027	0.631
rs13254578	C	G	0.035	0.003	4.81E-33	0.000	0.015	0.993
rs138759397	C	G	0.063	0.009	5.71E-13	-0.038	0.048	0.428
rs141147481	G	C	-0.059	0.009	3.61E-10	-0.082	0.050	0.098
rs1444026	G	T	0.014	0.002	2.15E-08	0.010	0.012	0.405
rs145104523	T	C	0.021	0.004	1.09E-08	0.001	0.018	0.958
rs1657936	T	C	-0.019	0.003	6.52E-10	-0.018	0.015	0.234
rs17197116	C	T	0.030	0.005	4.52E-11	-0.032	0.022	0.153
rs1737894	G	C	0.019	0.003	1.69E-14	-0.031	0.013	0.014
rs185771419	T	G	-0.054	0.007	1.20E-13	-0.050	0.045	0.267
rs2016968	G	C	-0.019	0.003	2.04E-14	0.010	0.014	0.469
rs2060220	T	A	0.020	0.004	2.16E-08	0.012	0.018	0.495
rs2072659	G	C	-0.030	0.004	6.44E-13	-0.002	0.023	0.920
rs215600	A	G	-0.023	0.003	2.81E-18	-0.009	0.013	0.465
rs244417	C	T	-0.017	0.003	2.72E-11	-0.027	0.012	0.028
rs2655008	T	A	0.016	0.003	1.78E-08	0.009	0.014	0.503
rs34370696	T	C	-0.019	0.003	2.36E-08	0.008	0.017	0.621
rs34406232	A	C	-0.080	0.007	1.21E-26	0.043	0.039	0.268
rs3796462	T	C	-0.015	0.003	3.11E-08	-0.003	0.013	0.807
rs56113850	C	T	0.062	0.002	1.36E-137	-0.007	0.012	0.597
rs6078372	A	G	0.015	0.003	1.22E-09	0.018	0.012	0.154
rs6603895	A	T	-0.014	0.002	1.55E-08	0.014	0.012	0.257
rs6697255	C	T	-0.015	0.003	6.18E-09	0.009	0.012	0.445
rs6699355	T	C	-0.021	0.004	3.05E-08	0.010	0.020	0.614

rs6831786	A	C	-0.016	0.003	6.51E-10	-0.019	0.012	0.121
rs72738704	C	G	0.086	0.003	1.00E-200	-0.002	0.013	0.887
rs73229090	A	C	0.030	0.004	1.65E-14	-0.007	0.020	0.731
rs7599488	T	C	0.014	0.002	8.42E-09	-0.003	0.012	0.802
rs7678019	A	G	-0.018	0.003	9.60E-11	0.007	0.013	0.601
rs79250609	T	C	-0.019	0.003	7.93E-09	-0.002	0.018	0.907
rs7928017	A	C	-0.018	0.002	2.22E-12	-0.002	0.012	0.846
rs7933830	T	C	0.019	0.003	5.23E-13	-0.004	0.013	0.759
rs7944241	G	A	-0.016	0.003	4.59E-10	0.010	0.012	0.415
rs8021229	T	C	0.018	0.003	2.14E-11	-0.003	0.013	0.846
rs80292109	G	A	-0.019	0.003	1.24E-11	0.003	0.013	0.838
rs806255	A	G	-0.020	0.003	5.53E-12	-0.013	0.014	0.348
rs9522262	G	C	0.014	0.002	6.50E-09	0.014	0.012	0.241
rs9881798	C	A	0.015	0.003	4.47E-09	0.032	0.012	0.009

Supplementary Table S10. Genetic instruments (121-SNP - P<5.0x10^{-8}) for lifetime smoking. Abbreviations: SNP, single nucleotide polymorphism; EA, effect allele; OA, other allele; LifeSmk, lifetime smoking; se, standard error.

SNP	EA	OA	beta. LifeSmk	se. LifeSmk	pval. LifeSmk	beta. Cataract	se. Cataract	pval. Cataract
rs10052591	T	C	0.008	0.001	2.10E-09	-0.009	0.012	0.482
rs10226228	A	G	-0.011	0.001	2.00E-15	-0.010	0.013	0.437
rs10282292	C	T	0.009	0.001	5.90E-10	0.009	0.013	0.465
rs1050847	C	T	0.008	0.001	1.40E-08	-0.015	0.014	0.271
rs10823968	A	T	0.008	0.001	2.10E-08	-0.002	0.013	0.843
rs10879871	T	G	-0.010	0.001	5.00E-11	0.009	0.013	0.492
rs10918701	G	A	0.008	0.001	2.10E-08	0.014	0.013	0.271
rs10922907	A	T	0.010	0.001	3.00E-13	0.008	0.012	0.489
rs11210229	A	G	0.012	0.001	2.00E-16	0.019	0.012	0.118
rs112282219	G	A	-0.023	0.004	3.80E-11	0.019	0.032	0.536
rs11255908	T	G	-0.010	0.002	2.30E-10	-0.005	0.014	0.712
rs113382419	C	A	-0.028	0.002	3.00E-37	0.014	0.021	0.505
rs11768481	C	A	0.009	0.001	9.90E-10	-0.006	0.013	0.665
rs11783093	C	T	0.016	0.002	1.20E-16	0.005	0.017	0.774
rs11948770	T	C	-0.010	0.002	4.90E-10	-0.010	0.014	0.499
rs12202536	A	G	-0.008	0.001	2.80E-09	0.011	0.012	0.353
rs1221148	C	G	0.009	0.001	7.30E-11	-0.004	0.013	0.733
rs12244388	G	A	-0.013	0.001	1.40E-19	0.008	0.013	0.506
rs1246265	T	C	-0.009	0.002	4.20E-09	-0.016	0.013	0.237
rs12481282	G	C	-0.009	0.002	7.80E-09	0.002	0.014	0.861
rs12623702	A	G	-0.010	0.001	7.70E-12	0.017	0.013	0.167
rs12708665	A	G	-0.009	0.002	3.50E-09	0.001	0.013	0.959
rs12831617	C	T	-0.009	0.002	1.90E-08	0.017	0.014	0.231
rs12967855	A	G	0.008	0.001	3.10E-08	-0.001	0.013	0.937
rs13009008	A	G	0.009	0.001	4.60E-09	0.002	0.013	0.868
rs13016665	C	A	-0.008	0.001	1.80E-09	-0.011	0.012	0.357
rs13153393	A	G	-0.014	0.002	2.50E-10	-0.024	0.019	0.211
rs13296519	G	T	-0.010	0.001	8.10E-12	0.002	0.013	0.866
rs136233	A	G	-0.010	0.002	1.80E-08	0.001	0.016	0.959
rs147412694	G	A	-0.012	0.002	2.90E-09	-0.002	0.017	0.888
rs17309874	G	A	-0.011	0.002	9.70E-13	0.011	0.014	0.427
rs17553262	A	C	-0.013	0.002	5.30E-09	0.003	0.020	0.882
rs17576594	G	A	0.011	0.002	1.70E-12	0.008	0.014	0.542

rs1922018	C	T	0.010	0.001	3.00E-12	-0.005	0.013	0.718
rs1931263	G	T	-0.008	0.001	4.00E-08	0.012	0.012	0.323
rs1933270	T	G	0.009	0.001	1.50E-10	0.019	0.013	0.140
rs202645	A	G	-0.010	0.002	3.90E-09	0.030	0.015	0.046
rs2062882	G	A	-0.008	0.001	1.10E-08	-0.004	0.012	0.732
rs2254710	C	A	0.009	0.002	3.50E-08	0.004	0.015	0.801
rs2401924	G	C	0.011	0.001	2.70E-14	0.012	0.012	0.336
rs245774	A	G	-0.009	0.002	7.40E-09	-0.023	0.014	0.103
rs2675638	G	A	0.008	0.001	1.30E-09	0.009	0.012	0.466
rs2678670	A	T	0.009	0.001	3.10E-10	-0.005	0.012	0.695
rs2838834	C	T	-0.009	0.002	6.30E-10	-0.028	0.014	0.035
rs28485305	C	T	0.008	0.001	2.60E-08	0.002	0.013	0.899
rs28635466	G	A	0.008	0.002	2.00E-08	-0.006	0.013	0.660
rs2867112	T	G	0.015	0.002	4.80E-15	-0.012	0.017	0.457
rs2890772	G	T	-0.014	0.001	2.10E-22	-0.019	0.012	0.113
rs2894808	T	A	-0.015	0.003	3.50E-09	0.002	0.022	0.934
rs317021	T	A	-0.012	0.002	1.10E-10	0.017	0.016	0.288
rs326341	G	A	0.009	0.001	1.20E-11	-0.011	0.012	0.361
rs329120	C	T	0.010	0.001	6.30E-12	0.013	0.012	0.292
rs34866095	A	G	-0.009	0.002	1.20E-08	0.004	0.013	0.767
rs348829	G	A	-0.008	0.001	1.10E-08	-0.017	0.013	0.171
rs35169606	T	G	0.009	0.001	1.20E-09	-0.019	0.013	0.146
rs35175834	G	A	-0.016	0.002	4.60E-22	0.007	0.015	0.626
rs359243	T	C	-0.009	0.001	9.50E-10	-0.009	0.012	0.484
rs369230	G	T	-0.009	0.002	1.80E-09	0.001	0.016	0.952
rs3742365	T	C	-0.011	0.001	2.50E-14	0.011	0.013	0.383
rs3769949	T	A	-0.008	0.001	2.50E-09	0.002	0.012	0.847
rs3811038	T	C	-0.010	0.002	8.90E-10	0.007	0.014	0.606
rs3896224	A	G	0.010	0.001	1.10E-11	-0.022	0.012	0.067
rs421983	T	C	0.009	0.001	3.30E-10	-0.041	0.012	0.001
rs4391802	A	G	0.010	0.002	1.40E-11	0.026	0.013	0.050
rs4473348	A	T	-0.010	0.002	6.40E-11	0.000	0.014	0.974
rs4543592	T	C	-0.009	0.001	4.50E-10	0.005	0.012	0.681
rs4568549	C	A	-0.008	0.001	3.90E-08	-0.026	0.012	0.033
rs4571506	C	T	0.008	0.001	1.50E-08	-0.022	0.012	0.076
rs4671357	T	C	-0.009	0.001	1.10E-11	-0.007	0.012	0.558
rs4731925	C	T	-0.008	0.001	2.60E-08	0.005	0.013	0.710

rs4814873	C	T	0.010	0.002	2.90E-09	-0.011	0.015	0.455
rs4957528	A	C	-0.010	0.002	4.20E-09	0.009	0.015	0.533
rs530916	A	G	-0.008	0.001	2.00E-08	0.011	0.012	0.372
rs549845	G	A	0.011	0.002	8.30E-14	0.002	0.013	0.876
rs57611503	G	A	0.008	0.001	4.00E-08	-0.009	0.013	0.493
rs60952428	T	C	0.013	0.002	3.00E-08	-0.012	0.021	0.555
rs6119897	G	A	-0.013	0.002	3.60E-15	0.016	0.015	0.282
rs61796681	A	T	-0.013	0.002	4.20E-08	0.018	0.022	0.421
rs62098013	G	A	-0.009	0.001	4.10E-09	-0.018	0.013	0.169
rs62135536	C	T	0.024	0.004	8.00E-10	0.050	0.038	0.186
rs62155874	A	G	-0.017	0.002	5.20E-16	-0.022	0.019	0.243
rs62175972	T	C	0.022	0.004	1.70E-08	-0.017	0.032	0.595
rs624833	T	G	0.009	0.002	6.60E-10	-0.009	0.013	0.500
rs6562474	C	G	0.008	0.001	1.00E-08	-0.006	0.013	0.614
rs6598539	T	C	-0.008	0.001	4.50E-09	-0.011	0.012	0.367
rs6692614	C	G	-0.008	0.001	2.80E-08	0.009	0.013	0.474
rs6741228	T	C	0.008	0.001	1.60E-08	0.005	0.012	0.690
rs67596067	G	A	-0.009	0.001	1.20E-09	-0.019	0.013	0.142
rs6778080	T	C	0.011	0.002	1.30E-12	0.006	0.014	0.650
rs6779302	G	T	-0.009	0.001	1.20E-09	-0.033	0.013	0.008
rs6935954	A	G	0.010	0.001	8.20E-12	-0.007	0.013	0.571
rs6962772	A	G	0.011	0.002	7.80E-09	-0.033	0.017	0.053
rs7039819	G	A	0.009	0.001	5.10E-10	-0.015	0.012	0.221
rs7077678	C	T	0.009	0.001	2.60E-09	0.007	0.013	0.592
rs71367545	G	A	-0.010	0.002	1.40E-09	-0.007	0.016	0.636
rs7155595	A	C	-0.009	0.001	2.50E-09	-0.008	0.013	0.568
rs71627581	G	A	0.013	0.002	1.60E-09	0.027	0.020	0.185
rs72674867	A	T	0.009	0.002	3.80E-08	0.008	0.014	0.587
rs72678864	G	A	0.012	0.002	1.60E-11	0.040	0.017	0.016
rs7297175	T	C	-0.008	0.001	6.60E-09	0.002	0.012	0.874
rs732083	G	A	0.008	0.001	1.50E-08	0.011	0.013	0.412
rs73220544	A	C	-0.011	0.002	1.50E-08	0.012	0.016	0.446
rs7333559	G	A	0.011	0.002	3.20E-10	0.019	0.015	0.216
rs74086911	G	A	0.015	0.003	2.10E-08	0.017	0.023	0.463
rs7519626	C	T	0.008	0.001	1.20E-08	-0.004	0.013	0.772
rs7528604	G	A	0.010	0.001	5.70E-12	0.004	0.012	0.719
rs7553348	G	A	0.010	0.001	5.20E-12	0.014	0.012	0.248

rs7569203	A	C	-0.011	0.002	7.40E-13	0.016	0.013	0.223
rs75742406	G	A	0.010	0.002	1.30E-09	-0.005	0.014	0.721
rs7766610	C	A	0.013	0.002	2.20E-12	-0.022	0.016	0.155
rs7807019	A	G	-0.010	0.001	6.70E-14	0.001	0.012	0.965
rs8042134	T	G	-0.010	0.001	1.30E-12	-0.003	0.013	0.796
rs8042849	C	T	0.019	0.001	1.80E-39	0.001	0.013	0.949
rs812887	A	G	0.008	0.001	5.00E-09	-0.018	0.012	0.140
rs860326	C	T	0.008	0.001	2.70E-09	0.001	0.012	0.968
rs8614	C	A	-0.011	0.002	1.80E-10	0.016	0.016	0.321
rs889398	C	T	0.009	0.001	6.30E-11	0.028	0.012	0.027
rs9435340	T	A	0.008	0.001	1.20E-08	-0.013	0.013	0.317
rs9842947	C	T	-0.009	0.001	3.10E-09	-0.016	0.013	0.233
rs986391	G	A	0.011	0.001	9.40E-15	0.033	0.013	0.010
rs9919670	G	A	-0.015	0.001	7.60E-27	0.007	0.012	0.567

Supplementary Table S11. Genetic instruments (93-SNP - P<5.0x10^{-8}) for alcohol consumption (drinks per week). <u>Abbreviations</u>: SNP, single nucleotide polymorphism; EA, effect allele; OA, other allele; Alc, alcohol consumption; se, standard error.

SNP	EA	OA	beta. Alc	se. Alc	pval. Alc	beta. Cataract	se. Cataract	pval. Cataract
rs1004787	A	G	0.017	0.002	1.13E-22	-0.006	0.012	0.619
rs1011392	G	A	-0.010	0.002	1.78E-08	0.004	0.013	0.728
rs10236149	G	A	-0.016	0.003	4.40E-10	0.046	0.019	0.013
rs10276148	A	G	0.012	0.002	1.19E-11	-0.003	0.012	0.824
rs10743083	G	A	-0.015	0.002	1.64E-10	0.018	0.016	0.271
rs10753661	A	G	-0.011	0.002	1.25E-09	-0.036	0.013	0.007
rs10956823	T	G	0.012	0.002	8.38E-09	-0.019	0.015	0.187
rs11039216	T	C	0.016	0.002	3.76E-19	0.005	0.012	0.709
rs11075711	T	C	-0.013	0.002	1.31E-08	0.008	0.016	0.624
rs111203819	G	T	0.010	0.002	2.41E-08	0.000	0.012	0.985
rs11238438	C	G	0.010	0.002	3.73E-08	-0.012	0.012	0.339
rs11607622	T	C	0.015	0.003	2.22E-08	-0.011	0.020	0.564
rs11692435	A	G	0.019	0.003	7.20E-11	0.028	0.024	0.256
rs11860773	C	T	-0.016	0.002	1.04E-12	0.000	0.016	0.999
rs11940694	G	A	0.028	0.002	9.51E-56	-0.014	0.012	0.255
rs11943397	C	T	0.012	0.002	1.23E-11	0.019	0.013	0.142
rs12044012	A	G	-0.012	0.002	1.34E-09	0.040	0.013	0.002
rs12121630	A	G	-0.014	0.002	1.59E-08	-0.005	0.017	0.773
rs1229984	C	T	0.193	0.005	1.00E-200	-0.012	0.028	0.658
rs1260326	C	T	0.025	0.002	3.16E-46	-0.009	0.012	0.449
rs12646808	C	T	-0.011	0.002	2.47E-09	0.012	0.014	0.394
rs13024996	A	C	-0.014	0.002	5.18E-14	0.006	0.013	0.614
rs13107325	T	C	-0.039	0.004	2.86E-28	0.037	0.023	0.114
rs13236841	G	A	-0.014	0.002	1.92E-12	-0.025	0.014	0.072
rs13288470	T	A	-0.016	0.003	4.05E-09	-0.031	0.019	0.101
rs13332432	G	C	0.013	0.002	1.64E-11	0.003	0.014	0.830
rs147711594	T	G	-0.030	0.005	2.91E-08	-0.054	0.039	0.171
rs153106	C	T	-0.016	0.002	9.38E-19	-0.019	0.013	0.120
rs1558902	A	T	-0.012	0.002	6.42E-11	0.001	0.012	0.958
rs16854020	A	G	0.019	0.003	6.28E-13	0.023	0.018	0.203
rs1838420	T	G	0.010	0.002	1.53E-08	-0.006	0.012	0.602
rs1906252	A	C	0.010	0.002	2.05E-08	0.020	0.012	0.106
rs1942964	G	T	-0.011	0.002	3.89E-09	0.003	0.012	0.822

rs1971157	C	G	0.010	0.002	2.74E-08	0.010	0.013	0.456
rs2049045	C	G	-0.015	0.002	2.24E-11	0.025	0.016	0.113
rs2079106	G	C	0.010	0.002	3.38E-08	0.013	0.013	0.310
rs2087975	G	A	-0.011	0.002	2.02E-09	0.024	0.013	0.053
rs2093186	T	C	-0.012	0.002	1.01E-09	0.014	0.014	0.322
rs2310752	A	G	-0.010	0.002	2.57E-08	-0.007	0.012	0.551
rs2424645	G	A	-0.012	0.002	1.15E-10	0.015	0.013	0.248
rs2533126	A	G	0.013	0.002	1.98E-14	-0.005	0.012	0.661
rs28601761	G	C	0.011	0.002	1.57E-10	0.001	0.013	0.911
rs28616142	T	C	0.011	0.002	2.99E-10	0.005	0.012	0.713
rs28680958	A	G	-0.014	0.002	1.08E-10	-0.021	0.015	0.150
rs28694391	C	T	-0.013	0.002	3.81E-09	-0.008	0.016	0.592
rs28732378	G	A	-0.020	0.002	1.55E-23	0.007	0.014	0.604
rs28929474	T	C	-0.049	0.006	3.28E-14	0.149	0.045	0.001
rs322773	G	A	0.010	0.002	1.48E-08	0.011	0.012	0.367
rs34121753	G	A	0.011	0.002	1.53E-09	-0.018	0.013	0.169
rs34484751	C	A	0.031	0.005	7.86E-10	0.008	0.042	0.853
rs34704785	T	C	-0.010	0.002	4.52E-09	-0.009	0.014	0.515
rs35011311	T	G	-0.011	0.002	8.95E-09	-0.018	0.014	0.202
rs35807116	T	C	0.011	0.002	2.65E-09	0.014	0.013	0.263
rs3768650	G	A	-0.011	0.002	1.67E-08	0.012	0.013	0.373
rs3809162	G	A	0.010	0.002	6.84E-09	-0.026	0.013	0.043
rs4337071	T	C	-0.015	0.002	5.59E-17	0.008	0.013	0.506
rs4481304	A	G	-0.011	0.002	1.25E-09	-0.007	0.012	0.595
rs4743005	A	G	-0.014	0.002	5.57E-10	0.015	0.016	0.337
rs4761961	C	A	-0.011	0.002	5.19E-10	0.000	0.012	0.990
rs4890444	G	C	0.011	0.002	1.89E-09	-0.004	0.013	0.732
rs4916723	C	A	-0.011	0.002	2.74E-10	-0.018	0.012	0.143
rs530916	G	A	0.011	0.002	4.22E-10	-0.011	0.012	0.372
rs55872084	T	G	0.012	0.002	4.68E-09	0.009	0.014	0.522
rs55932213	G	A	0.013	0.002	1.61E-10	0.003	0.014	0.849
rs55987845	T	C	-0.010	0.002	3.41E-08	0.018	0.012	0.140
rs56115085	T	C	-0.013	0.002	4.14E-08	-0.011	0.018	0.555
rs56353702	G	A	-0.014	0.003	2.16E-08	0.006	0.017	0.704
rs60026303	G	A	0.012	0.002	3.58E-08	-0.026	0.015	0.092
rs61873510	T	G	-0.011	0.002	1.17E-08	0.009	0.014	0.485
rs61934664	A	G	-0.012	0.002	8.96E-12	0.004	0.012	0.741

rs62305763	T	C	0.030	0.003	1.15E-28	-0.006	0.021	0.790
rs62641967	G	T	-0.026	0.002	1.74E-30	0.053	0.015	0.000
rs6531148	C	T	-0.012	0.002	7.58E-09	-0.011	0.014	0.449
rs6584893	C	A	-0.011	0.002	3.17E-08	-0.019	0.014	0.184
rs6698883	T	C	-0.016	0.003	1.14E-08	0.014	0.021	0.526
rs6739804	C	T	-0.014	0.002	2.85E-13	-0.011	0.013	0.374
rs6787172	G	T	-0.011	0.002	7.49E-10	-0.004	0.012	0.738
rs68084872	A	G	-0.011	0.002	6.40E-09	-0.016	0.014	0.232
rs6887908	C	A	-0.010	0.002	4.47E-08	-0.003	0.012	0.804
rs6899302	C	T	-0.010	0.002	1.70E-09	0.026	0.013	0.046
rs6962879	G	C	0.010	0.002	1.89E-08	-0.004	0.013	0.725
rs7162115	T	G	0.010	0.002	2.65E-08	-0.012	0.012	0.352
rs7165264	T	G	-0.011	0.002	1.78E-09	-0.017	0.013	0.179
rs72770409	T	C	-0.024	0.004	3.16E-09	-0.003	0.026	0.908
rs75199129	T	A	-0.028	0.004	8.23E-12	-0.023	0.031	0.451
rs7588444	C	T	0.013	0.002	1.76E-09	-0.003	0.015	0.868
rs79616692	C	G	0.020	0.003	3.54E-12	-0.014	0.020	0.486
rs800578	C	T	0.011	0.002	4.51E-08	0.017	0.015	0.255
rs8020892	A	G	-0.013	0.002	2.06E-12	-0.002	0.014	0.903
rs823099	A	C	0.011	0.002	2.31E-09	-0.001	0.012	0.920
rs828867	A	G	0.010	0.002	3.44E-09	-0.012	0.012	0.340
rs838145	A	G	-0.016	0.002	5.62E-21	-0.013	0.013	0.326

Supplementary Table S12. Mendelian randomization leave-one-out analysis for POAG suggesting evidence of causality with cataract. MR estimates using the inverse variance–weighted (IVW) method are reported.

SNP	OR	LCL	UCL	p
rs10248136	1.040	1.006	1.076	0.020
rs10448285	1.040	1.006	1.075	0.022
rs10517281	1.041	1.007	1.076	0.018
rs10800155	1.036	1.001	1.071	0.042
rs109467	1.042	1.008	1.077	0.015
rs1139795	1.043	1.010	1.078	0.011
rs113985657	1.043	1.010	1.077	0.011
rs114367221	1.041	1.007	1.076	0.018
rs12208086	1.041	1.007	1.076	0.018
rs12540035	1.040	1.006	1.076	0.020
rs1577488	1.040	1.006	1.075	0.021
rs1649068	1.037	1.004	1.072	0.029
rs17527016	1.044	1.011	1.078	0.009
rs2027312	1.039	1.005	1.074	0.022
rs2278565	1.042	1.008	1.077	0.015
rs2472494	1.034	1.000	1.069	0.050
rs2514879	1.040	1.006	1.075	0.021
rs257336	1.043	1.010	1.078	0.010
rs2579998	1.042	1.008	1.078	0.015
rs2667477	1.045	1.013	1.079	0.006
rs2735114	1.042	1.009	1.077	0.014
rs28497695	1.038	1.004	1.072	0.026
rs33912345	1.051	1.019	1.085	0.002
rs3825942	1.040	1.006	1.075	0.020
rs41543317	1.041	1.007	1.076	0.018
rs4577906	1.042	1.008	1.077	0.014
rs4612174	1.040	1.006	1.076	0.021
rs4653159	1.040	1.006	1.075	0.022
rs4775427	1.042	1.008	1.077	0.015
rs55882252	1.041	1.006	1.076	0.020
rs56233426	1.039	1.005	1.075	0.023
rs58073046	1.040	1.005	1.075	0.023
rs6117318	1.041	1.007	1.076	0.018
rs62283811	1.041	1.007	1.076	0.018

rs6462143	1.042	1.008	1.077	0.015
rs6475604	1.022	0.990	1.055	0.185
rs6490697	1.044	1.010	1.078	0.010
rs6602453	1.040	1.006	1.075	0.022
rs6845653	1.038	1.004	1.074	0.030
rs686768	1.038	1.005	1.072	0.026
rs7137828	1.039	1.005	1.074	0.024
rs7739648	1.041	1.007	1.077	0.018
rs9544017	1.040	1.006	1.075	0.021
rs9819278	1.039	1.005	1.074	0.024
rs9913911	1.041	1.006	1.076	0.020
rs993471	1.042	1.009	1.077	0.014

Supplementary Table S13. Mendelian randomization leave-one-out analysis for mean spherical equivalent RE suggesting evidence of causality with cataract. MR estimates using the inverse variance–weighted (IVW) method are reported.

SNP	OR	LCL	UCL	p
rs1002191	0.916	0.895	0.938	5.63E-13
rs10025605	0.916	0.895	0.938	5.15E-13
rs10204657	0.916	0.895	0.938	4.41E-13
rs10220706	0.917	0.896	0.939	1.01E-12
rs1055356	0.916	0.895	0.939	6.30E-13
rs10824515	0.915	0.894	0.937	2.16E-13
rs10839545	0.916	0.895	0.938	5.26E-13
rs10853030	0.917	0.896	0.939	9.57E-13
rs10890821	0.916	0.895	0.938	4.11E-13
rs10917958	0.918	0.896	0.940	1.39E-12
rs1106107	0.917	0.896	0.939	1.17E-12
rs11078724	0.917	0.895	0.939	7.85E-13
rs11078918	0.917	0.896	0.939	9.98E-13
rs11083244	0.917	0.895	0.939	8.45E-13
rs11127261	0.919	0.897	0.940	1.24E-12
rs11130365	0.917	0.895	0.939	7.70E-13
rs11145746	0.917	0.895	0.939	7.77E-13
rs1118543	0.918	0.896	0.940	1.27E-12
rs11186433	0.917	0.895	0.939	6.23E-13
rs11602008	0.917	0.895	0.939	1.51E-12
rs11614577	0.917	0.895	0.939	8.28E-13
rs11751433	0.916	0.895	0.938	3.51E-13
rs11909473	0.917	0.896	0.939	9.38E-13
rs12186577	0.916	0.895	0.938	5.49E-13
rs12193446	0.915	0.893	0.938	1.00E-12
rs12308700	0.917	0.895	0.939	6.65E-13
rs12440787	0.918	0.896	0.940	1.05E-12
rs12511880	0.916	0.895	0.938	6.32E-13
rs1254701	0.917	0.895	0.939	7.48E-13
rs12568187	0.917	0.895	0.939	7.32E-13
rs12599282	0.916	0.894	0.937	2.20E-13
rs12615720	0.919	0.897	0.941	2.35E-12
rs12853508	0.916	0.895	0.938	4.61E-13
rs13107325	0.916	0.895	0.938	2.43E-13

rs1323971	0.917	0.895	0.939	8.43E-13
rs13280928	0.916	0.895	0.938	2.74E-13
rs1340044	0.917	0.896	0.939	1.08E-12
rs1367023	0.917	0.896	0.940	1.20E-12
rs1369821	0.917	0.896	0.939	1.03E-12
rs1408345	0.917	0.895	0.939	7.10E-13
rs1420667	0.916	0.895	0.938	3.64E-13
rs1427750	0.918	0.896	0.940	1.23E-12
rs147268199	0.916	0.895	0.938	6.07E-13
rs147792504	0.917	0.896	0.939	9.52E-13
rs1483208	0.916	0.895	0.938	5.53E-13
rs1487441	0.918	0.896	0.940	1.21E-12
rs149850621	0.918	0.896	0.940	1.42E-12
rs1550094	0.916	0.894	0.938	7.12E-13
rs1550870	0.917	0.895	0.939	7.44E-13
rs1571590	0.916	0.895	0.938	5.59E-13
rs1659563	0.917	0.895	0.939	7.80E-13
rs17048756	0.916	0.895	0.938	6.04E-13
rs17283419	0.917	0.896	0.939	1.01E-12
rs17497118	0.917	0.896	0.939	1.02E-12
rs17499741	0.918	0.896	0.940	1.44E-12
rs17855844	0.917	0.896	0.939	9.72E-13
rs1858001	0.917	0.895	0.939	1.04E-12
rs196051	0.916	0.894	0.938	4.04E-13
rs1963456	0.917	0.895	0.939	9.88E-13
rs198442	0.917	0.895	0.939	7.90E-13
rs201224	0.916	0.895	0.938	5.21E-13
rs2046411	0.916	0.895	0.938	2.70E-13
rs2137277	0.916	0.894	0.938	3.66E-13
rs2155413	0.918	0.896	0.940	1.43E-12
rs2160729	0.917	0.896	0.939	1.12E-12
rs2229741	0.917	0.896	0.939	1.06E-12
rs2278161	0.917	0.895	0.939	7.00E-13
rs2281827	0.915	0.894	0.937	9.22E-14
rs2326838	0.916	0.895	0.939	6.45E-13
rs2329144	0.918	0.896	0.940	1.04E-12
rs2745939	0.917	0.896	0.939	1.01E-12

rs2808510	0.916	0.894	0.938	3.53E-13
rs2855530	0.919	0.898	0.941	2.23E-12
rs2856250	0.917	0.896	0.939	1.05E-12
rs2908972	0.916	0.895	0.938	7.11E-13
rs2965185	0.917	0.896	0.939	9.65E-13
rs297588	0.918	0.897	0.940	1.16E-12
rs3138142	0.917	0.895	0.939	1.58E-12
rs34695788	0.917	0.896	0.939	1.06E-12
rs35033460	0.917	0.896	0.939	9.21E-13
rs36024104	0.917	0.896	0.939	1.13E-12
rs36212732	0.918	0.896	0.940	1.27E-12
rs3778987	0.917	0.895	0.939	8.37E-13
rs3812112	0.916	0.895	0.938	5.05E-13
rs3819878	0.917	0.895	0.939	6.71E-13
rs4262652	0.917	0.895	0.939	8.38E-13
rs4278108	0.918	0.897	0.940	1.02E-12
rs429358	0.919	0.899	0.940	2.10E-13
rs442439	0.917	0.896	0.939	1.10E-12
rs4517452	0.917	0.896	0.940	1.54E-12
rs4766878	0.917	0.896	0.939	9.74E-13
rs4792448	0.917	0.895	0.939	7.01E-13
rs4793501	0.917	0.896	0.940	1.18E-12
rs4856278	0.917	0.895	0.939	8.54E-13
rs4858582	0.916	0.895	0.938	4.34E-13
rs500359	0.916	0.895	0.938	5.14E-13
rs501057	0.918	0.896	0.940	1.02E-12
rs524952	0.914	0.892	0.936	2.48E-13
rs55728756	0.916	0.895	0.938	4.20E-13
rs55754534	0.917	0.895	0.939	7.50E-13
rs55899248	0.918	0.896	0.940	1.44E-12
rs55978930	0.917	0.896	0.939	1.20E-12
rs56158821	0.917	0.895	0.939	7.81E-13
rs579728	0.917	0.896	0.939	1.01E-12
rs6054512	0.916	0.895	0.938	4.30E-13
rs6059635	0.918	0.897	0.940	1.18E-12
rs60743220	0.917	0.895	0.939	7.95E-13
rs6121073	0.917	0.896	0.939	1.05E-12

rs61421564	0.917	0.896	0.939	9.30E-13
rs62067167	0.917	0.895	0.939	1.01E-12
rs62169542	0.917	0.895	0.939	1.02E-12
rs62182437	0.917	0.895	0.939	6.98E-13
rs6421566	0.916	0.895	0.938	6.21E-13
rs654169	0.917	0.896	0.939	9.79E-13
rs6548183	0.917	0.895	0.939	9.06E-13
rs6680922	0.917	0.896	0.939	1.22E-12
rs67362351	0.918	0.897	0.940	1.28E-12
rs6764769	0.917	0.896	0.939	1.17E-12
rs67745574	0.918	0.896	0.940	1.05E-12
rs6967089	0.915	0.894	0.937	1.65E-13
rs6979354	0.916	0.895	0.938	4.94E-13
rs6980853	0.916	0.895	0.938	5.44E-13
rs6989160	0.917	0.896	0.939	1.07E-12
rs7042950	0.918	0.896	0.940	1.56E-12
rs7140259	0.917	0.896	0.939	1.17E-12
rs7149665	0.917	0.896	0.939	1.06E-12
rs7162310	0.917	0.896	0.940	1.22E-12
rs7188859	0.920	0.899	0.942	6.44E-12
rs72621438	0.919	0.897	0.941	3.45E-12
rs72683465	0.917	0.896	0.939	1.05E-12
rs72772496	0.917	0.896	0.939	9.86E-13
rs72903976	0.916	0.895	0.938	3.15E-13
rs7326825	0.917	0.895	0.939	7.54E-13
rs73294447	0.915	0.894	0.937	1.86E-13
rs7444298	0.917	0.896	0.939	1.00E-12
rs745030	0.916	0.894	0.938	7.10E-13
rs7465621	0.917	0.896	0.939	1.03E-12
rs75012440	0.917	0.895	0.939	8.21E-13
rs75120545	0.916	0.894	0.937	2.28E-13
rs75227249	0.916	0.895	0.938	4.44E-13
rs75601727	0.916	0.895	0.938	5.05E-13
rs7596847	0.916	0.895	0.938	2.70E-13
rs7744813	0.919	0.897	0.941	6.06E-12
rs77740144	0.918	0.896	0.940	1.12E-12
rs7816934	0.917	0.896	0.939	1.06E-12

rs781832	0.917	0.895	0.939	8.00E-13
rs7848891	0.916	0.895	0.939	6.13E-13
rs7849585	0.917	0.896	0.939	9.52E-13
rs78857879	0.918	0.897	0.940	1.21E-12
rs7944541	0.917	0.896	0.940	1.28E-12
rs807037	0.917	0.895	0.939	7.51E-13
rs8086861	0.917	0.895	0.939	7.03E-13
rs8108157	0.917	0.895	0.939	6.22E-13
rs8132840	0.916	0.895	0.938	5.73E-13
rs854087	0.917	0.895	0.939	6.46E-13
rs893363	0.917	0.895	0.939	9.07E-13
rs893819	0.916	0.895	0.938	4.86E-13
rs9038	0.915	0.894	0.936	3.44E-14
rs9395623	0.916	0.895	0.938	5.52E-13
rs943439	0.916	0.895	0.938	5.15E-13
rs9585327	0.916	0.894	0.938	4.94E-13
rs9611597	0.916	0.895	0.938	5.46E-13
rs9824877	0.917	0.896	0.939	1.10E-12
rs9902531	0.916	0.895	0.938	3.99E-13
rs9934586	0.917	0.895	0.939	8.74E-13
rs999951	0.917	0.895	0.939	6.91E-13

Supplementary Table S14. Mendelian randomization leave-one-out analysis for T2D showing a lack of association with cataract. MR estimates using the inverse variance–weighted (IVW) method are reported.

SNP	OR	LCL	UCL	p
rs10184004	0.997	0.963	1.034	0.890
rs10228456	0.999	0.964	1.035	0.955
rs10411648	0.999	0.964	1.036	0.961
rs10830963	1.000	0.965	1.036	0.990
rs10965246	0.999	0.964	1.036	0.958
rs11257655	1.000	0.964	1.036	0.981
rs112674299	1.000	0.965	1.037	0.991
rs11602873	0.998	0.963	1.034	0.897
rs11658063	0.995	0.961	1.030	0.779
rs116782923	0.996	0.962	1.032	0.822
rs11720108	0.997	0.962	1.032	0.848
rs11759026	1.000	0.965	1.037	0.981
rs11763876	1.004	0.970	1.039	0.827
rs1182395	0.997	0.963	1.033	0.883
rs12611068	0.999	0.964	1.035	0.957
rs12967878	0.999	0.964	1.035	0.936
rs13262861	0.999	0.964	1.035	0.947
rs13414381	0.999	0.964	1.036	0.972
rs1359790	0.999	0.964	1.036	0.958
rs1421085	0.999	0.964	1.036	0.965
rs1496653	1.000	0.965	1.036	0.992
rs17036160	1.000	0.965	1.037	0.987
rs17513135	1.001	0.966	1.037	0.969
rs1800961	1.000	0.965	1.036	0.988
rs2102278	1.000	0.965	1.036	0.993
rs2258238	0.998	0.963	1.034	0.904
rs2278524	0.999	0.964	1.035	0.960
rs243018	0.999	0.964	1.035	0.955
rs2479038	0.999	0.964	1.036	0.972
rs2510078	0.998	0.963	1.034	0.925
rs2796441	0.999	0.964	1.036	0.973
rs28678152	1.002	0.967	1.037	0.933
rs2972144	1.002	0.967	1.038	0.931
rs34715063	0.998	0.963	1.034	0.892

rs34744311	1.002	0.966	1.038	0.933
rs348330	0.997	0.963	1.033	0.884
rs34872471	1.017	0.979	1.058	0.385
rs374722	1.000	0.965	1.036	0.981
rs3802177	0.998	0.963	1.035	0.930
rs465002	0.998	0.963	1.034	0.904
rs4689394	1.001	0.966	1.037	0.970
rs4729854	1.000	0.965	1.036	0.995
rs4886876	0.999	0.964	1.035	0.958
rs5215	1.000	0.965	1.037	0.991
rs55891333	0.999	0.964	1.036	0.969
rs614730	0.997	0.963	1.033	0.878
rs6446298	0.999	0.964	1.036	0.977
rs6777684	1.000	0.965	1.036	0.990
rs6885132	0.997	0.963	1.033	0.882
rs6905775	1.000	0.965	1.036	0.984
rs703972	0.997	0.962	1.033	0.856
rs7125213	1.003	0.969	1.039	0.858
rs72631105	0.999	0.964	1.036	0.973
rs7274168	0.999	0.964	1.035	0.960
rs72802365	0.996	0.961	1.032	0.829
rs72892910	1.000	0.965	1.036	0.999
rs7633675	1.002	0.967	1.039	0.898
rs76895963	0.994	0.959	1.030	0.734
rs7766070	0.999	0.963	1.035	0.939
rs780094	0.998	0.963	1.034	0.892
rs7807512	0.998	0.963	1.035	0.929
rs79687284	1.000	0.965	1.036	0.985
rs7988007	0.998	0.963	1.034	0.910
rs849135	1.000	0.965	1.037	0.984
rs896854	0.994	0.960	1.029	0.729
rs9379084	0.996	0.961	1.031	0.812

Supplementary Table S15. Mendelian randomization leave-one-out analysis for SBP showing a lack of association with cataract. MR estimates using the inverse variance–weighted (IVW) method are reported.

SNP	OR	LCL	UCL	p
rs10219559	1.002	0.990	1.014	0.749
rs11105352	1.004	0.991	1.016	0.568
rs11191548	1.001	0.989	1.013	0.887
rs12509595	1.003	0.991	1.015	0.639
rs12712886	1.002	0.990	1.015	0.720
rs1275984	1.002	0.989	1.015	0.771
rs13021222	1.001	0.989	1.013	0.844
rs13107325	1.002	0.990	1.015	0.708
rs2307032	1.006	0.996	1.016	0.268
rs2392929	1.002	0.990	1.015	0.698
rs4688508	1.001	0.989	1.013	0.863
rs486023	1.002	0.990	1.014	0.749
rs55892892	1.005	0.994	1.016	0.376
rs6108787	1.002	0.990	1.015	0.719
rs6787345	1.003	0.991	1.015	0.662
rs7111257	1.000	0.989	1.012	0.961
rs7115331	1.004	0.993	1.016	0.476
rs7497418	1.001	0.989	1.013	0.847
rs7700842	1.003	0.991	1.015	0.664
rs8105753	1.003	0.991	1.015	0.662

Supplementary Table S16. Mendelian randomization leave-one-out analysis for DBP showing a lack of association with cataract. MR estimates using the inverse variance–weighted (IVW) method are reported.

SNP	OR	LCL	UCL	p
rs10427021	1.008	0.992	1.024	0.348
rs10747570	1.006	0.990	1.022	0.453
rs12509595	1.008	0.992	1.025	0.318
rs12654806	1.005	0.990	1.021	0.522
rs13021222	1.006	0.990	1.022	0.466
rs13107325	1.007	0.991	1.024	0.386
rs142570686	1.005	0.989	1.020	0.567
rs150446556	1.009	0.993	1.025	0.271
rs1731249	1.007	0.990	1.024	0.405
rs17637472	1.004	0.989	1.018	0.629
rs17791208	1.009	0.994	1.025	0.250
rs258317	1.010	0.994	1.025	0.217
rs486023	1.007	0.991	1.023	0.413
rs60228621	1.007	0.991	1.023	0.399
rs6429422	1.007	0.991	1.024	0.368
rs653178	1.011	0.996	1.026	0.160
rs6739828	1.007	0.991	1.023	0.409
rs6787345	1.008	0.992	1.024	0.345
rs7432737	1.009	0.993	1.025	0.257
rs77098653	1.005	0.990	1.020	0.526

Supplementary Table S17. Mendelian randomization leave-one-out analysis for BMI showing a lack of association with cataract. MR estimates using the inverse variance–weighted (IVW) method are reported.

SNP	OR	LCL	UCL	p
rs10063334	0.941	0.865	1.023	0.152
rs10132280	0.949	0.874	1.032	0.222
rs10182181	0.949	0.872	1.032	0.221
rs10192119	0.944	0.868	1.026	0.174
rs10460960	0.944	0.868	1.026	0.174
rs10499694	0.941	0.866	1.023	0.156
rs10733682	0.945	0.869	1.028	0.188
rs10742752	0.943	0.867	1.025	0.166
rs1074657	0.943	0.868	1.026	0.173
rs10840100	0.943	0.867	1.026	0.173
rs10842239	0.942	0.866	1.024	0.160
rs10866869	0.942	0.866	1.024	0.158
rs10883521	0.944	0.868	1.027	0.179
rs10909880	0.944	0.868	1.026	0.175
rs10920678	0.943	0.867	1.025	0.169
rs10929925	0.943	0.867	1.026	0.171
rs10938397	0.944	0.868	1.028	0.184
rs10968576	0.944	0.868	1.027	0.182
rs11057405	0.942	0.866	1.024	0.162
rs1106908	0.941	0.865	1.022	0.151
rs11074452	0.940	0.865	1.021	0.141
rs11081818	0.942	0.866	1.024	0.158
rs11165643	0.939	0.864	1.020	0.139
rs11170468	0.946	0.870	1.029	0.193
rs1126313	0.947	0.870	1.029	0.200
rs11611246	0.942	0.867	1.025	0.164
rs11655589	0.943	0.867	1.025	0.168
rs1167827	0.939	0.864	1.021	0.141
rs11709077	0.942	0.866	1.024	0.158
rs11727676	0.944	0.868	1.026	0.175
rs118067556	0.946	0.870	1.028	0.190
rs11851122	0.939	0.864	1.020	0.138
rs12220375	0.947	0.871	1.030	0.202
rs12286929	0.939	0.864	1.021	0.141

rs12352785	0.946	0.870	1.028	0.193
rs12429545	0.948	0.872	1.031	0.213
rs12887636	0.945	0.870	1.028	0.190
rs12939549	0.942	0.866	1.024	0.160
rs13021737	0.940	0.864	1.024	0.156
rs13107325	0.944	0.868	1.027	0.183
rs13191362	0.942	0.867	1.025	0.165
rs1396141	0.947	0.871	1.029	0.200
rs1421085	0.957	0.877	1.045	0.327
rs1436351	0.943	0.867	1.025	0.167
rs1439620	0.944	0.868	1.027	0.181
rs1441264	0.942	0.867	1.025	0.164
rs144839874	0.942	0.867	1.025	0.165
rs1561277	0.940	0.865	1.022	0.147
rs1561589	0.945	0.869	1.028	0.189
rs1666132	0.944	0.868	1.027	0.180
rs17016673	0.944	0.868	1.026	0.177
rs17024393	0.939	0.865	1.021	0.140
rs17066856	0.947	0.871	1.030	0.205
rs17203016	0.945	0.869	1.027	0.183
rs17405819	0.944	0.868	1.026	0.175
rs17630235	0.943	0.867	1.026	0.172
rs17759796	0.947	0.871	1.029	0.200
rs1819844	0.944	0.868	1.026	0.177
rs188275	0.945	0.869	1.028	0.188
rs1928295	0.945	0.869	1.028	0.185
rs197374	0.946	0.870	1.029	0.195
rs2062332	0.945	0.869	1.028	0.187
rs2112347	0.945	0.869	1.028	0.190
rs2145272	0.946	0.870	1.028	0.190
rs215607	0.939	0.864	1.020	0.136
rs2196618	0.944	0.868	1.026	0.175
rs2216931	0.942	0.866	1.024	0.162
rs2245368	0.944	0.868	1.026	0.176
rs2270204	0.941	0.866	1.023	0.153
rs2287019	0.946	0.870	1.029	0.195
rs2365389	0.943	0.867	1.026	0.173

rs2372716	0.945	0.869	1.027	0.182
rs2481665	0.944	0.868	1.026	0.175
rs2528531	0.944	0.868	1.026	0.175
rs2759315	0.944	0.868	1.027	0.178
rs2820311	0.948	0.871	1.031	0.209
rs2836754	0.943	0.868	1.026	0.173
rs2862996	0.944	0.868	1.027	0.181
rs2890652	0.949	0.873	1.031	0.216
rs29941	0.943	0.867	1.025	0.167
rs3026101	0.942	0.867	1.025	0.164
rs3101336	0.948	0.872	1.031	0.216
rs3127553	0.943	0.867	1.026	0.173
rs34045288	0.948	0.872	1.030	0.209
rs34811474	0.944	0.868	1.026	0.174
rs34976806	0.946	0.870	1.028	0.192
rs3764400	0.944	0.868	1.026	0.176
rs3817334	0.954	0.879	1.036	0.262
rs4130548	0.945	0.869	1.028	0.188
rs4671328	0.945	0.869	1.028	0.188
rs4715213	0.939	0.863	1.021	0.138
rs4740619	0.947	0.871	1.029	0.201
rs4757142	0.945	0.869	1.027	0.184
rs4776970	0.947	0.871	1.030	0.205
rs4788099	0.937	0.862	1.019	0.129
rs4820408	0.943	0.867	1.026	0.171
rs4833079	0.947	0.872	1.030	0.204
rs487152	0.946	0.870	1.029	0.194
rs492400	0.944	0.868	1.026	0.176
rs4986044	0.944	0.868	1.027	0.179
rs543874	0.945	0.868	1.028	0.187
rs592483	0.943	0.867	1.025	0.170
rs6091540	0.946	0.870	1.029	0.195
rs6142096	0.939	0.865	1.020	0.138
rs6265	0.949	0.873	1.032	0.224
rs6471932	0.942	0.867	1.024	0.162
rs6477694	0.942	0.867	1.024	0.162
rs6545714	0.942	0.867	1.025	0.164

rs6567160	0.954	0.877	1.038	0.278
rs6569648	0.948	0.873	1.030	0.210
rs6676190	0.944	0.868	1.027	0.178
rs6713510	0.948	0.872	1.030	0.205
rs6804842	0.946	0.870	1.028	0.190
rs6864049	0.944	0.868	1.027	0.178
rs6870983	0.938	0.864	1.019	0.129
rs687339	0.942	0.866	1.024	0.161
rs7138803	0.946	0.870	1.029	0.199
rs7143963	0.948	0.872	1.030	0.209
rs7144011	0.944	0.868	1.027	0.178
rs7161194	0.939	0.864	1.020	0.136
rs7193901	0.945	0.869	1.028	0.185
rs7239883	0.940	0.865	1.022	0.148
rs749671	0.947	0.872	1.030	0.205
rs756717	0.945	0.869	1.027	0.184
rs7580766	0.946	0.870	1.028	0.192
rs758747	0.940	0.865	1.021	0.143
rs7599312	0.948	0.872	1.030	0.209
rs7613875	0.942	0.866	1.025	0.164
rs7629375	0.945	0.869	1.028	0.186
rs7647305	0.946	0.869	1.029	0.195
rs77165542	0.949	0.873	1.032	0.219
rs7730898	0.945	0.870	1.028	0.189
rs7899106	0.947	0.871	1.029	0.200
rs7903146	0.937	0.863	1.018	0.122
rs80255756	0.947	0.871	1.030	0.203
rs8036171	0.946	0.870	1.029	0.193
rs815610	0.944	0.868	1.027	0.179
rs879620	0.940	0.864	1.022	0.145
rs889398	0.954	0.882	1.033	0.250
rs891389	0.945	0.869	1.028	0.189
rs901630	0.944	0.868	1.027	0.178
rs935166	0.945	0.869	1.028	0.189
rs9400239	0.938	0.864	1.019	0.131
rs9462027	0.945	0.869	1.028	0.186
rs946711	0.945	0.869	1.028	0.190

rs947612	0.944	0.868	1.027	0.181
rs9512699	0.942	0.867	1.025	0.166
rs9513140	0.951	0.876	1.033	0.235
rs9527895	0.941	0.866	1.023	0.155
rs9563576	0.946	0.870	1.029	0.197
rs972540	0.944	0.868	1.026	0.177
rs977747	0.947	0.871	1.029	0.199
rs9856151	0.944	0.869	1.027	0.182

Supplementary Table S18. Mendelian randomization leave-one-out analysis for cigarette smoking initiation showing a lack of association with cataract. MR estimates using the inverse variance–weighted (IVW) method are reported.

SNP	OR	LCL	UCL	p
rs10001365	1.145	0.979	1.339	0.091
rs1004787	1.155	0.988	1.352	0.071
rs10062607	1.152	0.985	1.347	0.077
rs10119117	1.151	0.984	1.346	0.078
rs10121930	1.158	0.992	1.353	0.064
rs10233018	1.149	0.982	1.344	0.083
rs1025910	1.156	0.989	1.352	0.068
rs10279261	1.150	0.983	1.344	0.081
rs10444314	1.150	0.984	1.345	0.080
rs10458563	1.141	0.976	1.334	0.098
rs1050847	1.156	0.989	1.351	0.069
rs10698713	1.150	0.984	1.345	0.079
rs10745324	1.163	0.997	1.357	0.055
rs10753630	1.144	0.979	1.337	0.091
rs10786721	1.156	0.988	1.352	0.070
rs1084445	1.155	0.988	1.350	0.070
rs10914684	1.166	1.000	1.360	0.050
rs10927039	1.154	0.987	1.349	0.073
rs10954115	1.156	0.989	1.351	0.068
rs11012726	1.155	0.988	1.351	0.071
rs11078713	1.155	0.988	1.351	0.071
rs1111578	1.144	0.979	1.337	0.091
rs11130381	1.155	0.988	1.350	0.070
rs11162019	1.150	0.984	1.345	0.080
rs11162976	1.149	0.983	1.344	0.081
rs111861749	1.144	0.979	1.337	0.091
rs11210229	1.140	0.975	1.333	0.100
rs11258417	1.151	0.984	1.345	0.079
rs114050142	1.145	0.979	1.338	0.090
rs114900182	1.152	0.985	1.347	0.076
rs1155641	1.151	0.984	1.345	0.079
rs11632439	1.154	0.987	1.349	0.072
rs11656151	1.138	0.974	1.329	0.102
rs11673452	1.144	0.979	1.337	0.091

rs11693702	1.153	0.986	1.349	0.074
rs11695197	1.145	0.980	1.339	0.088
rs11716705	1.156	0.989	1.352	0.068
rs11720703	1.156	0.989	1.351	0.069
rs11742625	1.145	0.979	1.338	0.090
rs11756490	1.151	0.985	1.346	0.077
rs11873164	1.147	0.981	1.341	0.086
rs11926232	1.149	0.983	1.344	0.081
rs12027999	1.158	0.991	1.354	0.065
rs12036050	1.146	0.980	1.340	0.087
rs12079063	1.143	0.978	1.336	0.092
rs12112638	1.140	0.976	1.332	0.099
rs12133063	1.152	0.985	1.347	0.076
rs12209775	1.146	0.980	1.340	0.088
rs12213996	1.151	0.984	1.346	0.078
rs12441907	1.140	0.975	1.332	0.100
rs1246265	1.144	0.979	1.337	0.091
rs12485391	1.155	0.988	1.350	0.070
rs12600466	1.149	0.983	1.344	0.081
rs12632110	1.147	0.981	1.341	0.086
rs12642744	1.146	0.980	1.340	0.087
rs12685816	1.153	0.986	1.348	0.075
rs12714017	1.148	0.982	1.343	0.083
rs1291865	1.147	0.981	1.341	0.086
rs12923427	1.153	0.986	1.349	0.074
rs13009008	1.150	0.983	1.344	0.081
rs13030994	1.146	0.979	1.341	0.091
rs13145728	1.152	0.985	1.347	0.076
rs13162305	1.153	0.986	1.348	0.074
rs13246563	1.151	0.984	1.346	0.079
rs13261725	1.148	0.982	1.343	0.084
rs13301073	1.154	0.987	1.349	0.073
rs1334557	1.148	0.982	1.342	0.084
rs13377168	1.150	0.983	1.345	0.080
rs13398418	1.155	0.988	1.350	0.071
rs134529	1.149	0.982	1.343	0.082
rs1381287	1.160	0.993	1.356	0.062

rs1392446	1.147	0.981	1.341	0.086
rs1435679	1.152	0.986	1.347	0.075
rs143909875	1.151	0.984	1.346	0.078
rs147052174	1.152	0.985	1.347	0.076
rs1485272	1.141	0.977	1.332	0.096
rs1499982	1.156	0.989	1.352	0.069
rs1503211	1.147	0.981	1.341	0.086
rs1559278	1.153	0.986	1.348	0.075
rs1565735	1.152	0.984	1.348	0.078
rs160631	1.145	0.979	1.338	0.089
rs16896199	1.147	0.981	1.340	0.086
rs16975171	1.158	0.991	1.353	0.066
rs17207435	1.152	0.986	1.347	0.075
rs1733756	1.144	0.979	1.338	0.091
rs17432775	1.144	0.979	1.337	0.091
rs17594561	1.153	0.986	1.348	0.074
rs1776631	1.150	0.983	1.345	0.080
rs1820083	1.151	0.984	1.346	0.078
rs1901477	1.157	0.989	1.353	0.068
rs1937443	1.150	0.983	1.345	0.080
rs1945737	1.154	0.987	1.350	0.072
rs1971318	1.146	0.980	1.341	0.087
rs1994247	1.148	0.982	1.343	0.084
rs2022815	1.155	0.988	1.350	0.070
rs2072155	1.145	0.979	1.338	0.090
rs2101364	1.155	0.988	1.350	0.070
rs2135160	1.157	0.990	1.352	0.067
rs2163413	1.149	0.983	1.344	0.081
rs2173019	1.141	0.977	1.334	0.096
rs2237303	1.149	0.983	1.344	0.081
rs2289791	1.153	0.986	1.348	0.074
rs2292239	1.147	0.981	1.341	0.085
rs2313500	1.142	0.977	1.334	0.096
rs2678903	1.151	0.985	1.346	0.078
rs2708630	1.155	0.988	1.350	0.071
rs2711607	1.151	0.985	1.346	0.077
rs2783130	1.147	0.981	1.341	0.085

rs2876586	1.162	0.996	1.356	0.056
rs288181	1.149	0.983	1.344	0.081
rs2939756	1.138	0.975	1.329	0.102
rs3110590	1.159	0.993	1.354	0.062
rs3213876	1.152	0.985	1.347	0.077
rs326341	1.155	0.988	1.351	0.070
rs326387	1.144	0.979	1.338	0.091
rs332827	1.158	0.991	1.352	0.065
rs34367058	1.152	0.985	1.347	0.076
rs34371841	1.141	0.977	1.333	0.096
rs34488670	1.154	0.987	1.350	0.073
rs35891966	1.146	0.981	1.340	0.087
rs3781295	1.151	0.984	1.346	0.078
rs3801289	1.152	0.986	1.348	0.076
rs3814994	1.145	0.980	1.338	0.089
rs3895907	1.143	0.978	1.336	0.094
rs3905125	1.141	0.976	1.332	0.097
rs3934797	1.134	0.972	1.324	0.110
rs39784	1.141	0.977	1.333	0.096
rs4044321	1.135	0.971	1.326	0.111
rs4374330	1.151	0.984	1.346	0.078
rs4401691	1.151	0.984	1.346	0.078
rs4479577	1.146	0.981	1.340	0.086
rs4543592	1.153	0.986	1.349	0.074
rs4571506	1.165	0.997	1.362	0.054
rs4579569	1.153	0.986	1.349	0.074
rs465646	1.165	0.997	1.362	0.055
rs4659805	1.149	0.983	1.344	0.081
rs4751614	1.147	0.981	1.341	0.085
rs4785187	1.152	0.986	1.347	0.075
rs4819027	1.143	0.978	1.336	0.092
rs4837631	1.154	0.987	1.349	0.072
rs4841484	1.153	0.986	1.348	0.074
rs4857114	1.149	0.982	1.343	0.083
rs4865172	1.149	0.982	1.343	0.082
rs4888444	1.143	0.978	1.336	0.093
rs4944844	1.155	0.988	1.350	0.070

rs540356	1.169	1.003	1.363	0.046
rs540860	1.162	0.994	1.357	0.059
rs551739	1.149	0.983	1.343	0.082
rs557544	1.155	0.988	1.350	0.071
rs55921136	1.141	0.977	1.333	0.097
rs55942317	1.153	0.987	1.349	0.073
rs56169608	1.152	0.985	1.347	0.076
rs56348592	1.143	0.978	1.336	0.093
rs56820925	1.150	0.983	1.345	0.080
rs574835	1.142	0.978	1.334	0.094
rs5751239	1.158	0.990	1.353	0.066
rs58400863	1.144	0.979	1.338	0.091
rs60453921	1.146	0.981	1.340	0.087
rs6088618	1.156	0.989	1.351	0.069
rs6141314	1.159	0.992	1.355	0.063
rs61533748	1.150	0.983	1.344	0.081
rs61959481	1.149	0.983	1.344	0.082
rs62107261	1.151	0.985	1.347	0.077
rs62135525	1.147	0.981	1.341	0.086
rs62181771	1.142	0.978	1.335	0.094
rs62250713	1.160	0.991	1.356	0.064
rs62254171	1.150	0.984	1.345	0.080
rs62258903	1.156	0.989	1.351	0.069
rs62266876	1.144	0.979	1.337	0.091
rs62419578	1.147	0.981	1.341	0.085
rs6265	1.164	0.996	1.360	0.056
rs6438208	1.156	0.990	1.351	0.067
rs6464024	1.157	0.990	1.353	0.066
rs6472232	1.160	0.993	1.354	0.061
rs6497840	1.154	0.987	1.349	0.073
rs6598539	1.146	0.980	1.339	0.088
rs66480876	1.150	0.983	1.345	0.080
rs66680800	1.147	0.981	1.341	0.086
rs6705147	1.145	0.980	1.338	0.089
rs6728726	1.158	0.990	1.354	0.066
rs6852117	1.153	0.986	1.349	0.074
rs6959670	1.145	0.979	1.338	0.090

rs6973168	1.147	0.981	1.341	0.086
rs7026534	1.153	0.986	1.348	0.075
rs7092291	1.147	0.981	1.341	0.085
rs7127712	1.162	0.992	1.361	0.063
rs71367545	1.148	0.982	1.342	0.083
rs71491832	1.157	0.990	1.352	0.067
rs71627577	1.144	0.978	1.337	0.092
rs7195043	1.154	0.987	1.349	0.072
rs7205551	1.142	0.977	1.334	0.095
rs7224742	1.145	0.979	1.338	0.090
rs72733235	1.149	0.982	1.343	0.082
rs72789627	1.151	0.984	1.346	0.079
rs72904370	1.150	0.984	1.345	0.080
rs7333559	1.144	0.978	1.337	0.092
rs75177132	1.142	0.978	1.335	0.094
rs7585579	1.150	0.984	1.345	0.080
rs76132272	1.151	0.984	1.345	0.079
rs7629352	1.141	0.977	1.332	0.096
rs77307359	1.152	0.985	1.346	0.077
rs7788527	1.150	0.984	1.345	0.080
rs7804551	1.162	0.995	1.357	0.058
rs78175438	1.152	0.985	1.347	0.077
rs7829715	1.152	0.985	1.347	0.077
rs7893954	1.144	0.979	1.337	0.091
rs79222572	1.142	0.978	1.335	0.093
rs7929518	1.151	0.984	1.346	0.078
rs7947391	1.159	0.993	1.354	0.062
rs7969559	1.149	0.982	1.343	0.082
rs7984311	1.146	0.981	1.340	0.086
rs7986094	1.143	0.978	1.336	0.092
rs8001839	1.148	0.982	1.343	0.083
rs8067305	1.158	0.991	1.353	0.065
rs8069451	1.144	0.978	1.337	0.092
rs846781	1.146	0.980	1.340	0.087
rs853684	1.156	0.989	1.351	0.069
rs888292	1.147	0.981	1.341	0.086
rs911781	1.149	0.983	1.344	0.081

rs9323328	1.147	0.981	1.341	0.086
rs9375371	1.146	0.980	1.340	0.089
rs9402093	1.153	0.986	1.348	0.074
rs9423279	1.146	0.980	1.340	0.088
rs951740	1.146	0.979	1.340	0.089
rs9529052	1.142	0.977	1.335	0.096
rs9538536	1.148	0.982	1.342	0.084
rs9541499	1.142	0.978	1.335	0.093
rs9613472	1.148	0.982	1.343	0.083
rs963354	1.144	0.979	1.338	0.091
rs993700	1.138	0.974	1.329	0.103
rs9987376	1.149	0.982	1.344	0.082

Supplementary Table S19. Mendelian randomization leave-one-out analysis for cigarettes per week showing a lack of association with cataract.

SNP	OR	LCL	UCL	P
rs11076320	1.047	0.888	1.236	0.583
rs112178027	1.067	0.905	1.259	0.440
rs112270518	1.066	0.902	1.261	0.452
rs11663346	1.051	0.890	1.241	0.556
rs11686893	1.045	0.887	1.230	0.602
rs11725618	1.048	0.888	1.237	0.578
rs11921010	1.051	0.890	1.241	0.556
rs11928552	1.051	0.890	1.241	0.559
rs11940430	1.053	0.891	1.245	0.541
rs12660603	1.054	0.892	1.246	0.534
rs13254578	1.060	0.894	1.257	0.500
rs138759397	1.066	0.902	1.258	0.454
rs141147481	1.044	0.887	1.229	0.602
rs1444026	1.051	0.890	1.242	0.554
rs145104523	1.058	0.895	1.251	0.511
rs1657936	1.048	0.888	1.236	0.581
rs17197116	1.072	0.910	1.263	0.406
rs1737894	1.085	0.924	1.274	0.318
rs185771419	1.049	0.889	1.238	0.572
rs2016968	1.066	0.902	1.259	0.455
rs2060220	1.053	0.891	1.244	0.546
rs2072659	1.057	0.894	1.250	0.514
rs215600	1.050	0.888	1.241	0.571
rs244417	1.037	0.883	1.218	0.657
rs2655008	1.053	0.891	1.244	0.546
rs34370696	1.062	0.899	1.255	0.478
rs34406232	1.076	0.911	1.271	0.389
rs3796462	1.056	0.894	1.249	0.522
rs56113850	1.092	0.911	1.309	0.340
rs6078372	1.046	0.887	1.233	0.593
rs6603895	1.068	0.905	1.259	0.437
rs6697255	1.065	0.902	1.257	0.461
rs6699355	1.062	0.899	1.254	0.482
rs6831786	1.044	0.886	1.230	0.607

rs72738704	1.092	0.897	1.330	0.381
rs73229090	1.062	0.898	1.256	0.479
rs7599488	1.060	0.897	1.253	0.493
rs7678019	1.064	0.900	1.257	0.469
rs79250609	1.057	0.894	1.250	0.515
rs7928017	1.056	0.893	1.249	0.521
rs7933830	1.062	0.898	1.255	0.484
rs7944241	1.066	0.902	1.258	0.453
rs8021229	1.060	0.897	1.254	0.494
rs80292109	1.061	0.897	1.254	0.492
rs806255	1.049	0.888	1.239	0.572
rs9522262	1.048	0.889	1.237	0.575
rs9881798	1.037	0.883	1.217	0.661

Supplementary Table S20. Mendelian randomization leave-one-out analysis for lifetime smoking showing a lack of association with cataract. MR estimates using the inverse variance–weighted (IVW) method are reported.

SNP	OR	LCL	UCL	p
rs10052591	1.074	0.833	1.385	0.582
rs10226228	1.056	0.818	1.362	0.678
rs10282292	1.058	0.820	1.365	0.664
rs1050847	1.076	0.835	1.387	0.570
rs10823968	1.068	0.828	1.378	0.611
rs10879871	1.075	0.833	1.386	0.579
rs10918701	1.055	0.819	1.360	0.679
rs10922907	1.057	0.819	1.364	0.668
rs11210229	1.043	0.810	1.344	0.742
rs112282219	1.074	0.832	1.385	0.584
rs11255908	1.062	0.823	1.370	0.643
rs113382419	1.083	0.837	1.400	0.545
rs11768481	1.071	0.830	1.382	0.597
rs11783093	1.062	0.823	1.372	0.642
rs11948770	1.059	0.821	1.366	0.660
rs12202536	1.076	0.835	1.387	0.570
rs1221148	1.070	0.830	1.381	0.601
rs12244388	1.078	0.835	1.392	0.564
rs1246265	1.054	0.818	1.358	0.686
rs12481282	1.068	0.828	1.378	0.613
rs12623702	1.084	0.841	1.396	0.534
rs12708665	1.067	0.827	1.376	0.619
rs12831617	1.079	0.837	1.389	0.559
rs12967855	1.067	0.827	1.376	0.618
rs13009008	1.065	0.825	1.373	0.630
rs13016665	1.056	0.819	1.362	0.672
rs13153393	1.052	0.817	1.356	0.693
rs13296519	1.068	0.828	1.379	0.611
rs136233	1.067	0.827	1.376	0.619
rs147412694	1.065	0.825	1.374	0.629
rs17309874	1.077	0.835	1.388	0.570
rs17553262	1.068	0.828	1.377	0.614
rs17576594	1.059	0.821	1.366	0.661
rs1922018	1.071	0.830	1.382	0.598

rs1931263	1.076	0.835	1.387	0.571
rs1933270	1.049	0.815	1.351	0.709
rs202645	1.088	0.846	1.398	0.512
rs2062882	1.063	0.824	1.371	0.640
rs2254710	1.064	0.825	1.372	0.634
rs2401924	1.053	0.817	1.359	0.690
rs245774	1.050	0.816	1.351	0.704
rs2675638	1.058	0.821	1.365	0.662
rs2678670	1.071	0.830	1.381	0.599
rs2838834	1.043	0.812	1.341	0.740
rs28485305	1.065	0.825	1.374	0.628
rs28635466	1.071	0.830	1.381	0.599
rs2867112	1.077	0.835	1.390	0.568
rs2890772	1.039	0.806	1.340	0.766
rs2894808	1.067	0.827	1.377	0.617
rs317021	1.078	0.837	1.390	0.560
rs326341	1.078	0.836	1.390	0.564
rs329120	1.054	0.817	1.358	0.687
rs34866095	1.069	0.829	1.379	0.606
rs348829	1.052	0.817	1.355	0.695
rs35169606	1.082	0.840	1.393	0.540
rs35175834	1.076	0.833	1.389	0.577
rs359243	1.059	0.821	1.365	0.661
rs369230	1.067	0.827	1.376	0.619
rs3742365	1.078	0.836	1.391	0.562
rs3769949	1.068	0.828	1.378	0.611
rs3811038	1.072	0.831	1.383	0.592
rs3896224	1.090	0.847	1.402	0.503
rs421983	1.105	0.866	1.410	0.423
rs4391802	1.043	0.811	1.341	0.745
rs4473348	1.066	0.826	1.376	0.623
rs4543592	1.071	0.830	1.381	0.598
rs4568549	1.045	0.814	1.343	0.728
rs4571506	1.085	0.843	1.395	0.527
rs4671357	1.059	0.821	1.367	0.658
rs4731925	1.070	0.830	1.380	0.602
rs4814873	1.074	0.833	1.385	0.582

rs4957528	1.073	0.832	1.384	0.587
rs530916	1.075	0.834	1.386	0.576
rs549845	1.064	0.825	1.374	0.632
rs57611503	1.073	0.832	1.383	0.588
rs60952428	1.072	0.831	1.383	0.591
rs6119897	1.081	0.839	1.394	0.546
rs61796681	1.074	0.833	1.384	0.582
rs62098013	1.052	0.817	1.355	0.696
rs62135536	1.053	0.817	1.356	0.690
rs62155874	1.050	0.814	1.354	0.707
rs62175972	1.072	0.831	1.383	0.592
rs624833	1.074	0.833	1.385	0.583
rs6562474	1.072	0.831	1.382	0.595
rs6598539	1.057	0.820	1.363	0.670
rs6692614	1.073	0.832	1.384	0.585
rs6741228	1.062	0.823	1.370	0.642
rs67596067	1.050	0.816	1.353	0.703
rs6778080	1.061	0.822	1.369	0.651
rs6779302	1.037	0.809	1.330	0.772
rs6935954	1.073	0.832	1.385	0.586
rs6962772	1.086	0.845	1.396	0.517
rs7039819	1.080	0.838	1.392	0.550
rs7077678	1.061	0.822	1.368	0.651
rs71367545	1.061	0.823	1.369	0.647
rs7155595	1.060	0.822	1.368	0.652
rs71627581	1.053	0.817	1.356	0.691
rs72674867	1.061	0.822	1.368	0.649
rs72678864	1.038	0.809	1.333	0.768
rs7297175	1.068	0.828	1.378	0.613
rs732083	1.058	0.820	1.364	0.664
rs73220544	1.074	0.833	1.385	0.580
rs7333559	1.053	0.817	1.356	0.692
rs74086911	1.059	0.821	1.365	0.659
rs7519626	1.069	0.829	1.379	0.606
rs7528604	1.062	0.823	1.370	0.644
rs7553348	1.052	0.816	1.356	0.695
rs7569203	1.083	0.840	1.395	0.540

rs75742406	1.070	0.829	1.380	0.602
rs7766610	1.085	0.842	1.397	0.528
rs7807019	1.067	0.827	1.378	0.618
rs8042134	1.063	0.824	1.372	0.637
rs8042849	1.067	0.824	1.381	0.624
rs812887	1.082	0.840	1.393	0.541
rs860326	1.066	0.826	1.375	0.624
rs8614	1.078	0.836	1.389	0.563
rs889398	1.040	0.810	1.336	0.757
rs9435340	1.076	0.835	1.387	0.569
rs9842947	1.054	0.818	1.358	0.686
rs986391	1.031	0.804	1.323	0.810
rs9919670	1.079	0.835	1.394	0.562

Supplementary Table S21. Mendelian randomization leave-one-out analysis for alcohol consumption showing a lack of association with cataract. MR estimates using the inverse variance–weighted (IVW) method are reported.

SNP	OR	LCL	UCL	p
rs1004787	0.860	0.706	1.048	0.136
rs1011392	0.859	0.705	1.046	0.130
rs10236149	0.871	0.718	1.056	0.160
rs10276148	0.858	0.705	1.045	0.129
rs10743083	0.863	0.709	1.051	0.143
rs10753661	0.843	0.696	1.020	0.079
rs10956823	0.864	0.710	1.051	0.143
rs11039216	0.853	0.700	1.040	0.115
rs11075711	0.860	0.706	1.047	0.133
rs111203819	0.857	0.704	1.044	0.125
rs11238438	0.862	0.708	1.049	0.138
rs11607622	0.860	0.706	1.047	0.133
rs11692435	0.852	0.700	1.036	0.108
rs11860773	0.857	0.703	1.044	0.125
rs11940694	0.869	0.712	1.062	0.170
rs11943397	0.848	0.698	1.031	0.098
rs12044012	0.874	0.723	1.057	0.166
rs12121630	0.856	0.703	1.042	0.121
rs1229984	0.816	0.639	1.042	0.103
rs1260326	0.864	0.707	1.054	0.150
rs12646808	0.861	0.708	1.049	0.137
rs13024996	0.860	0.706	1.048	0.134
rs13107325	0.872	0.716	1.062	0.174
rs13236841	0.845	0.696	1.027	0.090
rs13288470	0.849	0.698	1.031	0.098
rs13332432	0.856	0.703	1.042	0.121
rs147711594	0.851	0.700	1.034	0.104
rs153106	0.844	0.694	1.026	0.089

rs1558902	0.857	0.704	1.044	0.126
rs16854020	0.849	0.698	1.032	0.100
rs1838420	0.860	0.706	1.047	0.133
rs1906252	0.849	0.699	1.032	0.100
rs1942964	0.858	0.705	1.045	0.129
rs1971157	0.854	0.702	1.039	0.115
rs2049045	0.867	0.713	1.054	0.151
rs2079106	0.852	0.701	1.037	0.110
rs2087975	0.868	0.714	1.054	0.152
rs2093186	0.862	0.709	1.050	0.140
rs2310752	0.854	0.702	1.040	0.116
rs2424645	0.864	0.710	1.051	0.143
rs2533126	0.860	0.706	1.047	0.133
rs28601761	0.856	0.703	1.043	0.123
rs28616142	0.855	0.702	1.041	0.119
rs28680958	0.848	0.698	1.031	0.099
rs28694391	0.854	0.702	1.040	0.117
rs28732378	0.860	0.706	1.049	0.137
rs28929474	0.880	0.728	1.063	0.185
rs322773	0.853	0.701	1.038	0.111
rs34121753	0.864	0.711	1.051	0.144
rs34484751	0.856	0.703	1.043	0.123
rs34704785	0.854	0.702	1.040	0.116
rs35011311	0.851	0.700	1.034	0.105
rs35807116	0.851	0.700	1.035	0.107
rs3768650	0.862	0.708	1.049	0.138
rs3809162	0.867	0.714	1.053	0.151
rs4337071	0.861	0.707	1.049	0.139
rs4481304	0.854	0.702	1.040	0.116
rs4743005	0.862	0.708	1.050	0.140
rs4761961	0.857	0.704	1.044	0.125
rs4890444	0.859	0.705	1.046	0.130

rs4916723	0.849	0.698	1.032	0.100
rs530916	0.862	0.708	1.049	0.139
rs55872084	0.854	0.701	1.039	0.115
rs55932213	0.856	0.703	1.042	0.122
rs55987845	0.864	0.711	1.051	0.144
rs56115085	0.855	0.702	1.040	0.117
rs56353702	0.859	0.705	1.046	0.131
rs60026303	0.865	0.712	1.052	0.147
rs61873510	0.861	0.707	1.048	0.135
rs61934664	0.859	0.705	1.046	0.130
rs62305763	0.858	0.704	1.046	0.130
rs62641967	0.897	0.743	1.085	0.263
rs6531148	0.853	0.701	1.038	0.113
rs6584893	0.851	0.700	1.034	0.105
rs6698883	0.860	0.707	1.047	0.134
rs6739804	0.851	0.699	1.036	0.107
rs6787172	0.855	0.702	1.041	0.119
rs68084872	0.851	0.700	1.035	0.106
rs6887908	0.856	0.703	1.042	0.122
rs6899302	0.867	0.714	1.053	0.151
rs6962879	0.859	0.705	1.046	0.130
rs7162115	0.862	0.708	1.049	0.138
rs7165264	0.850	0.699	1.033	0.102
rs72770409	0.856	0.703	1.043	0.123
rs75199129	0.853	0.700	1.038	0.112
rs7588444	0.858	0.704	1.045	0.128
rs79616692	0.861	0.707	1.049	0.137
rs800578	0.852	0.700	1.036	0.108
rs8020892	0.856	0.703	1.043	0.122
rs823099	0.858	0.704	1.044	0.127
rs828867	0.862	0.708	1.049	0.139
rs838145	0.848	0.697	1.033	0.101

rs9875033	0.861	0.708	1.048	0.137

Supplementary Table S22. Evaluation of MR associations of POAG with cataract under different MR models

MR method	OR (95% CI)	*P*-value
IVW	0.04 (1.01-.08)	0.018
Weighted median	1.06 (1.02-1.10)	0.003
Weighted mode	1.09 (1.03-1.16)	0.008
MR Egger	1.16 (1.07-1.27)	0.001
MR-PRESSO model	1.03 (1.01-1.07)	0.04

Abbreviations: POAG, primary open-angle glaucoma; N, number of genetic instruments after clumping; MR, Mendelian randomization; IVW, Inverse-variance weighted model; OR, odds ratio; CI, confidence intervals; SNP, single nucleotide polymorphism; IGGC, International Glaucoma Genetics Consortium; UKB. UK Biobank.

Supplementary Table S23. Evaluation of MR associations of mean spherical equivalent RE with cataract under different MR models

Method	OR	LCL	UCL	pval
IVW	1.04	1.007	1.075	0.018
MR Egger	1.164	1.068	1.269	0.001
Weighted median	1.06	1.02	1.102	0.003
Weighted mode	1.092	1.026	1.161	0.008
MR-PRESSO model	1.033	1.002	1.065	0.044

Supplementary Table S24. Evaluation of MR associations of T2D with cataract under different MR models

Method	OR	LCL	UCL	pval
IVW	0.999	0.965	1.035	0.96
MR Egger	0.965	0.890	1.047	0.40
Weighted median	0.971	0.923	1.021	0.24
Weighted mode	0.950	0.890	1.014	0.13
MR-PRESSO model	0.999	0.965	1.035	0.96

Supplementary Table S25. Evaluation of MR associations of SBP with cataract under different MR models

Method	OR	LCL	UCL	pval
IVW	1.002	0.991	1.014	0.68
MR Egger	0.996	0.952	1.043	0.87
Weighted median	1.007	0.993	1.021	0.34
Weighted mode	1.005	0.984	1.027	0.64
MR-PRESSO model	1.002	0.991	1.014	0.68

Supplementary Table S26. Evaluation of MR associations of DBP with cataract under different MR models

Method	OR	LCL	UCL	pval
IVW	1.007	0.992	1.023	0.36
MR Egger	1.006	0.949	1.066	0.84
Weighted median	1.007	0.988	1.027	0.47
Weighted mode	1.003	0.969	1.039	0.85
MR-PRESSO model	1.007	0.992	1.023	0.37

Supplementary Table S27. Evaluation of MR associations of BMI with cataract under different MR models

Method	OR	LCL	UCL	pval
IVW	1.022	0.891	1.173	0.75
MR Egger	0.924	0.661	1.292	0.64
Weighted median	1.002	0.815	1.231	0.99
Weighted mode	0.955	0.753	1.211	0.71
MR-PRESSO model	1.022	0.891	1.173	0.75

Supplementary Table S28. Evaluation of MR associations of smoking initiation with cataract under different MR models

Method	OR	LCL	UCL	pval
IVW	1.15	0.984	1.344	0.079
MR Egger	0.993	0.529	1.863	0.98
Weighted median	1.030	0.828	1.281	0.79
Weighted mode	0.856	0.506	1.450	0.56
MR-PRESSO model	1.150	0.984	1.344	0.08

Supplementary Table S29. Evaluation of MR associations of cigarettes per day with cataract under different MR models

Method	OR	LCL	UCL	pval
IVW	1.058	0.897	1.247	0.51
MR Egger	0.815	0.612	1.085	0.17
Weighted median	0.961	0.748	1.236	0.76
Weighted mode	0.936	0.744	1.176	0.57
MR-PRESSO model	1.058	0.897	1.247	0.51

Supplementary Table S30. Evaluation of MR associations of lifetime smoking with cataract under different MR models

Method	OR	LCL	UCL	pval
IVW	1.066	0.828	1.372	0.62
MR Egger	1.078	0.375	3.100	0.89
Weighted median	0.904	0.644	1.268	0.56
Weighted mode	0.643	0.287	1.439	0.28
MR-PRESSO model	1.066	0.828	1.372	0.62

Supplementary Table S31. Evaluation of MR associations of alcohol drinks per week with cataract under different MR models

Method	OR	LCL	UCL	pval
IVW	0.858	0.705	1.043	0.12
MR Egger	0.813	0.590	1.119	0.21
Weighted median	0.924	0.698	1.222	0.58
Weighted mode	0.867	0.656	1.147	0.32
MR-PRESSO model	0.897	0.743	1.085	0.27
